# Knowledge attainment, learning approaches, and self-perceived study burnout among European veterinary students

**DOI:** 10.3389/fvets.2024.1292750

**Published:** 2024-07-18

**Authors:** Antti Iivanainen, Carlos Fernando Collares, Jakob Wandall, Anna Parpala, Anne Nevgi, Riikka Keto-Timonen, Andrea Tipold, Elisabeth Schaper, Theo van Haeften, Tina Holberg Pihl, Charles McLean Press, Peter Holm

**Affiliations:** ^1^Department of Biosciences, Faculty of Veterinary Medicine, University of Helsinki, Helsinki, Finland; ^2^Medical Education Unit, Faculty of Medicine and Biomedical Sciences, University of Algarve, Faro, Portugal; ^3^NordicMetrics ApS, Copenhagen, Denmark; ^4^Department of Veterinary and Animal Science, Faculty of Medical and Health Sciences, University of Copenhagen, Frederiksberg, Denmark; ^5^Centre for University Teaching and Learning HYPE, Faculty of Educational Sciences, University of Helsinki, Helsinki, Finland; ^6^Caledonia Hub (Campus Learning and Development Initiatives), Faculty of Educational Sciences, University of Helsinki, Helsinki, Finland; ^7^Department of Food Hygiene and Environmental Health, Faculty of Veterinary Medicine, University of Helsinki, Helsinki, Finland; ^8^Clinic for Small Animals, Neurology, University of Veterinary Medicine Hannover, Foundation, Hannover, Germany; ^9^Centre for E-learning, Didactics and Educational Research, University of Veterinary Medicine Hannover, Foundation, Hannover, Germany; ^10^Department of Biomolecular Health Sciences, Faculty of Veterinary Medicine, University of Utrecht, Utrecht, Netherlands; ^11^Center for Academic Teaching and Learning, Utrecht University, Utrecht, Netherlands; ^12^Department of Veterinary Clinical Sciences, Faculty of Health and Medical Sciences, University of Copenhagen, Taastrup, Denmark; ^13^Department of Preclinical Sciences and Pathology, Faculty of Veterinary Medicine, Norwegian University of Life Sciences, Ås, Norway

**Keywords:** knowledge assessment, structural equation modeling, item response theory, veterinary medical education, learning approaches, study burnout

## Abstract

**Introduction:**

This study investigates the relationship between approaches to learning, self-perceived study burnout, and the level of knowledge among veterinary students. Veterinary educational programs are under regular development and would benefit greatly from detailed feedback on students' knowledge, proficiency, influencing factors, and coping mechanisms.

**Methods:**

The VetRepos consortium developed and calibrated an item repository testing knowledge across the entire veterinary curriculum. Two hundred forty-eight students from seven European veterinary institutions took the VetRepos test, comprising a subset of the repository. They also responded to a questionnaire assessing deep and unreflective learning approaches and self-perceived study burnout, represented by exhaustion and cynicism. Structural equation modeling analyzed the relationship between these latent traits and the VetRepos test score.

**Results:**

The model failed the exact-fit test but was retained based on global fit indices, inter-item residual correlations, and standardized residual covariances. Root Mean Square Error of Approximation with robust standard errors and scaled test statistic was 0.049 (95% confidence interval 0.033–0.071), scaled and robust Comparative Fit Index 0.95 (0.90–0.98), and scaled Standardized Root Mean Square Residual 0.056 (0.049–0.071). Measurement invariance across study years was not violated (ΔCFI = 0.00, χ^2^ = 3.78, Δdf = 4, *p* = 0.44), but it could not be confirmed between genders or universities. The VetRepos test score regressed on the study year [standardized regression coefficient = 0.68 (0.62–0.73)], showed a negative regression on the unreflective learning approach [−0.25 (−0.47 to −0.03)], and a positive regression on the deep approach [0.16 (0.03–0.28)]. No direct association with perceived burnout was observed; however, a significant, medium-sized association was found between the unreflective approach and self-perceived study burnout. No significant differences in learning approaches or perceived burnout were found between study years.

**Discussion:**

The most important source of variance in VetRepos test scores, unrelated to the study year, was the learning approach. The association between the VetRepos test score and self-perceived burnout was indirect. Future research should complement this cross-sectional approach with longitudinal and person-oriented studies, further investigating the relationship between study burnout and learning approaches.

## 1 Introduction

Progress testing (PT) is a longitudinal assessment strategy that has become integral to student assessment in medical education (1). Students take the test several times during their studies. A test typically contains ~100–200 multiple-choice questions or true/false statements covering the entire curriculum. As studies progress, the students' knowledge and proficiency accumulate in parallel with an increase in their test scores. In veterinary medical education, PT is, however, still relatively uncommon. After a pilot project at Utrecht University (2), PT has been implemented to our knowledge only in German-speaking programs in Germany, Austria, and Switzerland (3). In 2019, representatives from veterinary educational establishments in Denmark, Finland, Germany, the Netherlands, Norway, and Sweden, together with the European Association of Establishments for Veterinary Education (EAEVE), formed the VetRepos consortium for creating and calibrating an item repository for progress testing (4). The repository contains 821 test items of varying degrees of difficulty that test the veterinary curriculum, as outlined by EAEVE (5). The VetRepos test uses the unidimensional Rasch model to calculate scores and calibrate item difficulty parameters (6). The item difficulty parameters from the VetRepos test have been calibrated using anchor items to compare the scores across test trials. A thorough description of the VetRepos test can be found in Schaper et al. (4). A single test score per student was used in the current cross-sectional study. Our study does not utilize the longitudinal aspects of PT.

The theoretical basis of deep and unreflective dimensions in the approach to learning stems from the 1970s and 1980s (7–9), and the related scales have been iteratively refined (7–9). Approaches to learning are not psychological states but are learned during schooling (10). The deep approach refers to the focus on meaning during learning; concurrently, the unreflective approach (also called the surface approach) refers to students' focus on memorizing details without attempting to understand the meaning of the subject (11, 12). Although various other approaches to learning and studying, including, e.g., strategic, achieving, or organized approaches, have been identified and characterized over the decades (9), we decided to focus on the deep and unreflective approaches. These fundamental learning approaches have been replicated in several studies and are widely accepted in the community of pedagogical scientists (7, 9, 13–15).

Burnout at work is an occupational stress-related affective response encompassing exhaustion, cynicism, and feelings of inadequacy (16). Similar dimensionality has also been discovered in the perception of burnout related to school or university studies (17, 18). The perception of burnout is common among students in medicine as well as in veterinary medicine, with a >40% prevalence (19, 20).

The unreflective approach to learning has been associated with self-perceived study burnout: students with an unreflective (superficial) approach experienced more exhaustion than students with a deep approach to learning, and students with a deep approach to learning experienced less exhaustion than other student groups (13, 21). The deep approach is associated with study success as measured by grade point average (GPA) or similar metrics (22, 23) that depend on an individual test, study program, or university.

The purpose of the present study is to investigate the use of VetRepos test scores as a simple measure of study success across European veterinary schools in the VetRepos consortium and to illustrate the relationships between the VetRepos test score, deep and unreflective approaches to learning, and self-perceived study burnout. In addition, we hope to identify patterns between these variables, which warrant additional research.

## 2 Methods

### 2.1 Data collection

As a part of a progress test development project, “VetRepos,” between European establishments of veterinary education (4, 24), veterinary students from seven European universities were invited to participate in a trial test (hereafter, the VetRepos test) that was open from June to August 14, 2023. The test comprised questions across the veterinary curriculum as outlined by EAEVE (5). Students were also invited to fill out an electronic questionnaire with 17 statements on approaches to learning and self-perceived study burnout (hereafter, the study questionnaire).

The trial was announced using email lists, in-house news bulletins, and face-to-face discussions on various occasions where students gathered. Participation in the study was voluntary. The students were free to participate in the study at any time between the opening and closing of the VetRepos test and the study questionnaire. There was no time limit for their participation. The participants were instructed not to prepare specifically for the test or use additional reference materials. Whether the participants had prepared for the test or used additional materials was not controlled.

### 2.2 Data protection regulation

The European Union's General Data Protection Regulation 2016/679 (GDPR) was strictly followed during all study phases. Before being allowed to proceed with the VetRepos test or the study questionnaire, the students were informed about the purpose of the study, the methods of data storage and handling, and their rights, including the right to be forgotten (25) and to withdraw their consent at any time. Only those students who provided explicit consent were allowed to proceed with the VetRepos test or the study questionnaire. The data were collected via a secure web interface, and both the VetRepos test questions and study questionnaire statements were hosted on QualtricsXM at Utrecht University, ensuring compliance with GDPR standards (26).

### 2.3 The respondents

The data comprised responses from 257 veterinary students who filled out the study questionnaire and participated in the VetRepos test. Twelve students had participated in a similar VetRepos test earlier (see Section 2.4) and filled out only the study questionnaire. Nine respondents with incomplete data on the study questionnaire were removed, leaving the responses of 248 students from Denmark, Finland, France, Germany, the Netherlands, Norway, and Sweden for the analyses.

The number of students between the universities ranged from 5 to 105 (Table 1A). A total of 166 (66.94%) students were in their first 3 years of their studies. The data included 29 male and 217 female students. Two students entered a gender category, “other.” The distribution of the students across study years and between women and men is presented in Table 1B.

**Table 1:** Distribution of students across universities, study years and genders.

**A. Number of students per university**							
**University**							**Sum**
**A**	**B**	**C**	**D**	**E**	**F**	**G**	
31	32	27	18	5	105	30	**248**
**B. Gender distribution**							
**Gender**	**Study year**						**Sum**
	**1**	**2**	**3**	**4**	**5**	**6**	
Male	4	8	3	6	5	3	**29**
Female	44	64	41	33	23	12	**217**
Sum	**48**	**72**	**44**	**39**	**28**	**15**	**246**
**C. Ratio of observed/expected number of students**							
**University**	**Study year**						
	**1**	**2**	**3**	**4**	**5**	**6**	
A	1.14	0.44	1.27	1.23	1.71	0.53	
B	0.32	0.32	1.23	1.39	1.38	4.13	
C	0.00	0.38	1.04	2.59	1.64	1.84	
D	0.28	1.51	0.63	0.71	1.97	0.92	
E	0.00	0.68	1.13	2.54	0.00	3.31	
F	1.88	1.36	0.86	0.30	0.25	0.00	
G	0.00	1.36	1.13	1.27	1.48	0.55	

(B) To ensure privacy, two students with the gender category “other” are not shown. (C) The distribution of students per study year differs between University F and all other universities at α = 0.01, and between Universities B and G at α = 0.05.

The participants from University F included noticeably many 1st and 2nd year students. The distribution of study years differed between students from University F vs. the other universities (Table 1C, χ^2^-test: χ^2^ statistic = 116.43, simulated *p*-value = 0.00050 with 2,000 iterations, followed by pairwise χ^2^-tests at α = 0.01). Study year distribution differed also between Universities B and G at α = 0.05 (χ^2^ = 12.95).

### 2.4 VetRepos test score

The data comprised responses to single-best-answer multiple-choice questions and true/false type statements based on additional questions or vignettes (4). The VetRepos items test knowledge across the entire veterinary curriculum, covering a range of disciplines in four subscales (17 disciplines in the basic sciences, 10 in companion animal and equine clinical sciences, 12 in animal production and production animal clinical sciences, and five in food safety and quality, public health, and one health concept). Each test trial contained 28 anchor items for test equating purposes so that the scores could be interpreted similarly throughout different test applications. The total number of items was 161 for the 245 students who took the test during the summer. The number of items ranged between 110 and 216 for the other 12 students who had taken the test earlier. The responses were scored as one (1) point for a correct answer and zero (0) points for an incorrect answer. Rasch model analysis (6) software RUMM (27) was used to place the student ability score (θ) and the item difficulty on a single linear logit scale where the mean item difficulty has a value of zero. The range of item difficulty for the items in the VetRepos database covered approximately six logit units (4).

The logit scale was linearly transformed, as has been done in the large-scale assessment studies by the International Association for the Evaluation of Educational Achievement (28, 29). The resulting VetRepos integral scale has a mean value of 500 and a standard deviation of 100 and is easier to interpret than the logit scale. This score is based on the entire VetRepos project database, which includes 1,948 respondents from the six VetRepos tests. Because of the shared anchor items, simple comparisons can be made between scores from any of the six trials. The Person Separation Index (PSI = 0.86, 95% confidence interval = 0.85–0.87) was used to estimate the reliability of the model. PSI is a more appropriate reliability estimate in Rasch model analyses than Cronbach's α coefficient, as it uses logit scores rather than raw scores for its calculation, even though they can be interpreted in the same manner. Furthermore, ~80% of the observations contained missing values, which Cronbach's α is sensitive to. The details of the development and structure of the VetRepos test are described in Schaper et al. (4).

### 2.5 Instruments

We used 17 items from the HowULearn (HUL, previously Learn (15, 30)] project questionnaire of the University of Helsinki to measure the students' approaches to learning and their perception of study burnout (Supplementary Table 1). All the study questionnaire statements were responded to using the Likert scale: “I completely disagree” (0), “I disagree” (1), “I neither agree nor disagree” (2), “I agree” (3), and “I completely agree” (4).

#### 2.5.1 Approaches to learning

Eight items measuring deep vs. unreflective learning approaches (Supplementary Table 1) were partly derived from the Enhancing Teaching-Learning Environments project (8, 31). The original surface approach subscale has been renamed the unreflective approach (12). The items were tested using a large cohort of university students from Denmark, Finland, and the UK (12, 32, 33). An example statement measuring the deep approach reads, “Ideas and perspectives I've come across while I'm studying make me contemplate them from all sides,” in contrast to a statement measuring the unreflective approach, such as “Much of what I've learned seems no more than unrelated bits and pieces.”

**Figure 1:**
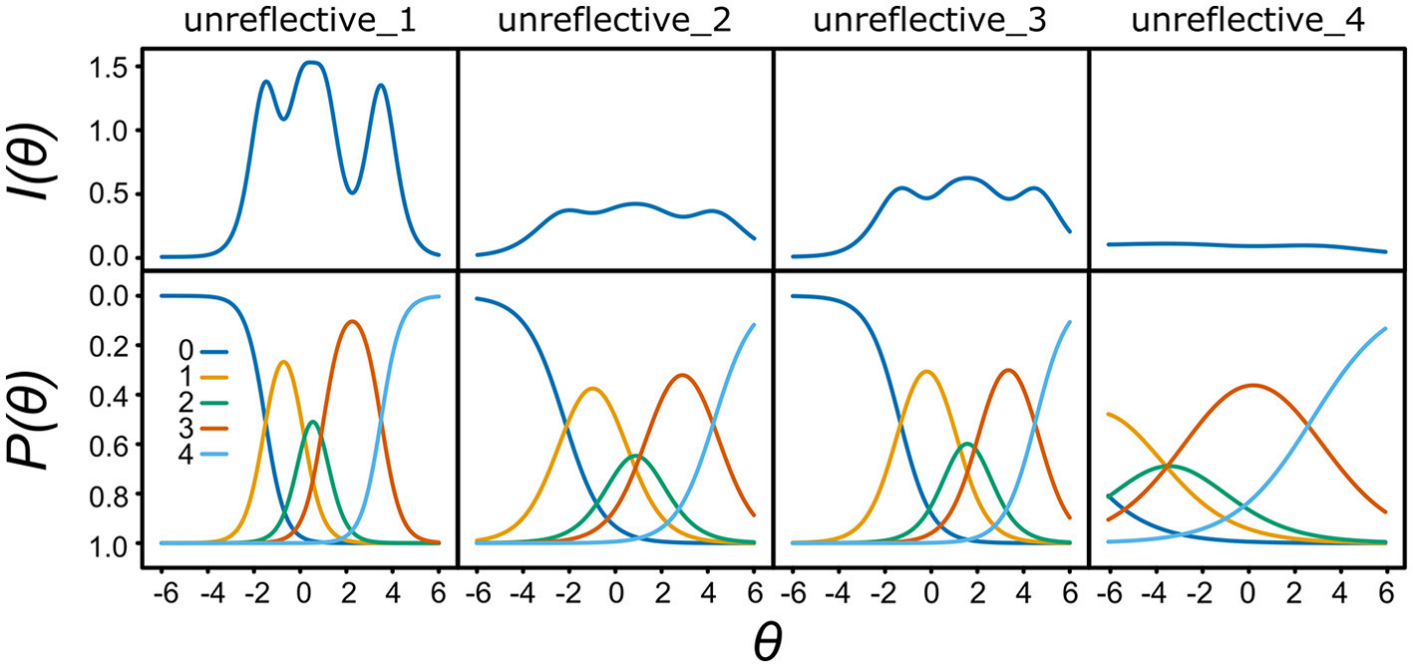
Information content (*I*) and probability (*P*) as functions of the level of the unreflective approach (θ). unreflective_1–4: indicator items for the unreflective approach. The Likert scale key (0–4) is indicated for *P(*θ*). N* = 248.

Inspection of pairwise Spearman correlation coefficients between the items revealed that item unreflective_4 (“Often I have to repeat things in order to learn them”) differed from the rest of the indicators of the unreflective approach (Supplementary Tables 2A, B). In addition, confirmatory factor analysis revealed a low factor loading of 0.28 (0.15–0.40) for item unreflective_4 (Table 2). In exploratory factor analysis, this item loaded similarly onto two factors (Table 2). Additional evidence about the inadequacy of item unreflective_4 to measure the purported construct was further suggested by the results of a polytomous Rasch model analysis (Figure 1), as it was not able to differentiate between participants with different levels of the unreflective approach, consequently yielding visibly less information than the other items. Based on the above, responses to item unreflective_4 were removed from the data. The removal of the item slightly increased the Cronbach's α reliability coefficient from 0.61 (0.52–0.68) to 0.64 (0.56–0.71) for the unreflective approach scale (Table 3).

**Table 2:** Factor loadings on the learning approach indicator items.

	**CFA**			**EFA, factor 1**			**EFA, factor 2**		
	**ci.lower**	**f.cfa**	**ci.upper**	**ci.lower**	**f1.efa**	**ci.upper**	**ci.lower**	**f2.efa**	**ci.upper**
deep_1	0.38	0.50	0.62	0.39	0.51	0.62	−0.11	0.02	0.16
deep_2	0.24	0.37	0.50	0.25	0.38	0.51	−0.12	0.02	0.17
deep_3	0.73	0.82	0.92	0.74	0.82	0.91	−0.09	−0.02	0.06
deep_4	0.73	0.82	0.92	0.73	0.81	0.90	−0.06	0.00	0.06
unreflective_1	0.61	0.75	0.88	−0.04	0.01	0.05	0.63	0.77	0.90
unreflective_2	0.41	0.54	0.66	−0.17	−0.04	0.09	0.37	0.51	0.65
unreflective_3	0.43	0.56	0.69	−0.13	−0.02	0.10	0.40	0.54	0.67
unreflective_4	0.15	0.28	0.40	0.14	0.28	0.42	0.24	0.39	0.53

Standardized factor loadings from confirmatory factor analysis (f_deep, f_unreflective) on column f.cfa. Factor loadings after exploratory two-factor analysis with geomin rotation on f1.efa and f2.efa. ci.lower, ci.upper = lower and upper bounds of the 95% confidence interval. Factor loadings between −0.2 and 0.2 are colored gray.

**Table 3:** Reliability estimates: Cronbach's α with 95% Feldt confidence intervals for the various scales, McDonald's ω_tot_ for the entire measurement model, and Person Separation Index for the VetRepos test.

**Target**	**Reliability estimate**	**Lower_ci**	**Estimated value**	**Upper_ci**
Deep scale		0.66	0.72	0.77
Unreflective4 scale		0.52	0.61	0.68
Unreflective scale	Raw Cronbach's α	0.56	0.64	0.71
Cynicism scale		0.76	0.80	0.84
Exhaustion scale		0.67	0.73	0.78
Inadequacy scale		0.42	0.55	0.65
Measurement model	McDonald's ω_tot_	0.77	0.81	0.84
VetRepos test	pers.sep.index	0.85	0.86	0.87

Gray = scales that were discarded and not used in modeling. Indicator items (see Supplementary Table 1) unreflective1–4 are included in the unreflective4 scale and unreflective1–3 in the unreflective scale.

lower_ci, lower bound of 95% confidence interval; upper_ci, upper bound of 95% confidence interval; pers.sep.index, Person Separation Index.

#### 2.5.2 Self-perceived study burnout

We utilized the nine-item School Burnout Inventory (SBI-9) (17), which has been used to investigate self-perceived burnout among university students [see e.g., (13)]. The following statements from SBI-9 represent each dimension in turn (Supplementary Table 1): “I brood over matters related to my studies during my free time” (exhaustion), “I feel that I am losing interest in my studies” (cynicism), and “I often have feelings of inadequacy in my studies” (inadequacy).

As the original factor structure of SBI-9 has not been optimal for some datasets (34–36), we decided to explore the factor structure of this scale in our data. Exploratory factor analysis indicated that the indicator items for inadequacy did not measure a single factor in our data (Table 4). We also observed a low-reliability coefficient for inadequacy [Cronbach's α = 0.55 (0.42–0.65)] in our data (Table 3). Based on the equivocal factor structure and the low reliability of the inadequacy subscale, we decided to remove the responses to the two inadequacy items from our data. The scales on cynicism and exhaustion were retained unaltered despite the mixed factor loadings on cynicism_1 (Table 4).

**Table 4:** Factor loadings from exploratory factor analysis of SBI-9 using the complete set of unweighted observations (*N* = 248).

	**3-factor solution**			**2-factor solution A**		**2-factor solution B**	
	**dim3_f1**	**dim3_f2**	**dim3_f3**	**dim2A_f1**	**dim2A_f2**	**dim2B_f1**	**dim2B_f2**
cynicism_1	0.56	0.26	0.01	0.50	0.34	0.57	0.24
cynicism_2	0.88	−0.10	0.01	0.76	0.10	0.82	0.04
cynicism_3	0.89	0.01	−0.22	0.86	−0.08	0.83	−0.12
exhaustion_1	0.00	0.66	0.19	0.01	0.73	0.13	0.55
exhaustion_2	0.03	0.01	0.64	0.01	0.61	0.03	0.67
exhaustion_3	−0.08	0.03	0.60	−0.12	0.60	−0.08	0.61
exhaustion_4	0.01	−0.12	0.80	−0.01	0.61	−0.01	0.69
inadequacy_1	0.16	0.67	0.00	0.16	0.58	NA	NA
inadequacy_2	0.43	0.21	0.00	0.39	0.25	NA	NA

Geomin rotation method. dim3_f1–3 = three-factor solution, dim2A_f1–2 = two-factor solution, dim2B_f1–2 = two-factor solution without the inadequacy items. Indicator items appear as row names. Loadings between −0.2 and 0.2 are colored gray. NA, not available.

#### 2.5.3 Reliability of the measurement model

McDonald's ω_tot_ was used as the reliability coefficient for the measurement model comprising the four factors and their 14 indicator items (Table 3). McDonald's ω_tot_ is suited for multidimensional data (37, 38) and thus complements reliability assessments of the individual unidimensional scales that were based on the coefficient Cronbach's α.

### 2.6 Structural equation modeling

The data were not normally distributed. The VetRepos test score distribution was platykurtic [kurtosis = −0.75 (−0.98, −0.48), Supplementary Table 3A] and failed the Shapiro-Wilk normality test (*W* = 0.99, *p*-value = 0.013). The scales failed the multivariate Mardia normality test (Supplementary Table 3B). Thus, the maximum likelihood estimation of model parameters was extended by the estimation of robust errors and a robust χ^2^ statistic, as suggested by the *lavaan* manual (39). The maximum likelihood-based χ^2^ statistic was rescaled by the Satorra-Bentler correction factor 1.074. These modifications were implemented by specifying estimator = “MLM” in the lavaan() command.

### 2.7 Adjusting the data by down-sampling and applying a weight matrix

To mitigate the potential disproportionate effect of University F on 105 (42.34%) of the observations, we repeated the modeling on two sets of adjusted data. *The down-sampled dataset* contained 1,000 iterations of 173 observations, of which 30 observations (17.34%) were randomly selected from University F. *The weighted dataset* was modified with the help of a weight matrix, where the weights were inversely proportional to the relative number of observations from each university. Five students from University E were removed as their weight constant would have been 7.09, with potentially distorting effects on the dataset.

### 2.8 Scripting

The analyses in this study were conducted in R version 4.3.3 (40) with the help of the following R packages: *lavaan* (39), *mirt* (41), and psych (42). The pdf version of the documented custom R script with the complete list of the required packages is attached as a Supplementary material (R-code.pdf). The script is available at https://dx.doi.org/10.6084/m9.figshare.25470436.

## 3 Results

### 3.1 Distribution of VetRepos test scores

**Figure 2:**
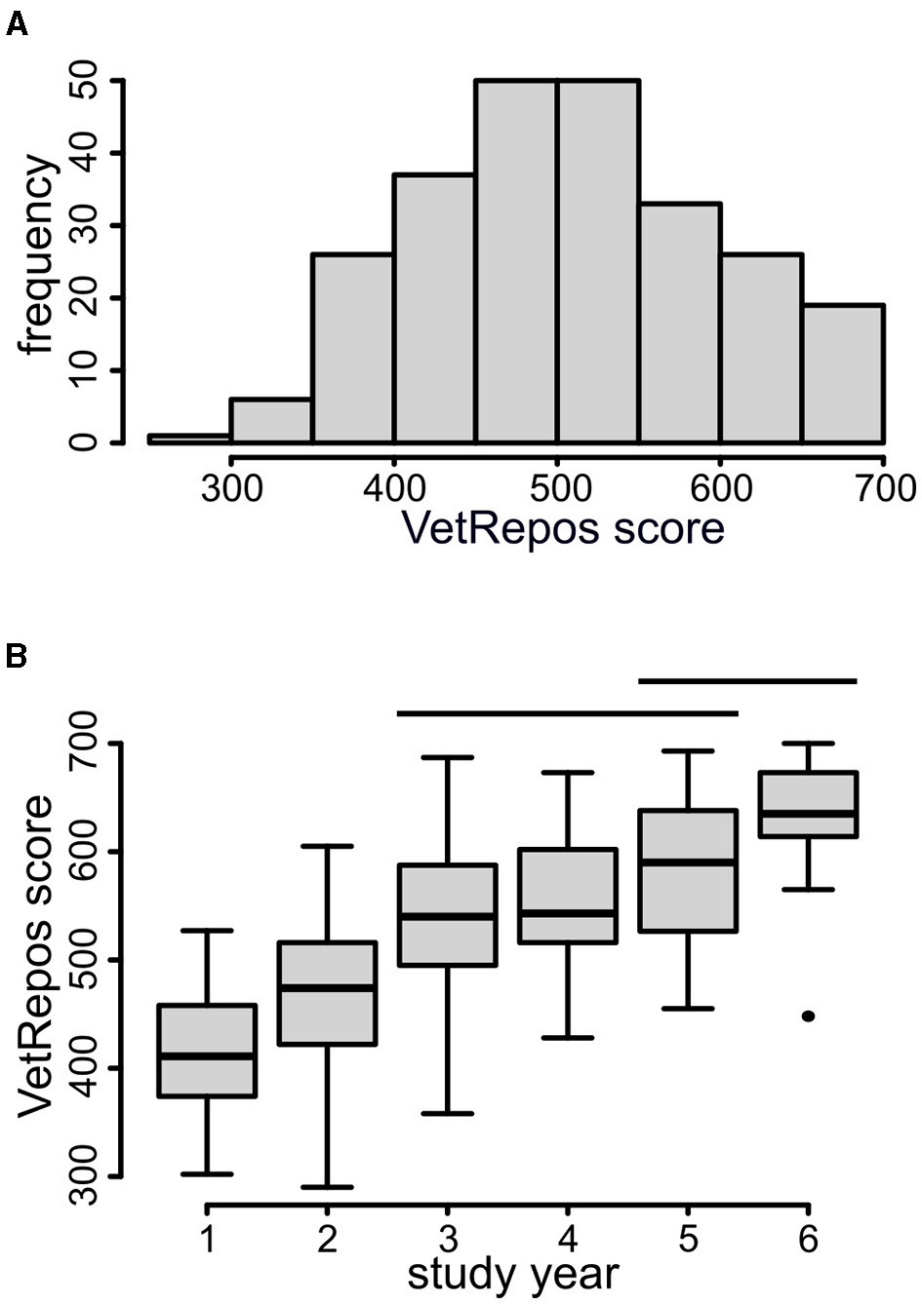
VetRepos test score. **(A)** The distribution of the VetRepos test score in the study population (*N* = 248). **(B)** Box plot of the VetRepos test score in each study year. The horizontal bars indicate groups with non-significant differences in the mean value (Kruskal test with Dunn's *post hoc* test for the pairwise comparison of the means). The horizontal bars in the boxes indicate median values. *N* = 248.

The median VetRepos test score in the current dataset was 508 (*N* = 248), with a range from 290 to 700. The mean value was 507.48, with a standard deviation of 92.51. The distribution of the scores is shown in Figure 2A. As expected, there was an increasing trend in the scores with an increase in study years (Figure 2B).

### 3.2 Interitem correlations, reliability indices, and the indicator sum scores for deep and surface learning approaches and self-perceived burnout

**Figure 3:**
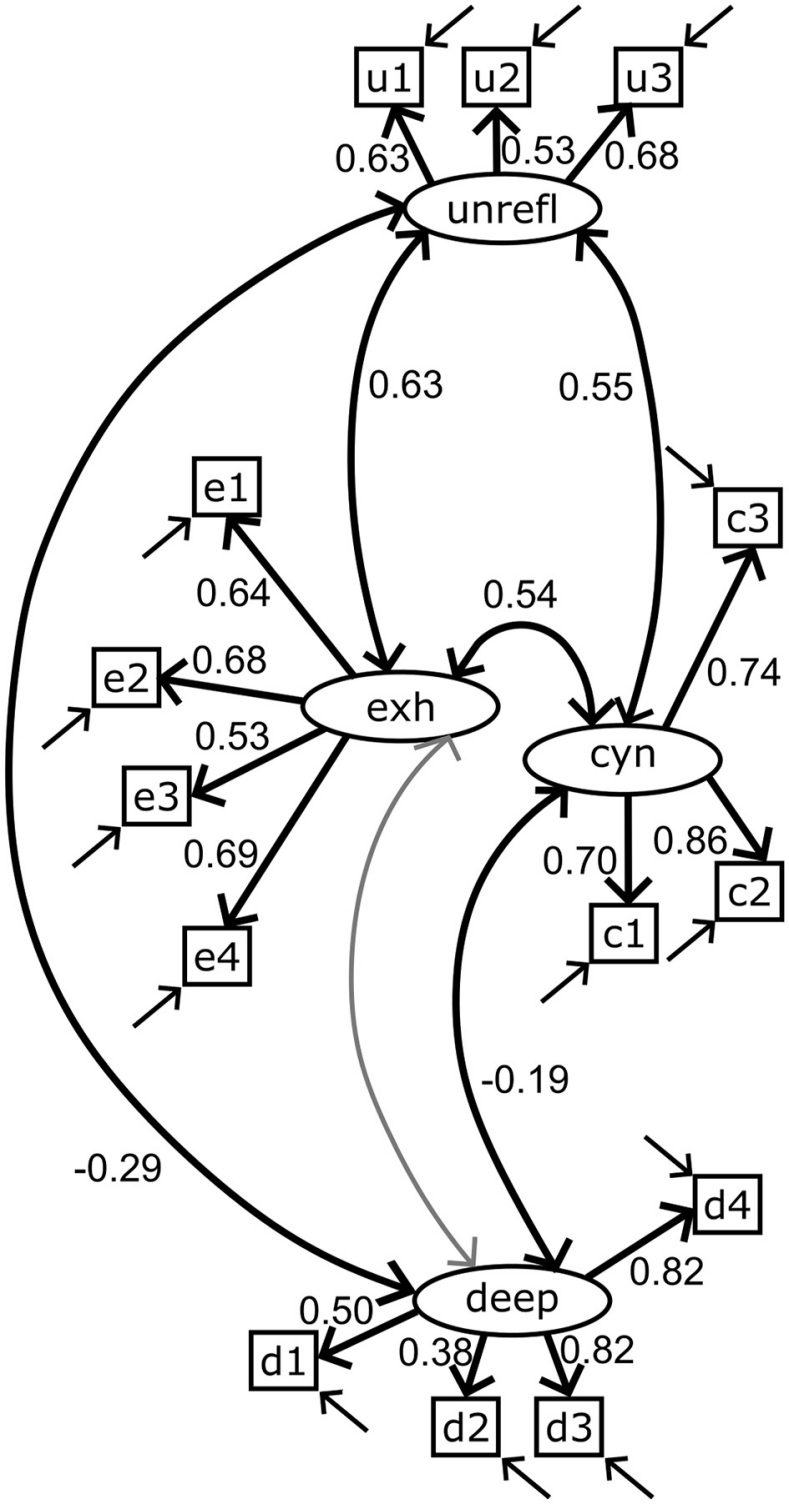
Path diagram of the measurement model. The four factors and their indicator items are deep approach (deep, d1–4), unreflective approach (unrefl, u1–3), cynicism (cyn, c1–3), and exhaustion (exh, e1–4). Straight single arrow: factor loadings. Bidirectional curved arrow: correlation. Standardized factor loadings and correlation coefficients are shown. The gray color indicates a non-significant relationship (α = 0.05). Errors on the indicator items are indicated by arrows, but the error variables remain hidden. A complete list of estimated coefficients is presented in Supplementary Table 4.

The interitem bivariate Spearman correlation coefficients for all indicator item pairs are shown in Supplementary Tables 2A, B. The Cronbach's α reliability coefficient varied between 0.64 and 0.80 for the final set of scales used in the modeling (Table 3). The measurement model of the four scales (deep and unreflective approaches and perceived cynicism and exhaustion) was fitted using CFA. Although the model failed the exact-fit test ($\chi_{\text{MLM}}^{2}$ = 113.62, df = 71, *p* = 0.001), global fit statistics suggested retaining the model (Table 5A) (43). The fitted model was used to estimate McDonald's ω_tot_ = 0.81 with a 95% bootstrapped confidence interval = (0.76–0.84; 1,000 iterations, Table 3). A path diagram and the standardized factor loadings of the measurement model are presented in Figure 3. A complete list of the standardized coefficients of the measurement model is available in Supplementary Table 4A.

**Table 5:** Global fit index estimates and their bootstrapped 95% confidence intervals for the measurement (Figure 3) and full structural equation models (Figure 5).

	**Measurement model**			**Full structural equation model**		
	**Lower_ci**	**Estimate**	**Upper_ci**	**Lower_ci**	**Estimate**	**Upper_ci**
**A**.						
rmsea.robust	0.031	0.051	0.076	0.033	0.049	0.071
cfi.robust	0.89	0.95	0.98	0.90	0.95	0.98
tli.scaled	0.86	0.93	0.98	0.87	0.94	0.97
srmr	0.045	0.054	0.068	0.049	0.056	0.071
**B**.						
rmsea.robust	0.043	0.060	0.093	0.038	0.051	0.083
cfi.robust	0.86	0.94	0.97	0.87	0.95	0.97
tli.scaled	0.81	0.92	0.96	0.83	0.93	0.96
srmr	0.050	0.059	0.080	0.052	0.059	0.079
**C**.						
rmsea.robust	0.037	0.050	0.062	0.029	0.043	0.056
cfi.robust	0.93	0.95	0.97	0.93	0.96	0.98
tli.scaled	0.90	0.94	0.96	0.91	0.95	0.98
srmr	0.057	0.062	0.068	0.057	0.063	0.068

**(A)** Complete unweighted data (N=248). **(B)** Weighted data (N = 243). **(C)** The estimate is a mean value of 1,000 iterations. Down-sampled data (N = 173).

lower_ci, lower bound of the 95% confidence interval; upper_ci, upper bound of the 95% confidence interval.

**Figure 4:**
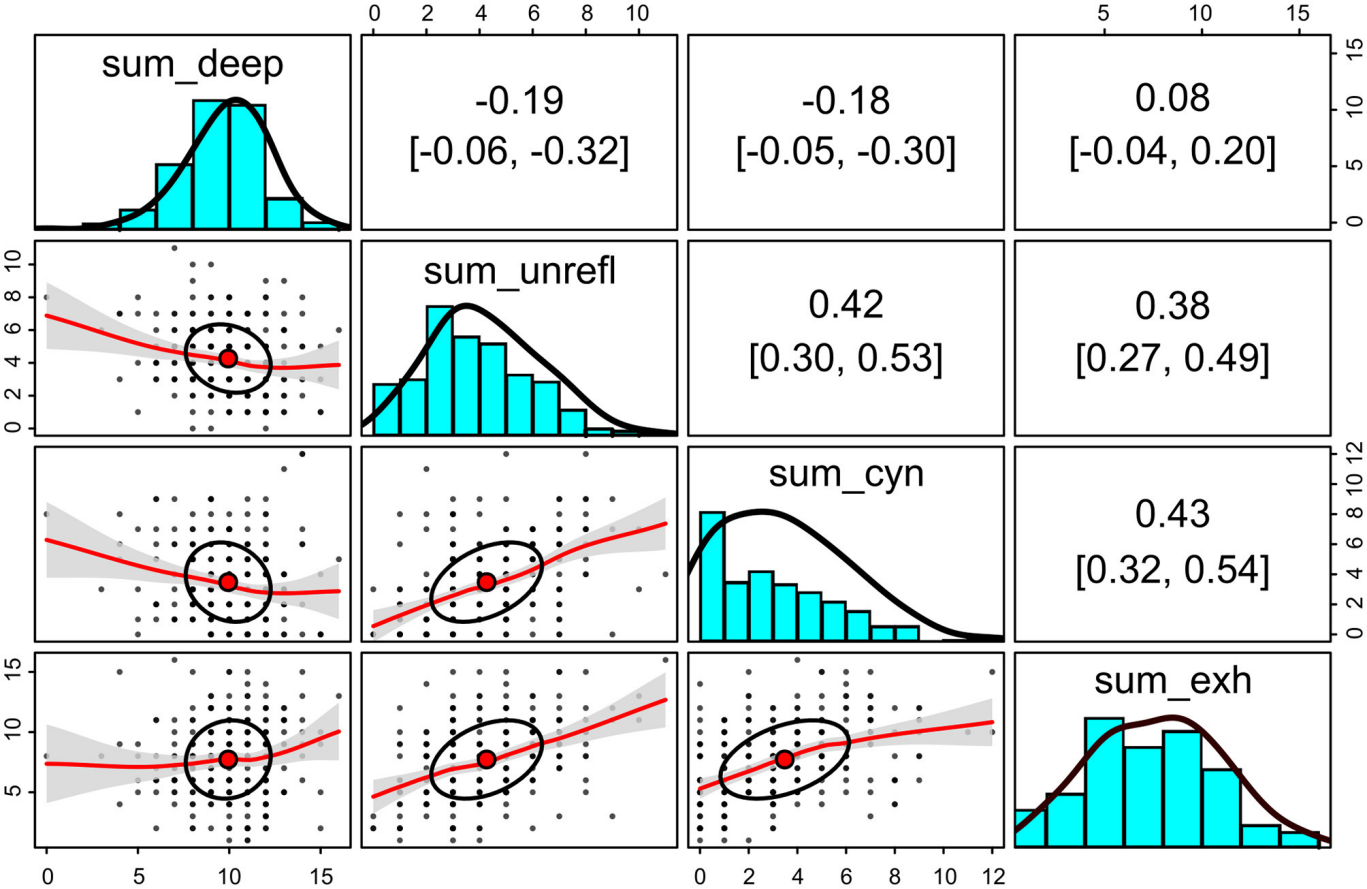
Sum scores of the indicator items for deep and unreflective approaches to learning and for the self-perceived study burnout for each participant. Upper triangle: bivariate Spearman correlation coefficients with bootstrapped 95% confidence intervals (2,000 iterations). Lower triangle: bivariate scatter plots with correlation ellipses and loess fit with confidence intervals (α = 0.05). Diagonal: histograms with density plots. The raw scores are on the *x*-axis and the counts on the *y*-axis. *N* = 248.

The responses to the indicator items as sum scores are compared in Figure 4. A positive correlation of 0.47 (95% bootstrapped confidence interval with 1,000 iterations: 0.36–0.57) was observed between the sum scores of the unreflective approach (unreflective_1–3) and the perceived study burnout indicators (not shown).

### 3.3 Structure and global and local fit of the full structural equation model

**Figure 5:**
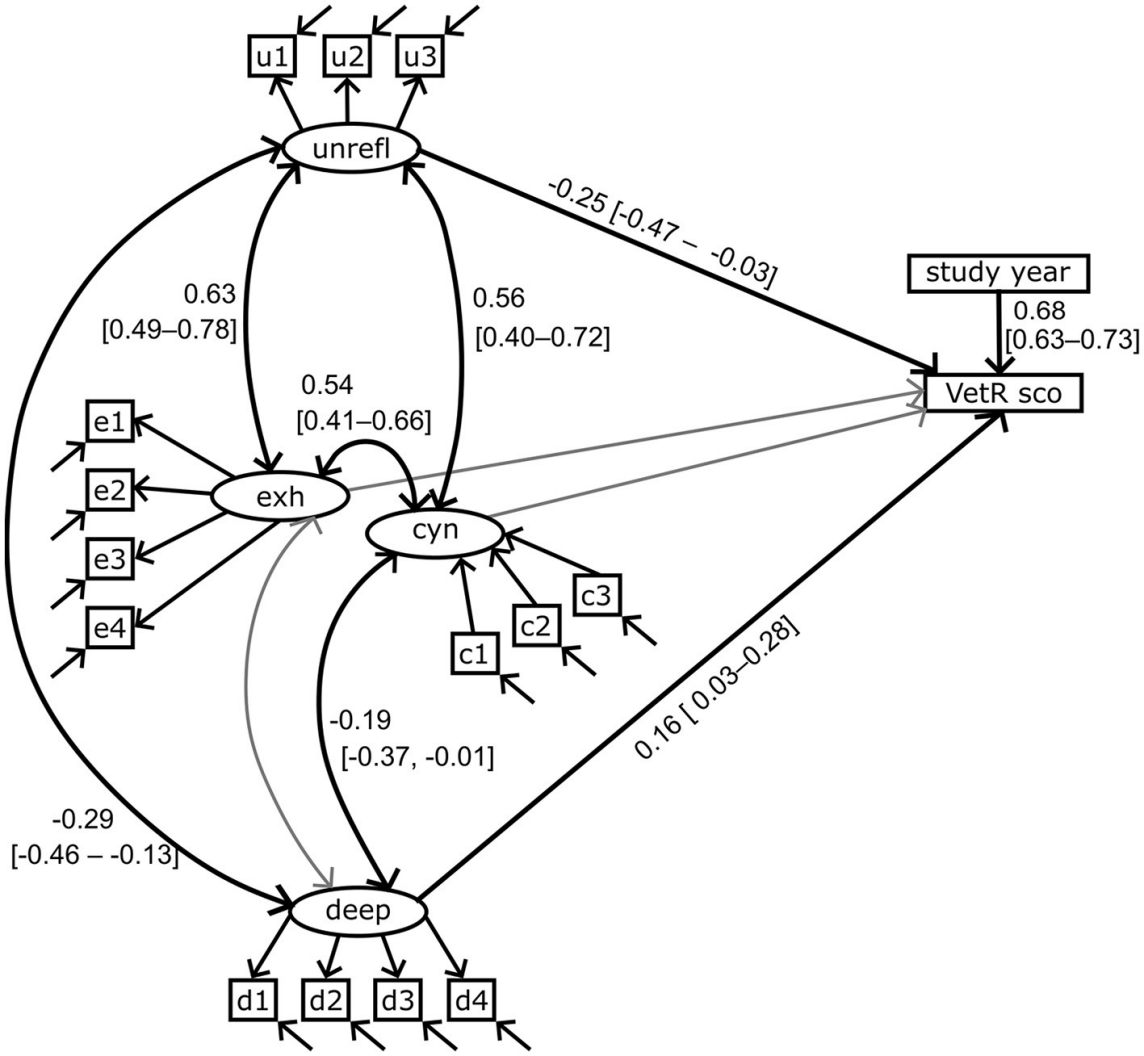
Path diagram of the full structural equation model. The four factors and their indicator items are deep approach (deep, d1–4), unreflective approach (unrefl, u1–3), cynicism (cyn, c1–3) and exhaustion (exh, e1–4). VetR sco, VetRepos test score. Statistically significant (α = 0.05) standardized covariances and regression coefficients with 95% confidence intervals are shown. Bidirectional curved arrow: correlation. Straight single arrow: regression. The gray color indicates a non-significant relationship. Errors on the indicator items are indicated by arrows, but the error variables remain hidden. A complete list of estimated coefficients is presented in Supplementary Table 7.

**Figure 6:**
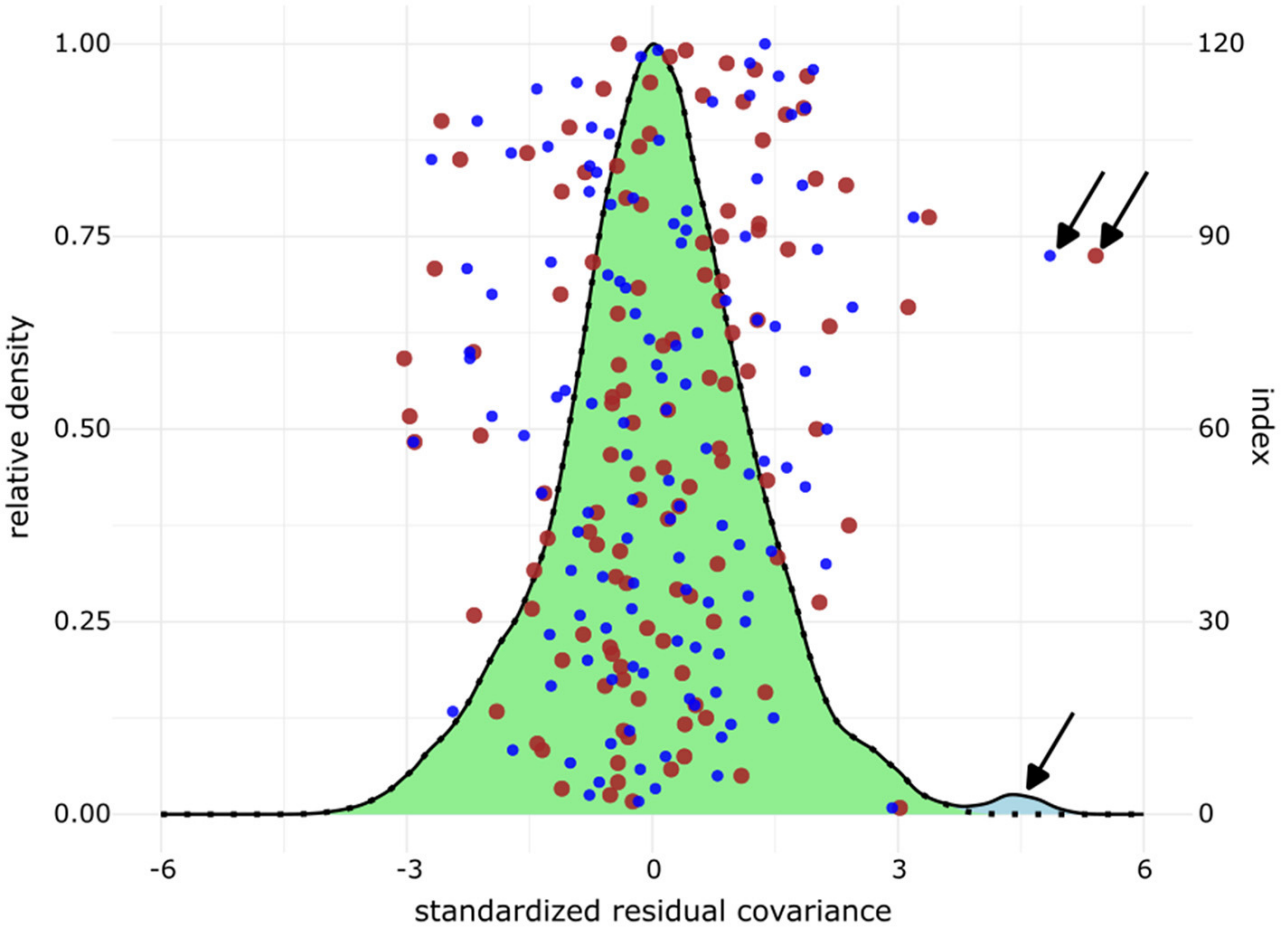
Standardized interitem residual covariances of the full structural equation model. Small blue dots = unweighted data (*N* = 248 observations). Large brown dots = weighted data (*N* = 243 observations). Density plot with a light blue area under the curve = down-sampled data (173 observations, 1,000 iterations). Dotted density plot with a light green area under the curve = down-sampled data with a mask on the residual between cynicism_1 and exhaustion_1. For the down-sampled data, the mean residual between cynicism_1 and exhaustion_1 was 4.44, 2.5% quantile = 3.80, 97.5% quantile = 5.02, and range = 3.44–5.29. *x*-axis: residuals, left *y*-axis: relative density, right *y*-axis: index from the residual data frame. Arrows mark the residual covariance between cynicism_1 and exhaustion_1.

A path diagram of the model with standardized coefficients is shown in Figure 5. The model failed the exact-fit test ($\chi_{\text{MLM}}^{2}$ = 148.20, df = 95, *p*-value = 0.00039). As the χ^2^ test is easily significant with larger sample sizes and more complex models, other global fit indices were computed (Table 5A). The robust RMSEA was 0.049 (95% ci = 0.033–0.072), robust CFI = 0.95 (0.90–0.98), scaled TLI = 0.94 (0.87–0.97), and SRMR = 0.056 (0.049–0.071). The global indices are compatible with retaining the model (43). We then assessed the local fit by inspecting the interitem residual correlation coefficients and standardized residual covariances (Supplementary Tables 5A and 6A). All residual correlation coefficients were between −0.3 and 0.3. The standardized residual covariances are plotted in Figure 6. The highest value of standardized residual covariance was 4.86 (between items cynicism_1 and exhaustion_1). The Shapiro–Wilk normality test statistic (*W* = 0.99, *p* = 0.35) was compatible with the normality of the residuals. Based on the global fit indices and the local fit, we retained the model despite its failure to meet the exact-fit test and the large standardized residual covariance between cynicism_1 and exhaustion_1.

### 3.4 Estimated coefficients

All the estimated standardized coefficients from the fitted model are presented in Supplementary Table 7A. Except for the loading on item deep_2, all the factor loadings are >0.4. The communalities (squared standardized loadings) explain more than 50% of the variance in the indicators deep_3, deep_4, cynicism_2, and cynicism_3. Communalities for the indicators of exhaustion and the unreflective approach range from 0.28 to 0.46 (95% ci = 0.17–0.61).

The size of the observed regression coefficients ranged from small to very large, as determined by the criteria discussed in Funder and Ozer (44). The VetRepos test score regressed on the deep learning approach with a small coefficient of 0.16 (95% ci = 0.03–0.28) and on the unreflective approach with a medium-sized coefficient of −0.25 (−0.47 to −0.03; Figure 5, Supplementary Table 7A). As expected, a large regression coefficient [0.68 (0.62–0.73)] on the study year was also observed (44).

The unreflective learning approach correlated with cynicism [*r* = 0.56 (0.40–0.72)] and exhaustion [*r* = 0.63 (0.49–0.78)]. Cynicism correlated with exhaustion [*r* = 0.54 (0.41–0.66)]. We also observed a small negative correlation coefficient between the deep learning approach and cynicism [*r* = −0.19 (−0.01 to −0.37); Figure 5, Supplementary Table 7A].

### 3.5 Measurement invariance

#### 3.5.1 Study year

We assessed measurement invariance with multiple indicators and multiple-causes (MIMIC) modeling by comparing the fits of a constrained vs. an unconstrained model. The regression coefficients of the latent traits (deep approach, unreflective approach, cynicism, and exhaustion) during the study year were freely estimated in the unconstrained model. There was no difference in the robust CFI global fit metric between the models (cfi.robust = 0.942), and the scaled χ^2^-difference test (45, 46) was non-significant (Δχ^2^ = 3.78, Δdf = 4, *p*-value = 0.44, Table 6A). Together, these results argue for measurement invariance between the study years in the full structural equation model (47, 48).

**Table 6:** Akaike and Bayesian information criterion and scaled χ^2^-difference test between the constrained and unconstrained models.

	**df**	**AIC**	**BIC**	**Chisq**	**Chisq diff**	**df diff**	**Pr (>Chisq)**
**A**.							
fit_unconstrained_syear	93	7,359.25	7,506.81	166.64	NA	NA	NA
fit_constrained_syear	97	7,355.03	7,488.54	170.42	3.78	4	0.44
**B**.							
fit_unconstrained_gender	108	5,738.78	5,886.34	178.68	NA	NA	NA
fit_constrained_gender	112	5,754.03	5,887.54	201.94	19.35	4	0.00067

The regression coefficients of the latent traits for the study year were freely estimated in the unconstrained model.

df, degrees of freedom; AIC, Akaike information criterion; BIC, Bayesian information criterio; Chisq, standard χ^2^ test statistic; NA, not available.

**(A)** The regression coefficients of the latent traits for the study year were freely estimated in the unconstrained model. **(B)** The regression coefficients of the latent traits on the binary gender (male, female) were freely estimated in the unconstrained model.

#### 3.5.2 Gender and university

Measurement invariance between men and women was analyzed by treating gender as a numerical variable (0 for men and 1 for women) in the MIMIC models. Invariance was rejected because of a large difference in robust CFI metrics (ΔCFI = ^−^0.013) (47, 48) and a significant scaled χ^2^-difference test (Δχ^2^ = 19.35, Δdf = 4, *p*-value = 0.00067, Table 6B). Measurement invariance could not be assessed between universities for several reasons, including the small sample sizes in each university, which prevented measurement alignment, and the absence of some categories in the responses to some items in some groups, preventing the use of multiple group confirmatory factor analysis.

### 3.6 Analyses of down-sampled and weighted data

The comparison between models fitted on the original complete (*N* = 248), weighted (*N* = 243), and down-sampled data (*N* = 173, 1,000 iterations) was carried out on the global and local fit and by comparing model estimated parameter values. All the fitted models failed the exact test. The global fit indices (rmsea.robust, cfi.robust, tli.scaled, and srmr) were comparable between models fitted on the different datasets (Tables 5A–C). The local fit was assessed by the distribution of residuals. The interitem residual correlations ranged between −0.23 and 0.31 for all datasets (see Supplementary Tables 5A, B for exact values for unweighted complete and weighted datasets). The standardized residual covariances ranged between −4.36 and 5.29 (1,000 down-sampled datasets). The most extreme and larger-than-expected standardized residual covariance value was always observed between items cynicism_1 and exhaustion_1 regardless of the data that were fitted to the full SE model (Figure 6, Supplementary Tables 6A, B). Based on the above-described results, we conclude that the model fits were comparable between the datasets.

**Figure 7:**
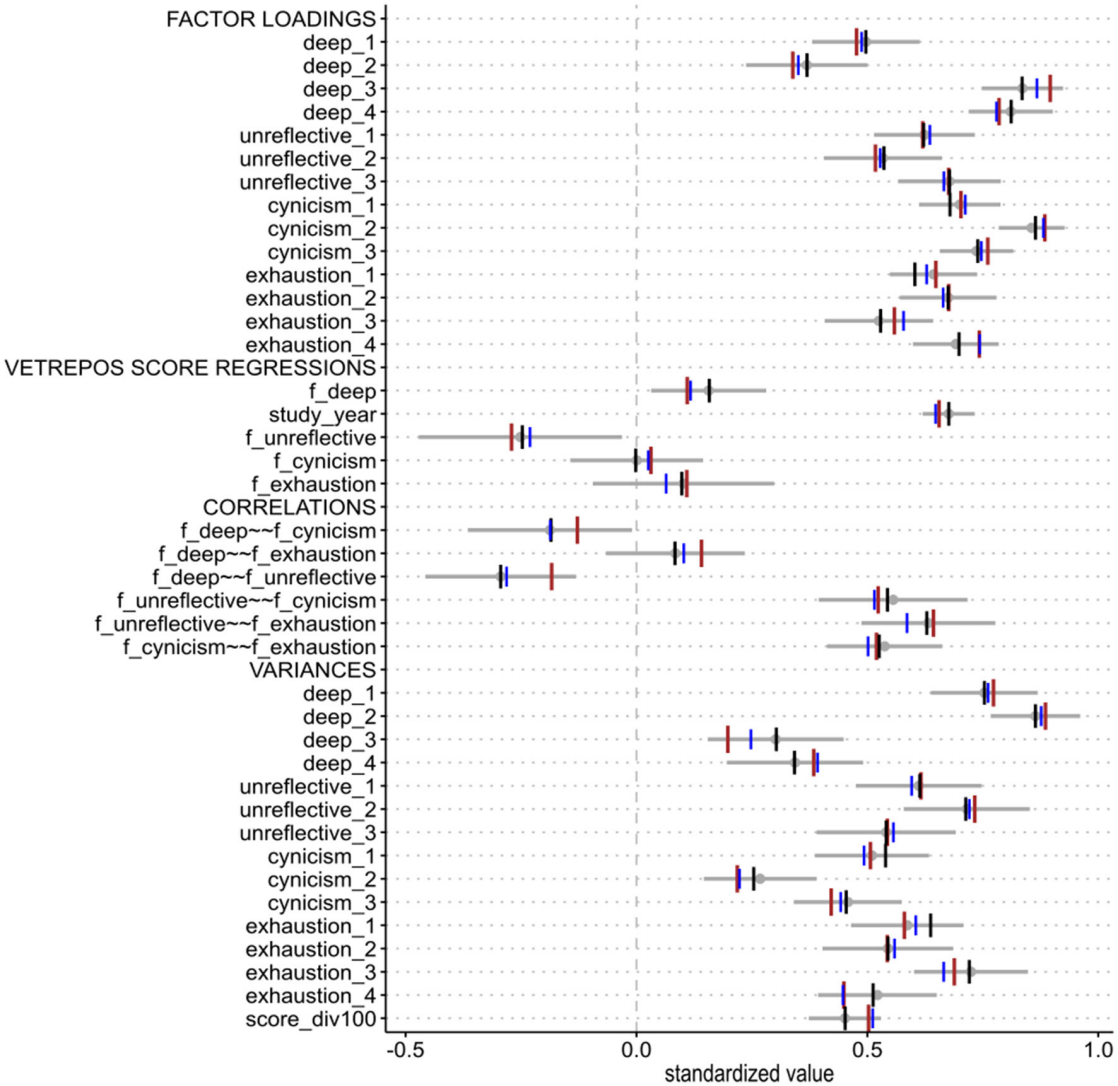
Standardized parameter estimates based on down-sampled (*N* = 173) or weighted data (*N* = 243) are compared with the estimates based on the unweighted complete data (*N* = 248). Blue = mean values of the estimates from down-sampled data (30 students from University F, 1,000 iterations). Brown = estimates from weighted data without University E. Gray = the complete data with a mean estimate and a 95% confidence interval. Black = estimates on the unweighted complete data with the covariance term between cynicism_1 and exhaustion_1 included in the model.

Next, we compared the values of the parameter estimates from the full SE model that were fitted to each of the datasets. As shown in Figure 7, all estimates fall within the 95% confidence interval determined by the unweighted complete data. The complete lists of the estimated values from measurement and full SE models are found in Supplementary Tables 4A, B, 7A, B, 8, 9.

### 3.7 Comparison of the latent traits and VetRepos test scores across study years

**Figure 8:**
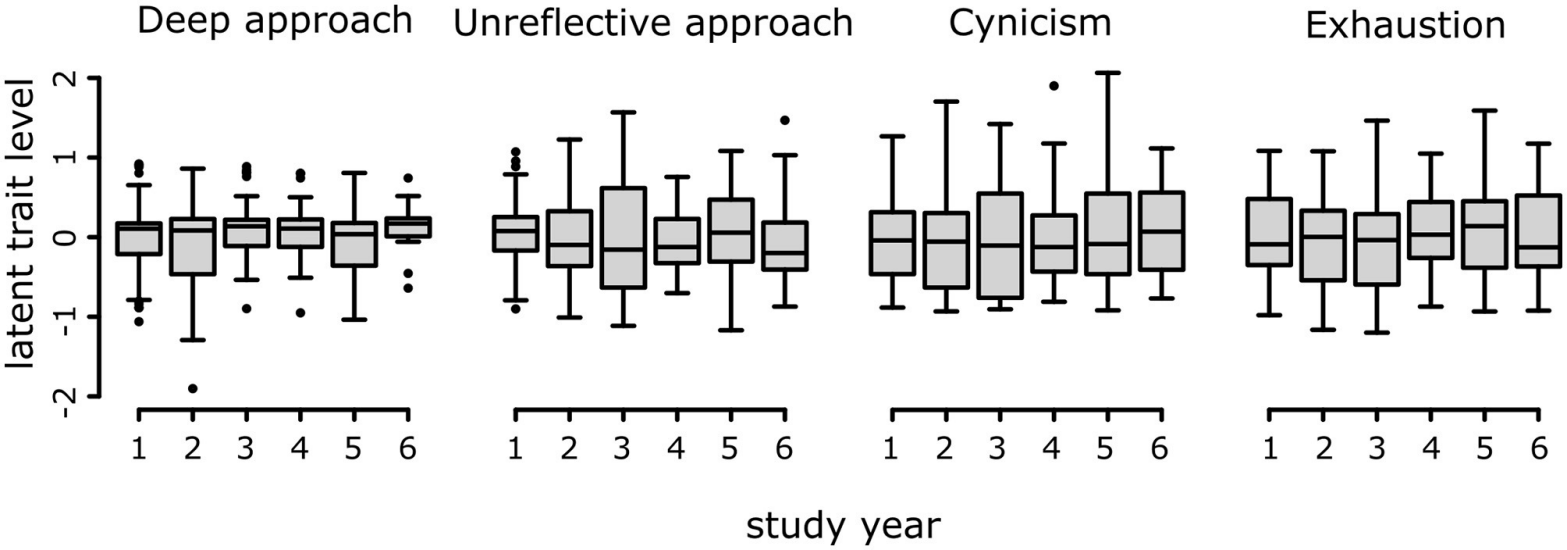
Latent factor scores across study years.

No differences were detected in the estimated latent factor scores (Figure 8). Instead, a significant and strong effect of the study year on VetRepos test scores was observed (Figure 2B). However, the difference in the scores was statistically non-significant from third to fifth year and between fifth- and sixth-year students (Kruskal test followed by Dunn's test for pairwise comparison of the means). Measurement non-invariance was found between the men and women, preventing direct comparisons between genders.

## 4 Discussion

This study reports the results of a cross-sectional structural equation modeling of the relationships of a single pan-curricular VetRepos test score with four latent traits: deep and unreflective approaches to learning, and the cynicism and exhaustion dimensions of self-perceived study burnout (Figure 5, Supplementary Table 7A). The modeling confirmed the regression of the VetRepos test score on the learning approaches. On the other hand, the observed association with self-perceived burnout was only indirect.

### 4.1 Scales, model fits and data structures

We carefully assessed the reliability of the scales and fit of the proposed model at different levels. The reliability coefficients (McDonald's ω_tot_ and Cronbach's α) suggest that the contribution of the true variance to the total variance in the responses to indicator items was acceptable (Table 3). The Person Separation Index for the VetRepos test database also suggests acceptable reliability for the knowledge measure used in this study (Table 3). Even though the full structural equation model failed the exact-fit test ($\chi_{\text{MLM}}^{2}$ = 148.20, df = 95, *p*-value = 0.00039), the estimated global fit indices (Table 5A) and the local fit assessed by inter-item correlation coefficient residuals and the standardized covariance residuals (Supplementary Tables 5A, 6A and Figure 6) support retaining the proposed theoretical model (43, 49). We acknowledge the unexpectedly large residual covariance between the indicators cynicism_1 and exhaustion_1. A modified model with a covariance term between these indicators fitted the data better than the original full SE model, as assessed by the χ^2^ difference test (Δχ^2^ = 17.68, df = 1, *p*-value = 0.0014) and by the drop in information criteria (ΔAIC = 15.7, ΔBIC = 12.2). As we cannot theoretically justify the covariance term and saw no improvement in the model parameter estimates (Supplementary Figure 1), we decided to retain the original model.

The students from University F accounted for 42.34% of the observations, and they were more junior (numerous 1st and 2nd year students) than students from the other universities. The inability to control the test settings, the students' preparation before the test, or their use of additional materials during the test could introduce bias into the data. Further, differences in language or cultural conventions between student groups that the model does not account for might influence the students' responses and, therefore, undermine the usefulness of the model. To mitigate the potential influence of the uneven distribution of the students between universities, we repeated the analyses using down-sampled and weighted datasets. The results from all of the control analyses conformed with the original results from the unweighted complete set of observations.

**Figure 9:**
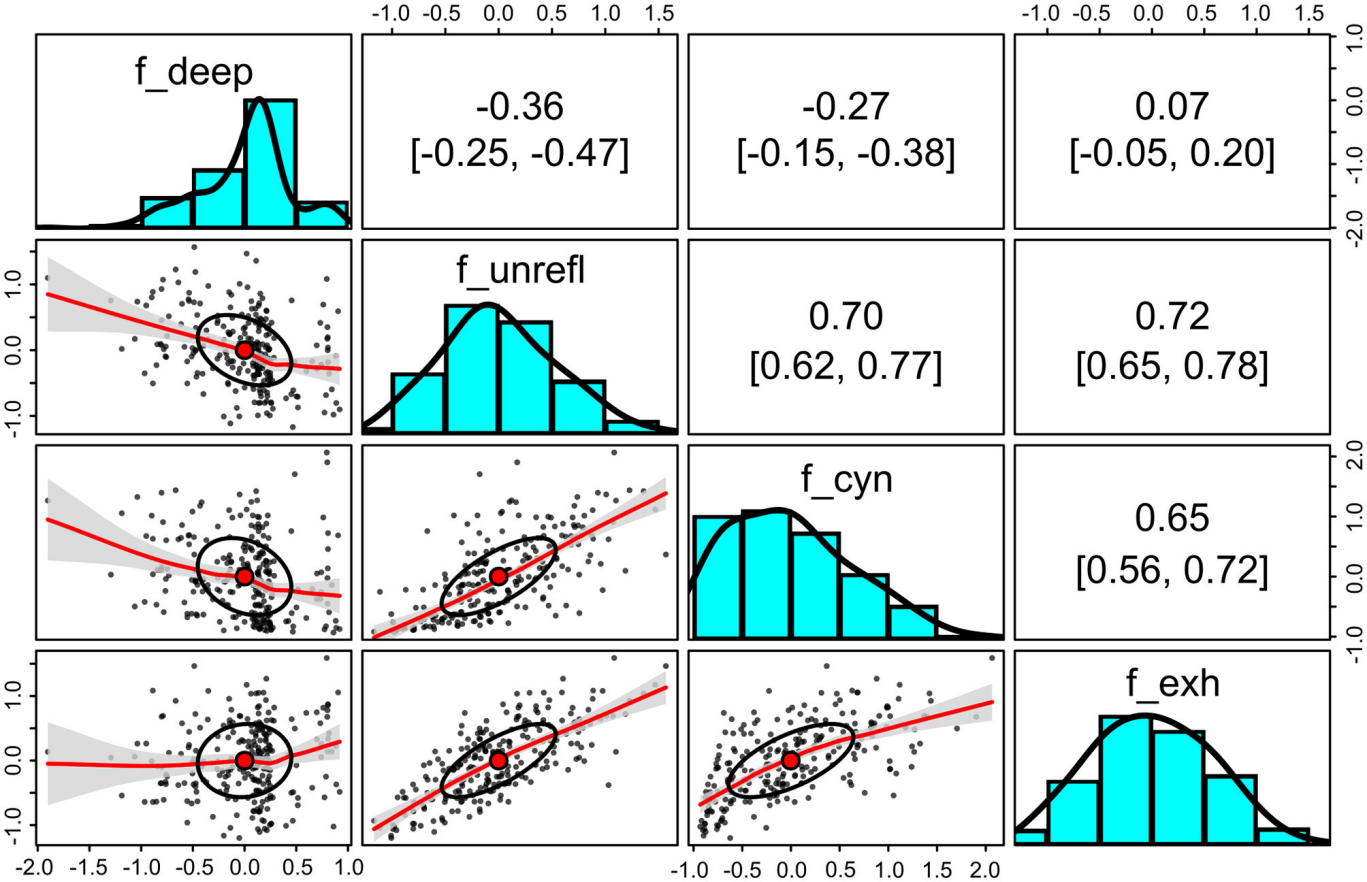
Estimated standardized levels of the deep and unreflective approaches to learning and for the self-perceived study burnout for each participant. Upper triangle: bivariate Spearman correlation coefficients with bootstrapped 95% confidence intervals (2,000 iterations). Lower triangle: bivariate scatter plots with correlation ellipses and loess fit with confidence intervals (α = 0.05). Diagonal: histograms with density plots. The raw scores are on the *x*-axis and the counts on the *y*-axis. *N* = 248.

One of the advantages of structural equational modeling over alternative methods, such as factorial analysis of variance, is that structural equation modeling (SEM) can estimate the attenuated (error-free) values of latent traits. Even though SEM cannot determine the true levels of latent traits, it effectively separates measurement errors from the observed values. This separation results in disattenuated correlation coefficients, which are typically larger between trait level estimates than between raw scores calculated from indicator items. For example, the disattenuated correlation coefficient between the estimated latent levels of the unreflective approach and cynicism was *r* = 0.70 (0.62–0.77; Figure 9). In contrast, the correlation coefficient between the raw sum scores of corresponding indicator items was only 0.42 (0.30–0.53; Figure 4).

The score of the VetRepos test, which spans the entire core of the veterinary curriculum, regresses positively on the study year (Figures 5 and 2B). The squared value of the standardized regression suggests that the study year explains ~46% of the variation in the VetRepos test score. A similar approach to square the regression coefficients of the full structural equation model suggests that the combined effect size of the learning approaches explains only ~9% of the total variation of the VetRepos test score. The remaining 45% of the variation is not accounted for by the model and probably includes differences between individuals, universities, and genders, as well as the error variance that could not be captured.

### 4.2 Burnout perceived by veterinary students

We did not estimate the prevalence of study burnout as the SBI-9 instrument is not designed for this purpose (17). However, other studies have reported burnout prevalence levels >40% among students in medicine and veterinary medicine (19, 20). Learning environments, including perceived low levels of support from staff and peers, cynical residents or interns, and clinical rotations with overnight calls, were associated with study burnout among medical students (50). Interestingly, a comparison of veterinary students with or without burnout revealed no differences between genders (19, 20), the stages of study, or study success regarding exam grades and passed exams (20). In contrast, burnout was associated with the perceived stress related to exams and various social contexts like colloquiums, contacts with teachers or pet owners, and fieldwork (20).

We, too, did not find an association between burnout and study success. The model-estimated regressions of the VetRepos test score on the latent traits of perceived burnout were non-significant (Figure 5). However, cynicism and exhaustion correlated significantly (α = 0.05) with the unreflective approach with very large correlation coefficients [*r* = 0.56 (95% ci = 0.40–0.72) vs. 0.63 (0.49–0.78), Supplementary Table 7A] (44). This suggests that the relationship between perceived burnout and the VetRepos test score is indirect and possibly mediated by the association with the unreflective approach (Figure 5). The nature of this relationship should be further investigated. Understanding the causality's direction and nuances would help design interventions for improving the students' wellbeing and learning. For example, to distance themselves from the unreflective approach, students might need help seeing how different courses and contents are linked. This could be done at various levels, including curriculum and course development. Increasing teacher interaction to support them in understanding the cumulative knowledge building in the curriculum could also benefit the students. In addition to improving the learning environment, the interventions could also be targeted to enhance the students' psychological flexibility (51, 52), which in working life has been shown to mitigate burnout (53, 54).

### 4.3 Comparison between genders

Unfortunately, we could not reject non-invariance between genders, which prevented the rigorous comparison of the factor scores between men and women. As there might be gender differences in students' learning approaches and in how they perceive study burnout, we carried out a superficial descriptive analysis of the responses to the study questionnaire in male and female students (Supplementary Figure 2, Supplementary Table 10). The female students responded by perceiving more exhaustion than the male students (Krustal.test χ^2^ = 14.80, df = 1, false discovery rate controlled *p*-value = 0.00012). No other differences were detected (α = 0.05).

The failure to show measurement invariance may have been due to the small size of our dataset, particularly the small number of men. However, the gender distribution of veterinary students in the current study reflects the international gender distribution in veterinary universities in general, and an increase in student numbers filling out the questionnaire would not have changed the ratio between female and male participants. On the other hand, there might be genuine gender-dependent differences that the model cannot account for. There is a need for additional studies to clarify these issues.

### 4.4 Conclusions

Our small pilot investigation underlines the potential of cooperation between European veterinary educational establishments for developing and improving veterinary education and supporting the students' wellbeing. We have demonstrated in this cross-sectional study that a single VetRepos test score can be successfully used in multinational settings to investigate the relationships between study success, learning approaches, and self-perceived burnout. Longitudinal and person-oriented investigations should complement our observations. Emphasis should be placed on the relationships between self-perceived burnout and learning approaches, including potential gender differences in these traits.

## Data availability statement

The dataset used in this study has been anonymized and deposited to the Finnish Social Science Data Archive from where it is available for research, teaching and study by the accession number FSD3873 (https://urn.fi/urn:nbn:fi:fsd:T-FSD3873). To ensure the anonymity of the participants information on the gender and home university has been removed.

## Ethics statement

The study was approved by the data protection officers of the participating universities and by the University of Helsinki Ethical Review Board in Humanities and Social and Behavioral Sciences (Statement 16/2022) according to the guidelines of the Finnish National Board on Research Integrity (55). The studies were conducted in accordance with the local legislation and institutional requirements. The participants provided their written informed consent to participate in this study.

## Author contributions

AI: Conceptualization, Data curation, Formal analysis, Funding acquisition, Investigation, Methodology, Project administration, Writing – original draft, Writing – review & editing. CC: Writing – review & editing, Formal analysis, Supervision. JW: Data curation, Formal analysis, Methodology, Supervision, Validation, Writing – review & editing. AP: Writing – review & editing, Supervision. AN: Writing – review & editing. RK-T: Funding acquisition, Writing – review & editing. AT: Writing – review & editing. ES: Funding acquisition, Writing – review & editing, Project administration. TH: Funding acquisition, Project administration, Writing – review & editing. TP: Writing – review & editing. CP: Writing – review & editing. PH: Funding acquisition, Project administration, Writing – review & editing.

## Group Members of VetRepos consortium

Marlies Beek, Harold G. J. Bok, Nina Dam Otten, Ann Kristin Egeli, Theo van Haeften, Peter Holm, Antti Iivanainen, Riikka Keto-Timonen, Hans Petter Kjæstad, Pierre Lekeux, Adam Dunstan Martin, Johanna Penell, Tina Holberg Pihl, Charles McLean Press, Andrea Tipold, Elisabeth Schaper, Karin Vargmar, Dax J. C. Vendrig, Jakob Wandall, Karin Elisabeth Zimmer.
