# Supplementary material for: Knowledge attainment, learning approaches, and self-perceived study burnout among European veterinary students
Source: Front Vet Sci. 2024 Jul 18;11:1292750. doi: 10.3389/fvets.2024.1292750 (PMC11291358; doi:10.3389/fvets.2024.1292750)
Supplement: Supplementary file 1 [file Data_Sheet_1.pdf]

Supplementary material

Supplementary Figures

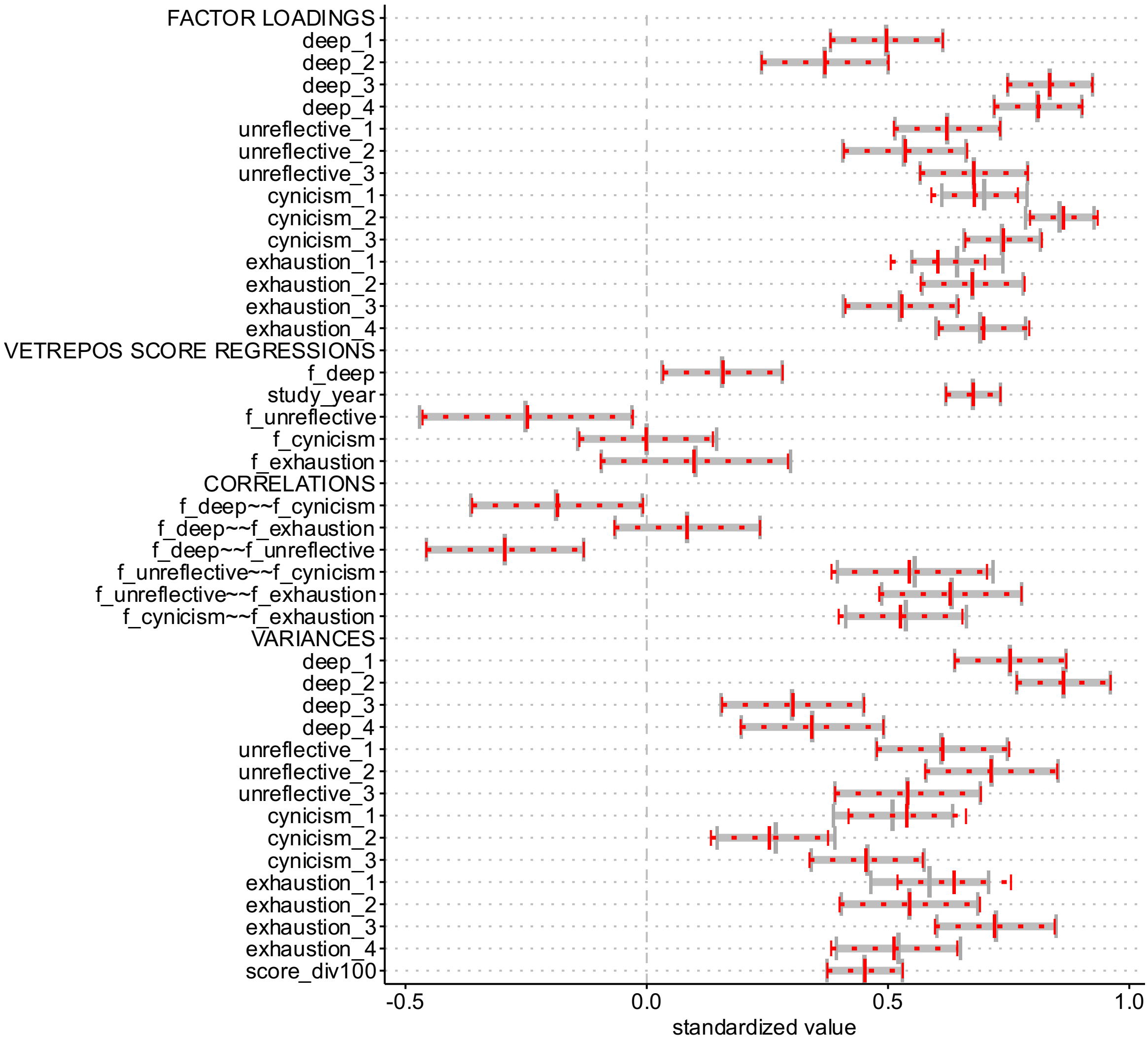

Supplementary Figure 1. Standardized parameter estimates on full SE model (gray) or full SE model with a covariance term between cynicism_1 and exhaustion_1 (red) fitted to the unweighted complete data (N=248). The estimates and the lower and upper bounds of a 95% confidence intervals are marked with vertical bars. The estimated standardized value of the introduced covariance term (correlation coefficient) between cynicism_1 and exhaustion_1 was 0.31 [95% confidence interval = 0.18–0.43].

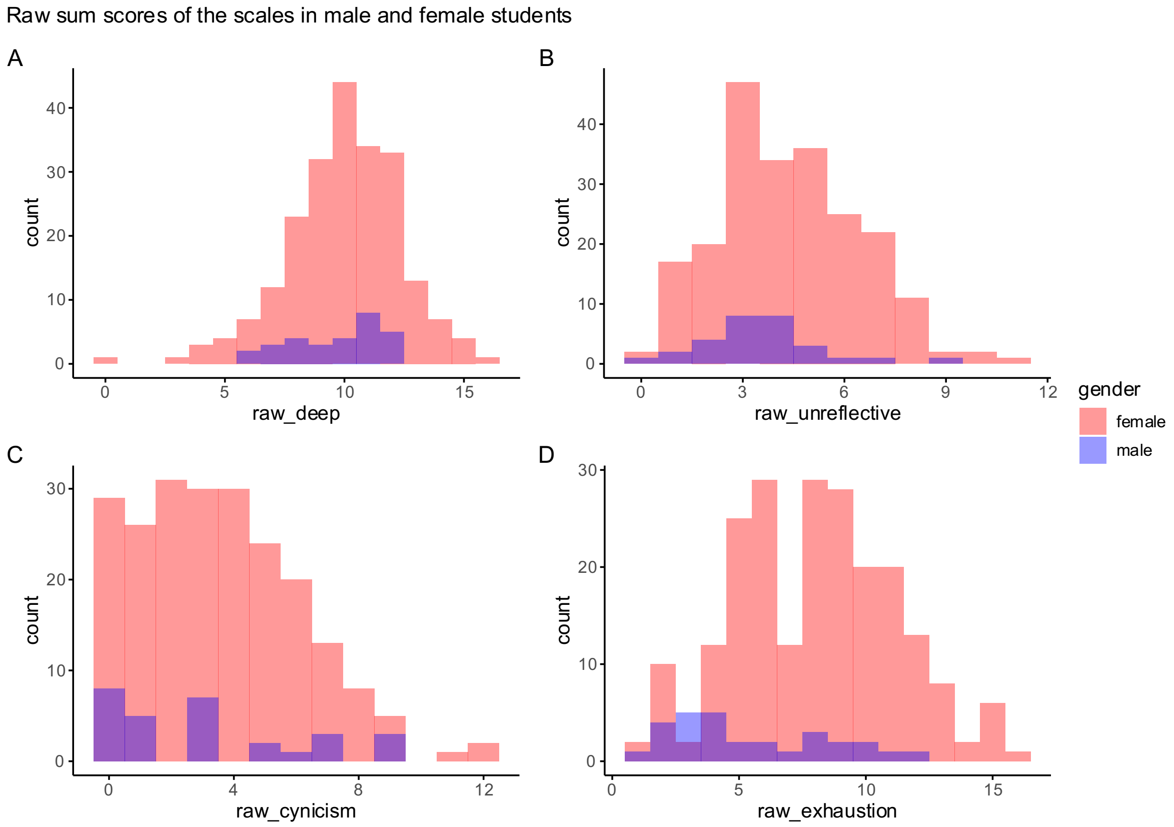

Supplementary Figure 2. Differential responses between genders to the indicator items. X-axis = the raw sum score of the responses to the indicator items for a single scale. Y = observation count. N = 246. (A–C) No differences were detected in responses to indicators of deep and unreflective approaches to learning, or self-perceived cynicism. (D) A differential response between genders was established to the items measuring self-perceived exhaustion ($\alpha = 0.05$). Kruskal.test $\chi^2 = 14.80$, df = 1, p-value = 0.00012. False discovery rate controlled p-value 0.00048.

Supplementary Tables 1–10

Supplementary Table 1. The indicator items in the study questionnaire.

	dimension	item_id	statement
Self-perceived study burnout	**cynicism**	cynicism_1	I feel a lack of study motivation and often think of giving up.
		cynicism_2	I feel that I am losing interest in my studies.
		cynicism_3	I'm continually wondering whether my studies have any meaning.
	**exhaustion**	exhaustion_1	I feel overwhelmed by the work related to my studies.
		exhaustion_2	I often sleep badly because of matters related to my studies.
		exhaustion_3	I brood over matters related to my studies during my free time.
		exhaustion_4	The pressure of my studies causes me problems in my close relationships with others.
	**inadequacy**	inadequacy_1	I often have feelings of inadequacy in my studies.
		inadequacy_2	I used to have higher expectations of my studies than I do now.
Learning approach	**unreflective**	unreflective_1	I often have trouble making sense of the things I have to learn.
		unreflective_2	Much of what I've learned seems no more than unrelated bits and pieces.
		unreflective_3	I am unable to understand the topics I need to learn because they are so complicated.
		unreflective_4	Often I have to repeat things in order to learn them.
	**deep**	deep_1	Ideas and perspectives I've come across while I'm studying make me contemplate them from all sides.
		deep_2	I look at evidence carefully to reach my own conclusion about what I'm studying.
		deep_3	I try to relate new material to my previous knowledge.
		deep_4	I try to relate what I have learned in one course to what I learn in other courses.

Supplementary Table 2A. Bivariate Spearman correlation coefficients of the indicator items for deep and unreflective approaches to learning and for self-perceived study burnout instrument SBI-9. B) Ninety-five per cent confidence intervals of the bivariate Spearman correlation coefficients of the indicators. Sum scores of the items for the various dimensions are also indicated. raw_burnout = sum score of the items in SBI-9.

	deep_1	deep_2	deep_3	deep_4	raw_deep	unreflective_1	unreflective_2	unreflective_3	unreflective_4	raw_unreflective4	cynicism_1	cynicism_2	cynicism_3	raw_cynicism	exhaustion_1	exhaustion_2	exhaustion_3	exhaustion_4	raw_exhaustion	inadequacy_1	inadequacy_2	raw_inadequacy	raw_burnout
deep_1	1.00	0.28	0.41	0.36	0.70	−0.08	−0.09	−0.07	0.09	−0.05	−0.07	−0.14	−0.04	−0.11	−0.05	0.00	0.08	0.00	0.02	−0.05	−0.06	−0.07	−0.06
deep_2	0.28	1.00	0.24	0.30	0.66	−0.04	0.01	−0.17	0.06	−0.03	−0.07	−0.08	−0.04	−0.08	−0.01	0.06	0.05	0.03	0.07	−0.04	−0.09	−0.07	−0.03
deep_3	0.41	0.24	1.00	0.68	0.75	−0.20	−0.15	−0.13	0.18	−0.12	−0.06	−0.15	−0.11	−0.13	0.04	−0.03	0.17	0.06	0.09	0.09	−0.02	0.03	0.00
deep_4	0.36	0.30	0.68	1.00	0.76	−0.15	−0.16	−0.11	0.11	−0.11	−0.09	−0.18	−0.10	−0.15	0.07	−0.03	0.16	0.07	0.10	0.08	0.06	0.07	0.01
raw_deep	0.70	0.66	0.75	0.76	1.00	−0.15	−0.12	−0.18	0.12	−0.12	−0.10	−0.21	−0.11	−0.18	−0.00	0.01	0.14	0.05	0.08	0.02	−0.05	−0.02	−0.03
unreflective_1	−0.08	−0.04	−0.20	−0.15	−0.15	1.00	0.35	0.41	0.25	0.78	0.27	0.21	0.20	0.26	0.34	0.23	0.09	0.27	0.32	0.31	0.19	0.30	0.36
unreflective_2	−0.09	0.01	−0.15	−0.16	−0.12	0.35	1.00	0.33	0.15	0.69	0.24	0.25	0.31	0.31	0.19	0.14	0.06	0.24	0.21	0.17	0.22	0.24	0.31
unreflective_3	−0.07	−0.17	−0.13	−0.11	−0.18	0.41	0.33	1.00	0.09	0.65	0.34	0.40	0.34	0.43	0.37	0.34	0.10	0.28	0.36	0.31	0.15	0.27	0.43
unreflective_4	0.09	0.06	0.18	0.11	0.12	0.25	0.15	0.09	1.00	0.52	0.10	−0.03	−0.09	−0.00	0.40	0.19	0.27	0.15	0.35	0.25	0.04	0.18	0.23
raw_unreflective4	−0.05	−0.03	−0.12	−0.11	−0.12	0.78	0.69	0.65	0.52	1.00	0.33	0.29	0.26	0.35	0.46	0.31	0.18	0.35	0.44	0.36	0.24	0.36	0.48
cynicism_1	−0.07	−0.07	−0.06	−0.09	−0.10	0.27	0.24	0.34	0.10	0.33	1.00	0.56	0.51	0.82	0.47	0.34	0.22	0.32	0.45	0.44	0.37	0.49	0.72
cynicism_2	−0.14	−0.08	−0.15	−0.18	−0.21	0.21	0.25	0.40	−0.03	0.29	0.56	1.00	0.67	0.86	0.31	0.33	0.20	0.30	0.38	0.33	0.44	0.46	0.70
cynicism_3	−0.04	−0.04	−0.11	−0.10	−0.11	0.20	0.31	0.34	−0.09	0.26	0.51	0.67	1.00	0.85	0.21	0.27	0.07	0.20	0.26	0.35	0.41	0.45	0.62
raw_cynicism	−0.11	−0.08	−0.13	−0.15	−0.18	0.26	0.31	0.43	−0.00	0.35	0.82	0.86	0.85	1.00	0.40	0.37	0.19	0.33	0.43	0.45	0.48	0.55	0.80
exhaustion_1	−0.05	−0.01	0.04	0.07	−0.00	0.34	0.19	0.37	0.40	0.46	0.47	0.31	0.21	0.40	1.00	0.41	0.30	0.39	0.69	0.57	0.33	0.54	0.67
exhaustion_2	0.00	0.06	−0.03	−0.03	0.01	0.23	0.14	0.34	0.19	0.31	0.34	0.33	0.27	0.37	0.41	1.00	0.37	0.50	0.79	0.35	0.14	0.29	0.64
exhaustion_3	0.08	0.05	0.17	0.16	0.14	0.09	0.06	0.10	0.27	0.18	0.22	0.20	0.07	0.19	0.30	0.37	1.00	0.41	0.67	0.33	0.24	0.34	0.52
exhaustion_4	0.00	0.03	0.06	0.07	0.05	0.27	0.24	0.28	0.15	0.35	0.32	0.30	0.20	0.33	0.39	0.50	0.41	1.00	0.78	0.29	0.28	0.34	0.63
raw_exhaustion	0.02	0.07	0.09	0.10	0.08	0.32	0.21	0.36	0.35	0.44	0.45	0.38	0.26	0.43	0.69	0.79	0.67	0.78	1.00	0.51	0.32	0.50	0.83
inadequacy_1	−0.05	−0.04	0.09	0.08	0.02	0.31	0.17	0.31	0.25	0.36	0.44	0.33	0.35	0.45	0.57	0.35	0.33	0.29	0.51	1.00	0.39	0.82	0.68
inadequacy_2	−0.06	−0.09	−0.02	0.06	−0.05	0.19	0.22	0.15	0.04	0.24	0.37	0.44	0.41	0.48	0.33	0.14	0.24	0.28	0.32	0.39	1.00	0.84	0.61
raw_inadequacy	−0.07	−0.07	0.03	0.07	−0.02	0.30	0.24	0.27	0.18	0.36	0.49	0.46	0.45	0.55	0.54	0.29	0.34	0.34	0.50	0.82	0.84	1.00	0.78
raw_burnout	−0.06	−0.03	0.00	0.01	−0.03	0.36	0.31	0.43	0.23	0.48	0.72	0.70	0.62	0.80	0.67	0.64	0.52	0.63	0.83	0.68	0.61	0.78	1.00

Supplementary Table 2B. Ninety-five per cent confidence intervals of the bivariate Spearman correlation coefficients of the indicators. Sum scores of the items for the various dimensions are also indicated. raw_burnout = sum score of the items in SBI-9.

	deep_1	deep_2	deep_3	deep_4	raw_deep	unreflective_1	unreflective_2	unreflective_3	unreflective_4	raw_unreflective4	cynicism_1	cynicism_2	cynicism_3	raw_cynicism	exhaustion_1	exhaustion_2	exhaustion_3	exhaustion_4	raw_exhaustion	inadequacy_1	inadequacy_2	raw_inadequacy	raw_burnout
deep_1	1.00	0.40	0.51	0.46	0.76	−0.20	−0.21	−0.20	0.21	−0.18	−0.19	−0.26	−0.17	−0.24	−0.18	0.13	0.22	0.13	0.15	−0.17	−0.20	−0.20	−0.19
deep_2	0.16	1.00	0.36	0.41	0.73	−0.17	0.14	−0.29	0.18	−0.16	−0.20	−0.21	−0.16	−0.20	−0.14	0.18	0.18	0.15	0.19	−0.16	−0.21	−0.19	−0.14
deep_3	0.30	0.12	1.00	0.76	0.81	−0.32	−0.26	−0.25	0.31	−0.23	−0.18	−0.28	−0.25	−0.26	0.16	−0.16	0.30	0.18	0.22	0.22	−0.15	0.17	0.13
deep_4	0.25	0.18	0.59	1.00	0.81	−0.28	−0.28	−0.24	0.23	−0.24	−0.21	−0.31	−0.23	−0.28	0.19	−0.15	0.29	0.19	0.21	0.21	0.19	0.20	0.14
raw_deep	0.62	0.57	0.69	0.69	1.00	−0.27	−0.24	−0.30	0.24	−0.24	−0.22	−0.33	−0.24	−0.30	−0.13	0.13	0.27	0.17	0.21	0.15	−0.18	−0.15	−0.16
unreflective_1	0.04	0.09	−0.08	−0.02	−0.03	1.00	0.46	0.52	0.36	0.83	0.38	0.32	0.32	0.37	0.45	0.35	0.22	0.38	0.43	0.42	0.31	0.41	0.46
unreflective_2	0.04	−0.12	−0.03	−0.04	0.01	0.23	1.00	0.44	0.26	0.75	0.37	0.37	0.42	0.43	0.32	0.26	0.18	0.35	0.34	0.30	0.33	0.36	0.43
unreflective_3	0.06	−0.04	0.00	0.02	−0.05	0.29	0.21	1.00	0.21	0.72	0.45	0.50	0.45	0.53	0.47	0.45	0.23	0.39	0.47	0.42	0.28	0.38	0.53
unreflective_4	−0.03	−0.07	0.04	−0.02	−0.01	0.15	0.04	−0.04	1.00	0.60	0.22	−0.15	−0.21	−0.13	0.50	0.30	0.39	0.27	0.45	0.36	0.16	0.29	0.34
raw_unreflective4	0.07	0.10	0.01	0.01	0.02	0.72	0.61	0.55	0.43	1.00	0.44	0.41	0.38	0.46	0.56	0.41	0.29	0.44	0.54	0.47	0.35	0.47	0.57
cynicism_1	0.06	0.05	0.07	0.04	0.03	0.15	0.11	0.22	−0.02	0.22	1.00	0.65	0.59	0.87	0.56	0.46	0.34	0.43	0.56	0.55	0.47	0.58	0.78
cynicism_2	−0.01	0.05	−0.03	−0.05	−0.08	0.09	0.12	0.28	0.10	0.16	0.46	1.00	0.75	0.90	0.41	0.45	0.32	0.41	0.48	0.44	0.54	0.56	0.76
cynicism_3	0.08	0.09	0.02	0.04	0.02	0.07	0.19	0.22	0.03	0.14	0.41	0.59	1.00	0.88	0.33	0.38	0.19	0.33	0.37	0.46	0.51	0.55	0.69
raw_cynicism	0.02	0.05	−0.01	−0.02	−0.05	0.14	0.18	0.31	0.12	0.22	0.77	0.81	0.81	1.00	0.50	0.48	0.31	0.44	0.54	0.55	0.57	0.64	0.84
exhaustion_1	0.08	0.12	−0.08	−0.06	0.12	0.23	0.06	0.25	0.28	0.36	0.37	0.19	0.09	0.29	1.00	0.51	0.42	0.50	0.75	0.65	0.44	0.63	0.74
exhaustion_2	−0.13	−0.06	0.10	0.09	−0.12	0.10	0.01	0.22	0.06	0.18	0.22	0.21	0.15	0.25	0.31	1.00	0.48	0.60	0.84	0.46	0.26	0.40	0.71
exhaustion_3	−0.05	−0.08	0.03	0.03	0.01	−0.03	−0.07	−0.03	0.13	0.06	0.09	0.07	−0.06	0.06	0.18	0.25	1.00	0.52	0.74	0.44	0.36	0.45	0.61
exhaustion_4	−0.12	−0.09	−0.07	−0.06	−0.08	0.15	0.11	0.16	0.03	0.23	0.19	0.18	0.07	0.21	0.27	0.39	0.30	1.00	0.83	0.41	0.39	0.46	0.71
raw_exhaustion	−0.11	−0.06	−0.03	−0.02	−0.04	0.20	0.08	0.25	0.23	0.33	0.34	0.27	0.13	0.32	0.61	0.74	0.59	0.72	1.00	0.61	0.44	0.60	0.87
inadequacy_1	0.08	0.09	−0.05	−0.05	−0.11	0.19	0.05	0.18	0.13	0.25	0.33	0.22	0.24	0.33	0.48	0.23	0.20	0.17	0.40	1.00	0.50	0.86	0.75
inadequacy_2	0.07	0.04	0.11	−0.07	0.09	0.06	0.09	0.02	−0.08	0.11	0.25	0.33	0.30	0.37	0.22	0.01	0.12	0.15	0.20	0.26	1.00	0.87	0.69
raw_inadequacy	0.06	0.06	−0.10	−0.06	0.11	0.18	0.11	0.15	0.05	0.24	0.38	0.36	0.34	0.46	0.45	0.17	0.22	0.22	0.39	0.77	0.80	1.00	0.83
raw_burnout	0.07	0.10	−0.12	−0.11	0.09	0.25	0.18	0.33	0.11	0.37	0.64	0.63	0.54	0.76	0.59	0.55	0.42	0.55	0.78	0.60	0.52	0.71	1.00

Supplementary Table 3. (A) Skewness and curtosis of the VetRepos Test score distribution. (B) Multivariate Mardia test on the various scales used in the study. Gray color indicates that the null hypothesis was rejected (α = 0.05, two-tailed alternative hypothesis).

A.

	lower_ci	estimate	upper_ci	pvalue
skewness	-0.15	0.03	0.20	0.75
kurtosis	-0.98	-0.75	-0.48	< 0.001

B.

	kurtosis			skewness			
	b2d	z	p2	b1d	chi	df	p1
all_scales	344.92	6.79	0.00	36.00	1488.08	969	1.18E-24
deep_approach	27.28	3.73	0.00	2.15	89.07	20	1.08E-10
unreflective_approach4	24.14	0.15	0.88	1.80	74.54	20	3.25E-08
unreflective_approach3	14.25	-1.08	0.28	0.71	29.51	10	1.03E-03
cynicism	16.30	1.87	0.06	2.01	83.08	10	1.25E-13
exhaustion	22.82	-1.34	0.18	1.15	47.53	20	4.95E-04
inadequacy	6.77	-2.43	0.02	0.03	1.32	4	8.58E-01

Supplementary Table 4A. Standardized coefficients of the fitted measurement model on the complete unweighted data (N=248). lhs: left-hand side variable, rhs: right-hand side variable, op: operator, est.std: standardized estimate, se: standard error, z: z statistics, ci.lower: lower bound and ci.upper: upper bound of the 95% confidence interval of the standardized estimate, h2: communality, =~: factor loadings, ~~: covariation, NA: not available. Non-significant estimates are colored gray.

lhs	op	rhs	est.std	se	z	pvalue	ci.lower	ci.upper	h2
f_deep	=~	deep_1	0.50	0.06	8.40	0.0E+00	0.38	0.61	0.25
f_deep	=~	deep_2	0.38	0.07	5.63	1.8E-08	0.25	0.51	0.14
f_deep	=~	deep_3	0.82	0.05	17.03	0.0E+00	0.73	0.92	0.68
f_deep	=~	deep_4	0.82	0.05	17.07	0.0E+00	0.73	0.92	0.68
f_unreflective	=~	unreflective_1	0.63	0.06	11.10	0.0E+00	0.52	0.74	0.40
f_unreflective	=~	unreflective_2	0.53	0.07	8.00	1.3E-15	0.40	0.66	0.28
f_unreflective	=~	unreflective_3	0.68	0.06	11.67	0.0E+00	0.56	0.79	0.46
f_cynicism	=~	cynicism_1	0.70	0.05	15.54	0.0E+00	0.61	0.79	0.49
f_cynicism	=~	cynicism_2	0.86	0.04	23.65	0.0E+00	0.79	0.93	0.74
f_cynicism	=~	cynicism_3	0.73	0.04	18.10	0.0E+00	0.65	0.81	0.54
f_exhaustion	=~	exhaustion_1	0.64	0.05	13.26	0.0E+00	0.55	0.74	0.41
f_exhaustion	=~	exhaustion_2	0.68	0.05	12.69	0.0E+00	0.57	0.78	0.46
f_exhaustion	=~	exhaustion_3	0.52	0.06	8.76	0.0E+00	0.41	0.64	0.28
f_exhaustion	=~	exhaustion_4	0.69	0.05	14.68	0.0E+00	0.60	0.78	0.48
deep_1	~~	deep_1	0.75	0.06	12.76	0.0E+00	0.64	0.87	NA
deep_2	~~	deep_2	0.86	0.05	17.02	0.0E+00	0.76	0.96	NA
deep_3	~~	deep_3	0.32	0.08	4.08	4.5E-05	0.17	0.48	NA
deep_4	~~	deep_4	0.32	0.08	4.11	4.0E-05	0.17	0.48	NA
unreflective_1	~~	unreflective_1	0.60	0.07	8.46	0.0E+00	0.46	0.74	NA
unreflective_2	~~	unreflective_2	0.72	0.07	10.25	0.0E+00	0.58	0.86	NA
unreflective_3	~~	unreflective_3	0.54	0.08	6.95	3.6E-12	0.39	0.70	NA
cynicism_1	~~	cynicism_1	0.51	0.06	8.11	4.4E-16	0.39	0.63	NA
cynicism_2	~~	cynicism_2	0.26	0.06	4.25	2.1E-05	0.14	0.39	NA
cynicism_3	~~	cynicism_3	0.46	0.06	7.72	1.1E-14	0.34	0.58	NA
exhaustion_1	~~	exhaustion_1	0.59	0.06	9.47	0.0E+00	0.47	0.71	NA
exhaustion_2	~~	exhaustion_2	0.54	0.07	7.54	4.8E-14	0.40	0.68	NA
exhaustion_3	~~	exhaustion_3	0.72	0.06	11.52	0.0E+00	0.60	0.85	NA
exhaustion_4	~~	exhaustion_4	0.52	0.07	7.98	1.6E-15	0.39	0.65	NA
f_deep	~~	f_deep	1.00	0.00	NA	NA	1.00	1.00	NA
f_unreflective	~~	f_unreflective	1.00	0.00	NA	NA	1.00	1.00	NA
f_cynicism	~~	f_cynicism	1.00	0.00	NA	NA	1.00	1.00	NA
f_exhaustion	~~	f_exhaustion	1.00	0.00	NA	NA	1.00	1.00	NA
f_deep	~~	f_unreflective	-0.29	0.08	-3.54	4.1E-04	-0.46	-0.13	NA
f_deep	~~	f_cynicism	-0.19	0.09	-2.07	3.8E-02	-0.37	-0.01	NA
f_deep	~~	f_exhaustion	0.08	0.08	1.09	2.8E-01	-0.07	0.23	NA
f_unreflective	~~	f_cynicism	0.55	0.08	6.73	1.7E-11	0.39	0.71	NA
f_unreflective	~~	f_exhaustion	0.63	0.07	8.53	0.0E+00	0.49	0.78	NA
f_cynicism	~~	f_exhaustion	0.54	0.06	8.42	0.0E+00	0.41	0.66	NA

Supplementary Table 4B. Standardized coefficients of the fitted measurement model on the weighted data (N=243). lhs: left-hand side variable, rhs: right-hand side variable, op: operator, est.std: standardized estimate, se: standard error, z: z statistics, ci.lower: lower bound and ci.upper: upper bound of the 95% confidence interval of the standardized estimate, h2: communality, =~: factor loadings, ~~: covariation, NA: not available. Non-significant estimates are colored gray.

lhs	op	rhs	est.std	se	z	pvalue	ci.lower	ci.upper	h2
f_deep	=~	deep_1	0.48	0.06	7.54	0.0E+00	0.35	0.60	0.23
f_deep	=~	deep_2	0.34	0.07	4.99	0.0E+00	0.21	0.48	0.12
f_deep	=~	deep_3	0.89	0.05	16.46	0.0E+00	0.78	0.99	0.79
f_deep	=~	deep_4	0.79	0.05	15.34	0.0E+00	0.69	0.89	0.63
f_unreflective	=~	unreflective_1	0.62	0.06	10.90	0.0E+00	0.51	0.73	0.39
f_unreflective	=~	unreflective_2	0.51	0.07	7.67	0.0E+00	0.38	0.64	0.26
f_unreflective	=~	unreflective_3	0.68	0.06	11.28	0.0E+00	0.56	0.79	0.46
f_cynicism	=~	cynicism_1	0.70	0.04	15.87	0.0E+00	0.62	0.79	0.49
f_cynicism	=~	cynicism_2	0.88	0.04	24.13	0.0E+00	0.81	0.96	0.78
f_cynicism	=~	cynicism_3	0.76	0.04	18.66	0.0E+00	0.68	0.84	0.58
f_exhaustion	=~	exhaustion_1	0.65	0.05	13.91	0.0E+00	0.56	0.74	0.42
f_exhaustion	=~	exhaustion_2	0.68	0.05	13.54	0.0E+00	0.58	0.77	0.46
f_exhaustion	=~	exhaustion_3	0.56	0.06	10.13	0.0E+00	0.45	0.67	0.31
f_exhaustion	=~	exhaustion_4	0.74	0.04	16.63	0.0E+00	0.66	0.83	0.55
deep_1	~~	deep_1	0.77	0.06	12.72	0.0E+00	0.65	0.89	NA
deep_2	~~	deep_2	0.88	0.05	18.63	0.0E+00	0.79	0.97	NA
deep_3	~~	deep_3	0.21	0.10	2.21	3.0E-02	0.02	0.40	NA
deep_4	~~	deep_4	0.37	0.08	4.58	0.0E+00	0.21	0.53	NA
unreflective_1	~~	unreflective_1	0.61	0.07	8.60	0.0E+00	0.47	0.75	NA
unreflective_2	~~	unreflective_2	0.74	0.07	10.73	0.0E+00	0.60	0.87	NA
unreflective_3	~~	unreflective_3	0.54	0.08	6.69	0.0E+00	0.38	0.70	NA
cynicism_1	~~	cynicism_1	0.51	0.06	8.15	0.0E+00	0.38	0.63	NA
cynicism_2	~~	cynicism_2	0.22	0.06	3.37	0.0E+00	0.09	0.35	NA
cynicism_3	~~	cynicism_3	0.42	0.06	6.79	0.0E+00	0.30	0.54	NA
exhaustion_1	~~	exhaustion_1	0.58	0.06	9.66	0.0E+00	0.46	0.70	NA
exhaustion_2	~~	exhaustion_2	0.54	0.07	8.01	0.0E+00	0.41	0.67	NA
exhaustion_3	~~	exhaustion_3	0.69	0.06	11.19	0.0E+00	0.57	0.81	NA
exhaustion_4	~~	exhaustion_4	0.45	0.07	6.75	0.0E+00	0.32	0.58	NA
f_deep	~~	f_deep	1.00	0.00	NA	NA	1.00	1.00	NA
f_unreflective	~~	f_unreflective	1.00	0.00	NA	NA	1.00	1.00	NA
f_cynicism	~~	f_cynicism	1.00	0.00	NA	NA	1.00	1.00	NA
f_exhaustion	~~	f_exhaustion	1.00	0.00	NA	NA	1.00	1.00	NA
f_deep	~~	f_unreflective	-0.18	0.09	-2.11	3.0E-02	-0.35	-0.01	NA
f_deep	~~	f_cynicism	-0.13	0.09	-1.45	1.5E-01	-0.30	0.05	NA
f_deep	~~	f_exhaustion	0.14	0.07	1.93	5.0E-02	0.00	0.28	NA
f_unreflective	~~	f_cynicism	0.52	0.08	6.34	0.0E+00	0.36	0.68	NA
f_unreflective	~~	f_exhaustion	0.64	0.07	9.07	0.0E+00	0.50	0.78	NA
f_cynicism	~~	f_exhaustion	0.52	0.06	8.59	0.0E+00	0.40	0.64	NA

Supplementary Table 5. Interitem residual correlation coefficients in the fitted full structural equation model. All residuals are between -0.3 and 0.3. Indicator items: deep_1–4 for the deep approach, unreflective_1–3 for the unreflective approach, cynicism_1–3 for cynicism, exhaustion_1–4 for exhaustion. score_div100: VetRepos Test score divided by 100. The transformation does not affect modeling but brings the variances closer to each other. (A) Unweighted complete data (N=248). (B) Weighted data (N=243).

A.	deep_1	deep_2	deep_3	deep_4	unreflective_1	unreflective_2	unreflective_3	cynicism_1	cynicism_2	cynicism_3	exhaustion_1	exhaustion_2	exhaustion_3	exhaustion_4	score_div100
**deep_2**	1.5E-01														
**deep_3**	-2.9E-03	-4.4E-02													
**deep_4**	-1.8E-02	1.3E-02	4.5E-03												
**unreflective_1**	2.2E-03	2.7E-02	-4.2E-02	-1.6E-02											
**unreflective_2**	-3.5E-02	4.7E-02	-1.0E-02	-3.9E-02	4.2E-02										
**unreflective_3**	5.0E-02	-7.2E-02	3.2E-02	4.0E-02	-5.6E-03	-2.7E-02									
**cynicism_1**	-8.0E-03	-3.0E-02	5.5E-02	1.0E-02	2.8E-02	1.9E-02	6.1E-02								
**cynicism_2**	-5.8E-02	-6.2E-03	1.3E-02	-2.7E-02	-1.0E-01	4.4E-03	4.8E-02	-1.6E-02							
**cynicism_3**	9.4E-03	-1.4E-02	-1.1E-02	1.4E-02	-6.7E-02	8.0E-02	-9.5E-03	-1.9E-02	2.2E-02						
**exhaustion_1**	-1.0E-01	-4.4E-02	-2.8E-02	-1.1E-02	1.1E-01	2.5E-03	1.1E-01	2.3E-01	1.8E-02	-3.0E-02					
**exhaustion_2**	-3.2E-02	4.9E-02	-4.7E-02	-6.5E-02	-1.4E-02	-9.7E-02	3.7E-02	9.2E-02	-2.0E-02	-3.3E-02	-1.3E-02				
**exhaustion_3**	5.5E-02	3.3E-02	1.3E-01	1.1E-01	-9.5E-02	-1.2E-01	-9.8E-02	1.9E-02	-1.1E-02	-1.5E-01	-2.3E-02	2.3E-02			
**exhaustion_4**	-1.6E-02	1.7E-02	1.4E-02	8.6E-03	7.5E-03	1.3E-02	-1.2E-02	5.5E-02	-2.7E-02	-8.1E-02	-4.9E-02	2.4E-02	5.0E-02		
**score_div100**	4.9E-02	-7.1E-02	6.6E-02	5.2E-02	-3.4E-02	4.1E-05	-1.8E-02	2.2E-02	8.4E-02	-6.1E-02	9.5E-02	-7.0E-02	1.1E-01	-7.0E-03	
**study_year**	9.0E-02	-3.5E-02	6.9E-02	1.1E-01	-7.5E-02	3.3E-02	-3.5E-02	1.7E-02	8.0E-02	5.2E-03	1.2E-01	-5.7E-02	7.8E-02	4.4E-03	1.2E-02

B.	deep_1	deep_2	deep_3	deep_4	unreflective_1	unreflective_2	unreflective_3	cynicism_1	cynicism_2	cynicism_3	exhaustion_1	exhaustion_2	exhaustion_3	exhaustion_4	score_div100
**deep_2**	1.7E-01														
**deep_3**	-2.8E-03	-2.4E-02													
**deep_4**	-1.4E-02	1.6E-02	1.8E-03												
**unreflective_1**	-6.9E-02	-1.0E-02	-1.0E-01	-7.2E-02											
**unreflective_2**	-2.4E-02	8.4E-02	-6.0E-02	-3.6E-02	2.6E-02										
**unreflective_3**	7.4E-02	-3.5E-02	9.5E-02	1.3E-01	-8.6E-03	-8.9E-03									
**cynicism_1**	1.3E-02	-2.1E-02	2.2E-02	9.4E-03	3.4E-02	4.2E-02	9.0E-02								
**cynicism_2**	-2.6E-02	2.1E-02	8.1E-03	-2.7E-02	-1.0E-01	2.7E-02	4.9E-02	-1.6E-02							
**cynicism_3**	2.4E-02	-2.3E-02	-1.6E-02	1.6E-02	-9.2E-02	5.2E-02	-2.1E-02	-1.1E-02	1.6E-02						
**exhaustion_1**	-8.6E-02	-6.2E-02	-2.1E-02	-8.2E-03	1.0E-01	-1.9E-02	1.4E-01	2.6E-01	4.0E-02	-3.8E-02					
**exhaustion_2**	-9.4E-02	-2.9E-02	-6.6E-02	-6.9E-02	-9.7E-03	-1.3E-01	3.4E-02	7.6E-02	-5.5E-03	-1.9E-02	-9.0E-04				
**exhaustion_3**	-1.9E-02	-3.0E-02	4.7E-02	2.7E-02	-1.4E-01	-1.1E-01	-5.4E-02	3.2E-02	-1.4E-02	-1.3E-01	-3.1E-02	3.4E-02			
**exhaustion_4**	-2.2E-02	7.4E-03	6.0E-02	6.4E-02	7.9E-03	5.7E-03	-6.0E-03	4.0E-02	-3.6E-02	-7.2E-02	-5.3E-02	1.1E-02	5.2E-02		
**score_div100**	2.5E-02	-5.3E-02	-1.8E-02	-8.9E-03	-2.5E-02	1.3E-02	4.6E-02	6.8E-02	1.1E-01	-8.7E-03	9.8E-02	-3.2E-02	7.3E-02	1.1E-02	
**study_year**	4.1E-02	-3.8E-03	-4.6E-02	9.4E-03	-3.1E-02	5.9E-02	4.3E-02	8.3E-02	1.3E-01	9.0E-02	1.2E-01	-1.6E-03	5.8E-02	2.7E-02	-3.4E-03

Supplementary Table 6. Standardized interitem residual covariances in the fitted full structural equation model. Indicator items: deep_1–4 for the deep approach, unreflective_1–3 for the unreflective approach, cynicism_1–3 for cynicism, exhaustion_1–4 for exhaustion. score_div100: VetRepos Test score divided by 100. The transformation does not affect modeling but brings the variances closer to each other. (A) Complete unweighted data (N=248). The maximum residual is 4.86 between cynicism_1 and exhaustion_1. (B) Weighted data (N=243). The maximum residual is 5.41 between cynicism_1 and exhaustion_1.

A.	deep_1	deep_2	deep_3	deep_4	unreflective_1	unreflective_2	unreflective_3	cynicism_1	cynicism_2	cynicism_3	exhaustion_1	exhaustion_2	exhaustion_3	exhaustion_4	score_div100	study_year
**deep_1**	0.00															
**deep_2**	2.93	0.00														
**deep_3**	-0.17	-2.44	0.00													
**deep_4**	-0.77	0.52	1.14	0.00												
**unreflective_1**	0.04	0.45	-0.88	-0.31	0.00											
**unreflective_2**	-0.65	0.78	-0.25	-0.91	1.37	0.00										
**unreflective_3**	0.79	-1.24	0.68	0.85	-0.31	-1.07	0.00									
**cynicism_1**	-0.15	-0.49	1.17	0.22	0.66	0.41	1.50	0.00								
**cynicism_2**	-1.00	-0.11	0.41	-0.78	-2.92	0.12	1.28	-2.26	0.00							
**cynicism_3**	0.16	-0.23	-0.23	0.33	-1.57	1.87	-0.21	-1.24	3.19	0.00						
**exhaustion_1**	-1.71	-0.79	-0.61	-0.24	2.13	0.05	2.44	4.86	0.42	-0.68	0.00					
**exhaustion_2**	-0.51	0.81	-0.99	-1.35	-0.35	-2.23	0.89	2.01	-0.51	-0.76	-0.53	0.00				
**exhaustion_3**	0.84	0.53	2.12	1.87	-1.96	-2.23	-1.96	0.35	-0.23	-2.70	-0.74	0.73	0.00			
**exhaustion_4**	-0.28	0.31	0.33	0.20	0.17	0.29	-0.33	1.14	-0.77	-1.72	-2.14	1.19	1.54	0.00		
**score_div100**	0.96	-1.25	1.45	1.18	-0.74	-0.04	-0.40	0.41	1.83	-1.28	1.70	-1.41	1.96	-0.14	1.38	
**study_year**	1.48	-0.57	1.06	1.64	-1.17	0.55	-0.54	0.26	1.28	0.08	1.87	-0.92	1.19	0.07	1.38	0.00

B.	deep_1	deep_2	deep_3	deep_4	unreflective_1	unreflective_2	unreflective_3	cynicism_1	cynicism_2	cynicism_3	exhaustion_1	exhaustion_2	exhaustion_3	exhaustion_4	score_div100	study_year
**deep_1**	0.00															
**deep_2**	3.02	0.00														
**deep_3**	-0.24	-1.90	0.00													
**deep_4**	-0.52	0.53	0.75	0.00												
**unreflective_1**	-1.11	-0.17	-2.18	-1.28	0.00											
**unreflective_2**	-0.42	1.38	-1.47	-0.77	0.85	0.00										
**unreflective_3**	1.09	-0.58	2.04	2.40	-0.51	-0.35	0.00									
**cynicism_1**	0.23	-0.36	0.46	0.19	0.82	0.89	2.16	0.00								
**cynicism_2**	-0.42	0.37	0.30	-0.68	-2.90	0.70	1.28	-2.66	0.00							
**cynicism_3**	0.39	-0.39	-0.32	0.33	-2.10	1.17	-0.42	-0.73	3.38	0.00						
**exhaustion_1**	-1.34	-1.10	-0.45	-0.16	2.00	-0.41	3.12	5.41	0.92	-0.82	0.00					
**exhaustion_2**	-1.41	-0.49	-1.44	-1.32	-0.24	-3.03	0.82	1.66	-0.14	-0.43	-0.03	0.00				
**exhaustion_3**	-0.30	-0.52	0.80	0.45	-2.96	-2.18	-1.12	0.62	-0.32	-2.35	-1.01	1.11	0.00			
**exhaustion_4**	-0.35	0.13	1.53	1.40	0.19	0.13	-0.17	0.84	-1.11	-1.53	-2.57	0.62	1.89	0.00		
**score_div100**	0.40	-0.84	-0.40	-0.18	-0.49	0.25	0.85	1.30	2.37	-0.16	1.63	-0.60	1.25	0.21	-0.41	
**study_year**	0.66	-0.06	-0.68	0.14	-0.49	0.98	0.64	1.30	1.99	1.35	1.85	-0.03	0.91	0.41	-0.41	0.00

Supplementary Table 7A. Standardized coefficients of the full structural equation model fitted on the unweighted complete data (N=248). lhs: left-hand side variable, rhs: right-hand side variable, op: operator, est.std: standardized estimate, se: standard error, z: z statistics, ci.lower: lower bound and ci.upper: upper bound of the 95% confidence interval of the standardized estimate, h2: communalities, =~: factor loadings, ~~: covariation, NA: not available. score_div100: VetRepos Test score divided by 100. This transformation does not affect the modeling but brings the variances closer to each other. Non-significant estimates are colored gray.

lhs	op	rhs	est.std	se	z	pvalue	ci.lower	ci.upper	h2
f_deep	=~	deep_1	0.50	0.06	8.37	0.0E+00	0.38	0.61	0.25
f_deep	=~	deep_2	0.37	0.07	5.50	3.8E-08	0.24	0.50	0.14
f_deep	=~	deep_3	0.84	0.04	18.60	0.0E+00	0.75	0.92	0.70
f_deep	=~	deep_4	0.81	0.05	17.43	0.0E+00	0.72	0.90	0.66
f_unreflective	=~	unreflective_1	0.62	0.06	11.19	0.0E+00	0.51	0.73	0.39
f_unreflective	=~	unreflective_2	0.53	0.07	8.18	2.2E-16	0.41	0.66	0.28
f_unreflective	=~	unreflective_3	0.68	0.06	11.94	0.0E+00	0.57	0.79	0.46
f_cynicism	=~	cynicism_1	0.70	0.05	15.54	0.0E+00	0.61	0.79	0.49
f_cynicism	=~	cynicism_2	0.86	0.04	23.53	0.0E+00	0.78	0.93	0.73
f_cynicism	=~	cynicism_3	0.74	0.04	18.19	0.0E+00	0.66	0.82	0.54
f_exhaustion	=~	exhaustion_1	0.64	0.05	13.32	0.0E+00	0.55	0.74	0.41
f_exhaustion	=~	exhaustion_2	0.67	0.05	12.62	0.0E+00	0.57	0.78	0.46
f_exhaustion	=~	exhaustion_3	0.53	0.06	8.76	0.0E+00	0.41	0.64	0.28
f_exhaustion	=~	exhaustion_4	0.69	0.05	14.59	0.0E+00	0.60	0.78	0.48
score_div100	~	f_deep	0.16	0.06	2.46	1.4E-02	0.03	0.28	NA
score_div100	~	study_year	0.68	0.03	23.52	0.0E+00	0.62	0.73	NA
score_div100	~	f_unreflective	-0.25	0.11	-2.24	2.5E-02	-0.47	-0.03	NA
score_div100	~	f_cynicism	0.00	0.07	0.01	1.0E+00	-0.14	0.14	NA
score_div100	~	f_exhaustion	0.10	0.10	1.02	3.1E-01	-0.09	0.30	NA
f_cynicism	~~	f_exhaustion	0.54	0.06	8.43	0.0E+00	0.41	0.66	NA
f_unreflective	~~	f_cynicism	0.56	0.08	6.76	1.4E-11	0.39	0.72	NA
f_unreflective	~~	f_exhaustion	0.63	0.07	8.54	0.0E+00	0.49	0.78	NA
f_deep	~~	f_unreflective	-0.29	0.08	-3.53	4.1E-04	-0.46	-0.13	NA
deep_1	~~	deep_1	0.75	0.06	12.77	0.0E+00	0.64	0.87	NA
deep_2	~~	deep_2	0.86	0.05	17.46	0.0E+00	0.77	0.96	NA
deep_3	~~	deep_3	0.30	0.08	4.01	6.0E-05	0.15	0.45	NA
deep_4	~~	deep_4	0.34	0.08	4.55	5.3E-06	0.20	0.49	NA
unreflective_1	~~	unreflective_1	0.61	0.07	8.80	0.0E+00	0.48	0.75	NA
unreflective_2	~~	unreflective_2	0.72	0.07	10.27	0.0E+00	0.58	0.85	NA
unreflective_3	~~	unreflective_3	0.54	0.08	7.03	2.1E-12	0.39	0.69	NA
cynicism_1	~~	cynicism_1	0.51	0.06	8.09	6.7E-16	0.39	0.63	NA
cynicism_2	~~	cynicism_2	0.27	0.06	4.30	1.7E-05	0.15	0.39	NA
cynicism_3	~~	cynicism_3	0.46	0.06	7.68	1.6E-14	0.34	0.57	NA
exhaustion_1	~~	exhaustion_1	0.59	0.06	9.44	0.0E+00	0.46	0.71	NA
exhaustion_2	~~	exhaustion_2	0.54	0.07	7.54	4.6E-14	0.40	0.69	NA
exhaustion_3	~~	exhaustion_3	0.72	0.06	11.51	0.0E+00	0.60	0.85	NA
exhaustion_4	~~	exhaustion_4	0.52	0.07	7.96	1.8E-15	0.39	0.65	NA
score_div100	~~	score_div100	0.45	0.04	11.32	0.0E+00	0.37	0.53	NA
f_deep	~~	f_deep	1.00	0.00	NA	NA	1.00	1.00	NA
f_unreflective	~~	f_unreflective	1.00	0.00	NA	NA	1.00	1.00	NA
f_cynicism	~~	f_cynicism	1.00	0.00	NA	NA	1.00	1.00	NA
f_exhaustion	~~	f_exhaustion	1.00	0.00	NA	NA	1.00	1.00	NA
f_deep	~~	f_cynicism	-0.19	0.09	-2.07	3.9E-02	-0.37	-0.01	NA
f_deep	~~	f_exhaustion	0.08	0.08	1.10	2.7E-01	-0.07	0.23	NA
study_year	~~	study_year	1.00	0.00	NA	NA	1.00	1.00	NA

Supplementary Table 7B. Standardized coefficients of the full structural equation model fitted on the weighted data (N=243). lhs: left-hand side variable, rhs: right-hand side variable, op: operator, est.std: standardized estimate, se: standard error, z: z statistics, ci.lower: lower bound and ci.upper: upper bound of the 95% confidence interval of the standardized estimate, h2: communalities, =~: factor loadings, ~~: covariation, NA: not available. score_div100: VetRepos Test score divided by 100. This transformation does not affect the modeling but brings the variances closer to each other. Non-significant estimates are colored gray.

lhs	op	rhs	est.std	se	z	pvalue	ci.lower	ci.upper	h2
f_deep	=~	deep_1	0.48	0.06	7.46	0.0E+00	0.35	0.60	0.23
f_deep	=~	deep_2	0.34	0.07	4.90	0.0E+00	0.20	0.47	0.11
f_deep	=~	deep_3	0.90	0.05	17.19	0.0E+00	0.79	1.00	0.80
f_deep	=~	deep_4	0.79	0.05	15.32	0.0E+00	0.68	0.89	0.62
f_unreflective	=~	unreflective_1	0.62	0.06	10.92	0.0E+00	0.51	0.73	0.38
f_unreflective	=~	unreflective_2	0.52	0.07	7.77	0.0E+00	0.39	0.65	0.27
f_unreflective	=~	unreflective_3	0.68	0.06	11.45	0.0E+00	0.56	0.79	0.46
f_cynicism	=~	cynicism_1	0.70	0.04	15.88	0.0E+00	0.62	0.79	0.49
f_cynicism	=~	cynicism_2	0.88	0.04	24.15	0.0E+00	0.81	0.96	0.78
f_cynicism	=~	cynicism_3	0.76	0.04	18.65	0.0E+00	0.68	0.84	0.58
f_exhaustion	=~	exhaustion_1	0.65	0.05	13.98	0.0E+00	0.56	0.74	0.42
f_exhaustion	=~	exhaustion_2	0.68	0.05	13.48	0.0E+00	0.58	0.77	0.46
f_exhaustion	=~	exhaustion_3	0.56	0.06	10.13	0.0E+00	0.45	0.67	0.31
f_exhaustion	=~	exhaustion_4	0.74	0.04	16.55	0.0E+00	0.65	0.83	0.55
score_div100	~	f_deep	0.11	0.06	1.70	9.0E-02	-0.02	0.24	NA
score_div100	~	study_year	0.66	0.03	21.26	0.0E+00	0.59	0.72	NA
score_div100	~	f_unreflective	-0.27	0.11	-2.40	2.0E-02	-0.49	-0.05	NA
score_div100	~	f_cynicism	0.03	0.08	0.41	6.8E-01	-0.12	0.18	NA
score_div100	~	f_exhaustion	0.11	0.10	1.08	2.8E-01	-0.09	0.31	NA
f_cynicism	~~	f_exhaustion	0.52	0.06	8.60	0.0E+00	0.40	0.64	NA
f_unreflective	~~	f_cynicism	0.52	0.08	6.35	0.0E+00	0.36	0.68	NA
f_unreflective	~~	f_exhaustion	0.64	0.07	9.09	0.0E+00	0.50	0.78	NA
f_deep	~~	f_unreflective	-0.18	0.09	-2.12	3.0E-02	-0.35	-0.01	NA
deep_1	~~	deep_1	0.77	0.06	12.69	0.0E+00	0.65	0.89	NA
deep_2	~~	deep_2	0.89	0.05	18.96	0.0E+00	0.79	0.98	NA
deep_3	~~	deep_3	0.20	0.09	2.12	3.0E-02	0.01	0.38	NA
deep_4	~~	deep_4	0.38	0.08	4.77	0.0E+00	0.23	0.54	NA
unreflective_1	~~	unreflective_1	0.62	0.07	8.75	0.0E+00	0.48	0.75	NA
unreflective_2	~~	unreflective_2	0.73	0.07	10.64	0.0E+00	0.60	0.87	NA
unreflective_3	~~	unreflective_3	0.54	0.08	6.81	0.0E+00	0.39	0.70	NA
cynicism_1	~~	cynicism_1	0.51	0.06	8.15	0.0E+00	0.38	0.63	NA
cynicism_2	~~	cynicism_2	0.22	0.06	3.37	0.0E+00	0.09	0.35	NA
cynicism_3	~~	cynicism_3	0.42	0.06	6.80	0.0E+00	0.30	0.54	NA
exhaustion_1	~~	exhaustion_1	0.58	0.06	9.65	0.0E+00	0.46	0.70	NA
exhaustion_2	~~	exhaustion_2	0.54	0.07	8.01	0.0E+00	0.41	0.68	NA
exhaustion_3	~~	exhaustion_3	0.69	0.06	11.18	0.0E+00	0.57	0.81	NA
exhaustion_4	~~	exhaustion_4	0.45	0.07	6.74	0.0E+00	0.32	0.58	NA
score_div100	~~	score_div100	0.50	0.04	12.12	0.0E+00	0.42	0.58	NA
f_deep	~~	f_deep	1.00	0.00	NA	NA	1.00	1.00	NA
f_unreflective	~~	f_unreflective	1.00	0.00	NA	NA	1.00	1.00	NA
f_cynicism	~~	f_cynicism	1.00	0.00	NA	NA	1.00	1.00	NA
f_exhaustion	~~	f_exhaustion	1.00	0.00	NA	NA	1.00	1.00	NA
f_deep	~~	f_cynicism	-0.13	0.09	-1.45	1.5E-01	-0.30	0.05	NA
f_deep	~~	f_exhaustion	0.14	0.07	1.94	5.0E-02	0.00	0.28	NA
study_year	~~	study_year	1.00	0.00	NA	NA	1.00	1.00	NA

Supplementary Table 8. Parameter estimates (mean value, upper and lower bound for 95% confidence interval, 1000 iterations) of measurement model fitted on down-sampled data (N=173 for each iteration).

lhs	op	rhs	est.std			se			z		
			mean	lower	upper	mean	lower	upper	mean	lower	upper
f_deep	=~	deep_1	0.49	0.43	0.55	0.07	0.06	0.07	7.35	6.00	8.57
f_deep	=~	deep_2	0.36	0.30	0.41	0.08	0.08	0.09	4.43	3.55	5.28
f_deep	=~	deep_3	0.85	0.80	0.90	0.05	0.04	0.07	16.84	11.71	22.05
f_deep	=~	deep_4	0.79	0.75	0.83	0.06	0.05	0.07	13.35	11.19	15.63
f_unreflective	=~	unreflective_1	0.64	0.59	0.68	0.07	0.06	0.07	9.31	7.95	10.77
f_unreflective	=~	unreflective_2	0.52	0.47	0.58	0.08	0.07	0.09	6.54	5.49	7.70
f_unreflective	=~	unreflective_3	0.67	0.61	0.72	0.07	0.07	0.08	9.32	7.93	10.74
f_cynicism	=~	cynicism_1	0.71	0.67	0.75	0.05	0.05	0.06	13.71	11.74	15.72
f_cynicism	=~	cynicism_2	0.88	0.83	0.92	0.04	0.03	0.05	23.10	17.67	28.79
f_cynicism	=~	cynicism_3	0.75	0.71	0.78	0.05	0.04	0.05	16.05	13.83	18.44
f_exhaustion	=~	exhaustion_1	0.63	0.58	0.67	0.06	0.05	0.06	11.11	9.41	12.94
f_exhaustion	=~	exhaustion_2	0.66	0.62	0.70	0.06	0.06	0.07	10.93	9.20	12.42
f_exhaustion	=~	exhaustion_3	0.58	0.53	0.62	0.07	0.07	0.08	8.18	7.01	9.16
f_exhaustion	=~	exhaustion_4	0.74	0.69	0.79	0.05	0.04	0.06	15.59	12.66	18.64
deep_1	~~	deep_1	0.76	0.70	0.82	0.07	0.06	0.07	11.64	9.69	13.77
deep_2	~~	deep_2	0.87	0.83	0.91	0.06	0.05	0.07	15.24	12.57	18.56
deep_3	~~	deep_3	0.27	0.20	0.35	0.09	0.07	0.11	3.05	2.28	3.74
deep_4	~~	deep_4	0.38	0.31	0.44	0.09	0.08	0.11	4.03	3.17	4.80
unreflective_1	~~	unreflective_1	0.59	0.53	0.65	0.09	0.08	0.09	6.75	5.96	7.62
unreflective_2	~~	unreflective_2	0.73	0.67	0.78	0.08	0.08	0.09	8.74	7.60	10.02
unreflective_3	~~	unreflective_3	0.55	0.48	0.62	0.10	0.09	0.10	5.79	4.79	6.81
cynicism_1	~~	cynicism_1	0.49	0.43	0.56	0.07	0.07	0.08	6.66	5.80	7.58
cynicism_2	~~	cynicism_2	0.22	0.15	0.31	0.07	0.06	0.08	3.25	2.33	4.08
cynicism_3	~~	cynicism_3	0.44	0.39	0.50	0.07	0.07	0.07	6.37	5.69	7.12
exhaustion_1	~~	exhaustion_1	0.60	0.55	0.66	0.07	0.07	0.07	8.50	7.77	9.26
exhaustion_2	~~	exhaustion_2	0.56	0.51	0.62	0.08	0.08	0.09	6.90	6.23	7.61
exhaustion_3	~~	exhaustion_3	0.67	0.62	0.72	0.08	0.08	0.09	8.13	7.27	9.15
exhaustion_4	~~	exhaustion_4	0.45	0.37	0.52	0.07	0.07	0.08	6.28	5.37	7.21
f_deep	~~	f_deep	1.00	1.00	1.00	0.00	0.00	0.00	NA	NA	NA
f_unreflective	~~	f_unreflective	1.00	1.00	1.00	0.00	0.00	0.00	NA	NA	NA
f_cynicism	~~	f_cynicism	1.00	1.00	1.00	0.00	0.00	0.00	NA	NA	NA
f_exhaustion	~~	f_exhaustion	1.00	1.00	1.00	0.00	0.00	0.00	NA	NA	NA
f_deep	~~	f_unreflective	-0.28	-0.36	-0.20	0.10	0.10	0.11	-2.73	-3.62	-1.89
f_deep	~~	f_cynicism	-0.19	-0.27	-0.11	0.11	0.10	0.11	-1.80	-2.54	-1.03
f_deep	~~	f_exhaustion	0.10	0.05	0.16	0.09	0.09	0.10	1.11	0.49	1.74
f_unreflective	~~	f_cynicism	0.51	0.46	0.57	0.10	0.10	0.11	5.02	4.35	5.70
f_unreflective	~~	f_exhaustion	0.59	0.53	0.64	0.09	0.09	0.10	6.34	5.37	7.34
f_cynicism	~~	f_exhaustion	0.50	0.45	0.55	0.07	0.07	0.08	6.81	5.69	7.87

Supplementary Table 9. Parameter estimates (mean value, upper and lower bound for 95% confidence interval, 1000 iterations) of measurement model fitted on down-sampled data (N=173 for each iteration).

lhs	op	rhs	est.std			se			z		
			mean	lower	upper	mean	lower	upper	mean	lower	upper
f_deep	=~	deep_1	0.49	0.42	0.55	0.07	0.42	0.55	7.27	5.92	8.51
f_deep	=~	deep_2	0.35	0.29	0.41	0.08	0.29	0.40	4.35	3.46	5.20
f_deep	=~	deep_3	0.87	0.82	0.91	0.05	0.82	0.91	17.95	12.53	23.75
f_deep	=~	deep_4	0.78	0.74	0.82	0.06	0.74	0.81	13.21	11.21	15.38
f_unreflective	=~	unreflective_1	0.64	0.58	0.68	0.07	0.58	0.68	9.48	7.99	11.16
f_unreflective	=~	unreflective_2	0.53	0.47	0.58	0.08	0.47	0.59	6.72	5.64	7.93
f_unreflective	=~	unreflective_3	0.67	0.61	0.72	0.07	0.61	0.72	9.45	8.01	10.91
f_cynicism	=~	cynicism_1	0.71	0.67	0.75	0.05	0.67	0.75	13.72	11.74	15.73
f_cynicism	=~	cynicism_2	0.88	0.83	0.93	0.04	0.83	0.92	23.05	17.64	28.77
f_cynicism	=~	cynicism_3	0.75	0.71	0.78	0.05	0.71	0.78	16.07	13.87	18.51
f_exhaustion	=~	exhaustion_1	0.63	0.58	0.67	0.06	0.58	0.67	11.11	9.38	12.94
f_exhaustion	=~	exhaustion_2	0.66	0.62	0.70	0.06	0.62	0.70	10.92	9.18	12.44
f_exhaustion	=~	exhaustion_3	0.58	0.53	0.62	0.07	0.53	0.62	8.17	7.02	9.17
f_exhaustion	=~	exhaustion_4	0.74	0.69	0.79	0.05	0.70	0.79	15.59	12.63	18.63
score_div100	~	f_deep	0.12	0.04	0.19	0.07	0.04	0.19	1.59	0.57	2.67
score_div100	~	study_year	0.65	0.62	0.67	0.04	0.62	0.67	16.50	14.94	18.09
score_div100	~	f_unreflective	-0.23	-0.32	-0.14	0.13	-0.32	-0.14	-1.81	-2.50	-1.09
score_div100	~	f_cynicism	0.03	-0.04	0.09	0.09	-0.04	0.09	0.29	-0.46	1.04
score_div100	~	f_exhaustion	0.06	-0.01	0.14	0.11	0.00	0.14	0.56	-0.11	1.20
f_cynicism	~~	f_exhaustion	0.50	0.45	0.55	0.07	0.45	0.55	6.81	5.69	7.86
f_unreflective	~~	f_cynicism	0.52	0.46	0.57	0.10	0.45	0.57	5.02	4.36	5.70
f_unreflective	~~	f_exhaustion	0.59	0.53	0.64	0.09	0.53	0.63	6.33	5.37	7.35
f_deep	~~	f_unreflective	-0.28	-0.36	-0.20	0.10	-0.35	-0.21	-2.75	-3.64	-1.95
deep_1	~~	deep_1	0.76	0.70	0.82	0.07	0.70	0.82	11.69	9.69	13.93
deep_2	~~	deep_2	0.88	0.83	0.92	0.06	0.84	0.91	15.66	12.83	19.33
deep_3	~~	deep_3	0.25	0.17	0.33	0.09	0.17	0.33	2.91	2.06	3.63
deep_4	~~	deep_4	0.39	0.33	0.45	0.09	0.34	0.45	4.26	3.41	5.08
unreflective_1	~~	unreflective_1	0.60	0.53	0.66	0.09	0.54	0.66	6.99	6.15	7.88
unreflective_2	~~	unreflective_2	0.72	0.66	0.78	0.08	0.65	0.78	8.70	7.59	9.99
unreflective_3	~~	unreflective_3	0.56	0.48	0.63	0.09	0.48	0.62	5.93	4.97	7.00
cynicism_1	~~	cynicism_1	0.49	0.43	0.56	0.07	0.44	0.55	6.66	5.81	7.58
cynicism_2	~~	cynicism_2	0.22	0.14	0.31	0.07	0.15	0.31	3.26	2.30	4.10
cynicism_3	~~	cynicism_3	0.44	0.39	0.50	0.07	0.39	0.50	6.35	5.68	7.11
exhaustion_1	~~	exhaustion_1	0.60	0.55	0.66	0.07	0.55	0.66	8.49	7.75	9.27
exhaustion_2	~~	exhaustion_2	0.56	0.51	0.62	0.08	0.51	0.62	6.90	6.23	7.61
exhaustion_3	~~	exhaustion_3	0.67	0.62	0.72	0.08	0.61	0.72	8.13	7.27	9.13
exhaustion_4	~~	exhaustion_4	0.45	0.37	0.52	0.07	0.37	0.51	6.27	5.36	7.21
score_div100	~~	score_div100	0.51	0.48	0.54	0.05	0.48	0.54	10.11	8.90	11.21
f_deep	~~	f_deep	1.00	1.00	1.00	0.00	1.00	1.00	NA	NA	NA
f_unreflective	~~	f_unreflective	1.00	1.00	1.00	0.00	1.00	1.00	NA	NA	NA
f_cynicism	~~	f_cynicism	1.00	1.00	1.00	0.00	1.00	1.00	NA	NA	NA
f_exhaustion	~~	f_exhaustion	1.00	1.00	1.00	0.00	1.00	1.00	NA	NA	NA
f_deep	~~	f_cynicism	-0.19	-0.27	-0.11	0.10	-0.27	-0.11	-1.80	-2.53	-1.02
f_deep	~~	f_exhaustion	0.10	0.05	0.16	0.09	0.04	0.16	1.12	0.50	1.75
study_year	~~	study_year	1.00	1.00	1.00	0.00	1.00	1.00	NA	NA	NA

Supplementary Table 10. The median response category (0–4) to each indicator item by each gender. Last four rows represent raw sum scores for the scales.

	males	females	difference
deep_1	2	2	0
deep_2	2	2	0
deep_3	3	3	0
deep_4	3	3	0
unreflective_1	1	1	0
unreflective_2	1	1	0
unreflective_3	1	1	0
cynicism_1	1	2	-1
cynicism_2	1	1	0
cynicism_3	0	1	-1
exhaustion_1	2	3	-1
exhaustion_2	1	1	0
exhaustion_3	1	2	-1
exhaustion_4	1	2	-1
raw_deep	10	10	0
raw_unreflective	3	4	-1
raw_cynicism	3	3	0
raw_exhaustion	4	8	-4
