# Supplementary material for: Knowledge attainment, learning approaches, and self-perceived study burnout among European veterinary students
Source: Front Vet Sci. 2024 Jul 18;11:1292750. doi: 10.3389/fvets.2024.1292750 (PMC11291358; doi:10.3389/fvets.2024.1292750)
Supplement: Supplementary file 2 [file Data_Sheet_2.pdf]

Knowledge attainment, learning approaches, and self-perceived study burnout among European veterinary students

Antti Iivanainen

2024-05-24

Table of contents

Brief description of the contents 2
Load required packages 3
Load observations data 3
Group variables, assign data to raw_data object and describe 4
Model structures 6
Preparation of weights 9
Fit models, exact fit test and global fit indices 10
Down sampling university F 14
Complete data with factor scores 18

Tables **19**
Table 1: Distribution of the participants 19
Table 2: Factor loadings on the learning approach indicator items (CFA & EFA) 23
Table 3: Reliability measures 24
Table 4: Factor loadings from EFA on burnout indicators 27
Table 5A: Global fit indices for the measurement and full SE models. Complete unweighted data. 27
Table 5B: Global fit indices for the measurement and full SE models. Weighted data. 29
Table 5C: Global fit indices, down-sampled data 31
Table 6A: Measurement invariance between study years 32
Table 6B: Measurement invariance between genders 34
Table S2A: Bivariate Spearman correlations between indicator items 35
Table S2B. Confidence intervals of the interitem correlation coefficients (1000 bootstrap iterations). 38
Table S3: Testing for normality. 39

Table S4: Measurement model, standardized parameters . . . 42
Table S5: Interitem residual correlation coefficients, fitted full model . . . 45
Table S6: Standardized interitem residual covariances, fitted full model . . . 47
Table S7. Full model, standardized parameters . . . 49
Table S8: Parameter estimates from measurement models fitted to downsampled data . . . 52
Table S9: Parameter estimates from full SE models fitted to downsampled data . . . 54
Table S10: Raw score medians by gender . . . 55

Figures 56
Figure 1: Unreflective approach items - information and probability as a function of θ . . . 56
Figure 2: VetRepos test score distribution . . . 59
Figure 3: Path diagram for the measurement model . . . 61
Figure 4: Sum scores of the indicator items . . . 61
Figure 5: Path diagram for the full model . . . 63
Figure 6: Standardized residual covariances of the fitted full model . . . 63
Figure 7: Comparison of parameter estimates from full SE models . . . 67
Figure 8: Levels of latent traits across study years . . . 71
Figure 9: Standardized levels of latent traits . . . 74
Figure S1: Model comparison: original vs. covariance term at cynicism_1~~exhaustion_1 . . . 76
Figure S2: Raw sumscores of learning approaches, cynicism and exhaustion in males and females 80

Brief description of the contents

This script documents the analyses of the paper and produces csv files for the tables and raw figure files. The figures for the paper were exported as svg files and polished using Inkscape. The csv tables were loaded to excel, formatted and copied to the manuscript file for final polishing. Supplementary Tables 2A and 2B were exported as pdf files.

The anonymized input data have been deposited to the Finnish Social Science Data Archive https://urn.fi/urn:nbn:fi:fsd:T-FSD3873. The data do not include information on the gender or the university of the participants. Therefore a good number of the analyses won't run. ***These are marked red color***. Part of the code is hidden for clarity but all code is included in the script file, which is available from figshare at https://dx.doi.org/10.6084/m9.figshare.25470436.

To recreate a static pdf document from the script file, launch R and run the command:

```
quarto::quarto_render(input="R-code.qmd", output_format="pdf")
```

Rendering the pdf document relies on Quarto and an up-to-date LaTeX installation, for example TinyTeX or TeX Live.

Load required packages

```
boot
corrplot
dplyr
dunn.test
effectsize
GGally
ggExtra
ggplot2
GPArotation
kableExtra
knitr
lavaan
lattice
latticeExtra
mirt
patchwork
psych
psychTools
rsvg
semPlot
semTools
stats
```

Load observations data

The public anonymous data file does not contain university and gender information (variables 1 and 2 on the table in page 5.

```
ANONYMOUS_DATA = "anonymous_data_VetRepos.csv"
data1 <- read.csv(
  ANONYMOUS_DATA,
  header=T,
  dec = '.',
  stringsAsFactors = T
)
```

Group variables, assign data to raw_data object and describe

```
scale_deep <- paste("deep_", 1:4, sep ='')
scale_unreflective4 <- paste("unreflective_", 1:4, sep ='')
scale_unreflective3 <- paste("unreflective_", 1:3, sep ='')
scale_cynicism <- paste("cynicism_", 1:3, sep = '')
scale_exhaustion <- paste("exhaustion_", 1:4, sep = '')
scale_inadequacy <- paste("inadequacy_", 1:2, sep = '')
scale_all <- c(
  scale_deep,
  scale_unreflective4,
  scale_cynicism,
  scale_exhaustion,
  scale_inadequacy
)


# divide score by 100 to align variance with the other observed variables
data1["score_div100"] <- data1["vetrepos_score"]/100

# raw sumscores
data1["raw_deep"] <- apply(data1[paste("deep_", 1:4, sep ='')], 1, sum)
data1["raw_unreflective4"] <- apply( # all four indicators
  data1[paste("unreflective_", 1:4, sep ='')], 1, sum
)
data1["raw_unreflective3"] <- apply( # indicators 1, 2 and 3
  data1[paste("unreflective_", 1:3, sep ='')], 1, sum
)
data1["raw_unreflective"] <- data1["raw_unreflective3"]
data1["raw_cynicism"] <- apply(data1[paste("cynicism_", 1:3, sep = '')], 1, sum)
data1["raw_exhaustion"] <- apply(data1[paste("exhaustion_", 1:4, sep = '')], 1, sum)
data1["raw_inadequacy"] <- apply(data1[paste("inadequacy_", 1:2, sep = '')], 1, sum)
data1["raw_burnout"] <- apply(
  data1[,c("raw_cynicism", "raw_exhaustion", "raw_inadequacy")],
  1,
  sum
)

# Assign everything to raw_data and describe
raw_data <- data1
```

Description of the rawdata

	vars	n	mean	sd	median	trimmed	mad	min	max	range	skew	kurtosis	se
university*	1	248	4.49	2.08	6.00	4.60	1.48	1.0	7	6.0	-0.47	-1.35	0.13
gender	2	248	1.89	0.34	2.00	1.98	0.00	1.0	3	2.0	-1.84	3.67	0.02
study_year	3	248	2.88	1.49	3.00	2.77	1.48	1.0	6	5.0	0.50	-0.80	0.09
vetrepos_score	4	248	507.48	92.51	508.00	507.16	103.04	290.0	700	410.0	0.03	-0.75	5.87
deep_1	5	248	2.19	0.78	2.00	2.24	1.48	0.0	4	4.0	-0.35	0.01	0.05
deep_2	6	248	2.22	0.87	2.00	2.25	1.48	0.0	4	4.0	-0.29	-0.67	0.06
deep_3	7	248	2.76	0.76	3.00	2.79	0.00	0.0	4	4.0	-0.71	0.67	0.05
deep_4	8	248	2.77	0.74	3.00	2.80	0.00	0.0	4	4.0	-0.73	0.85	0.05
unreflective_1	9	248	1.57	0.95	1.00	1.58	1.48	0.0	4	4.0	0.19	-0.88	0.06
unreflective_2	10	248	1.53	0.94	1.00	1.53	1.48	0.0	4	4.0	0.38	-0.60	0.06
unreflective_3	11	248	1.17	0.84	1.00	1.10	0.00	0.0	4	4.0	0.68	0.30	0.05
unreflective_4	12	248	2.87	0.81	3.00	2.94	0.00	0.0	4	4.0	-0.91	1.13	0.05
cynicism_1	13	248	1.54	1.04	1.00	1.51	1.48	0.0	4	4.0	0.22	-0.74	0.07
cynicism_2	14	248	1.01	1.03	1.00	0.88	1.48	0.0	4	4.0	0.85	-0.15	0.07
cynicism_3	15	248	0.94	1.04	1.00	0.79	1.48	0.0	4	4.0	0.87	-0.30	0.07
exhaustion_1	16	248	2.40	0.99	3.00	2.43	1.48	0.0	4	4.0	-0.50	-0.39	0.06
exhaustion_2	17	248	1.52	1.23	1.00	1.46	1.48	0.0	4	4.0	0.36	-1.03	0.08
exhaustion_3	18	248	2.19	1.00	2.00	2.19	1.48	0.0	4	4.0	-0.16	-0.64	0.06
exhaustion_4	19	248	1.61	1.14	1.00	1.59	1.48	0.0	4	4.0	0.20	-0.97	0.07
inadequacy_1	20	248	1.96	1.10	2.00	1.99	1.48	0.0	4	4.0	-0.07	-0.88	0.07
inadequacy_2	21	248	1.75	1.15	2.00	1.76	1.48	0.0	4	4.0	0.10	-1.05	0.07
score_div100	22	248	5.07	0.93	5.08	5.07	1.03	2.9	7	4.1	0.03	-0.75	0.06
raw_deep	23	248	9.94	2.33	10.00	10.02	1.48	0.0	16	16.0	-0.52	1.08	0.15
raw_unreflective4	24	248	7.13	2.40	7.00	7.08	2.97	1.0	14	13.0	0.23	-0.26	0.15
raw_unreflective3	25	248	4.27	2.09	4.00	4.20	1.48	0.0	11	11.0	0.40	-0.16	0.13
raw_unreflective	26	248	4.27	2.09	4.00	4.20	1.48	0.0	11	11.0	0.40	-0.16	0.13
raw_cynicism	27	248	3.48	2.64	3.00	3.29	2.97	0.0	12	12.0	0.60	-0.13	0.17
raw_exhaustion	28	248	7.72	3.26	8.00	7.70	2.97	1.0	16	15.0	0.10	-0.55	0.21
raw_inadequacy	29	248	3.71	1.87	4.00	3.73	2.97	0.0	8	8.0	0.02	-0.76	0.12
raw_burnout	30	248	14.92	6.33	15.00	14.84	5.93	1.0	34	33.0	0.12	-0.39	0.40

Model structures

- Measurement model
- Full structural equation model
- Full structural equation model with the covariance term cynicism_1~~exhaustion_1
- Measurement invariance for study years: constrained and unconstrained models
- Measurement invariance for genders: constrained and unconstrained models

```r
# measurement model
m_measurement = '
  f_deep =~ deep_1 + deep_2 + deep_3 + deep_4
  f_unreflective =~ unreflective_1 + unreflective_2 + unreflective_3
  f_cynicism =~ cynicism_1 + cynicism_2 + cynicism_3
  f_exhaustion =~ exhaustion_1 + exhaustion_2 + exhaustion_3 + exhaustion_4
'

# full structural equation model
m_full_SEM <- '
  # measurement model
  f_deep =~ deep_1 + deep_2 + deep_3 + deep_4
  f_unreflective =~ unreflective_1 + unreflective_2 + unreflective_3
  f_cynicism =~ cynicism_1 + cynicism_2 + cynicism_3
  f_exhaustion =~ exhaustion_1 + exhaustion_2 + exhaustion_3 + exhaustion_4

  # regressions
  score_div100 ~ f_deep + study_year  + f_unreflective + f_cynicism + f_exhaustion

  # covariances
  f_cynicism ~~ f_exhaustion
  f_unreflective ~~ f_cynicism + f_exhaustion + f_deep
'

# full SEM with covariance term cynicism_1~~exhaustion_1
m_full_SEM_cov <- '
  # measurement model
  f_deep =~ deep_1 + deep_2 + deep_3 + deep_4
  f_unreflective =~ unreflective_1 + unreflective_2 + unreflective_3
  f_cynicism =~ cynicism_1 + cynicism_2 + cynicism_3
  f_exhaustion =~ exhaustion_1 + exhaustion_2 + exhaustion_3 + exhaustion_4

  # regressions
```

```
  score_div100 ~ f_deep + study_year  + f_unreflective + f_cynicism + f_exhaustion

  # covariances
  f_cynicism ~~ f_exhaustion
  f_unreflective ~~ f_cynicism + f_exhaustion + f_deep
  cynicism_1 ~~ exhaustion_1
'

# models for assessing measurement invariance
m_constrained_syear <- '
  # measurement model
  f_deep =~ deep_1 + deep_2 + deep_3 + deep_4
  f_unreflective =~ unreflective_1 + unreflective_2 + unreflective_3
  f_cynicism =~ cynicism_1 + cynicism_2 + cynicism_3
  f_exhaustion =~ exhaustion_1 + exhaustion_2 + exhaustion_3 + exhaustion_4

  # regressions
  score_div100 ~ f_deep + study_year  + f_unreflective + f_cynicism + f_exhaustion

  # mimic regressions
  f_deep ~ 0*study_year
  f_unreflective ~ 0*study_year
  f_cynicism ~ 0*study_year
  f_exhaustion ~ 0*study_year

  # covariances
  f_cynicism ~~ f_exhaustion
  f_unreflective ~~ f_cynicism + f_exhaustion + f_deep
'

m_unconstrained_syear <- '
  # measurement model
  f_deep =~ deep_1 + deep_2 + deep_3 + deep_4
  f_unreflective =~ unreflective_1 + unreflective_2 + unreflective_3
  f_cynicism =~ cynicism_1 + cynicism_2 + cynicism_3
  f_exhaustion =~ exhaustion_1 + exhaustion_2 + exhaustion_3 + exhaustion_4

  # regressions
  score_div100 ~ f_deep + study_year  + f_unreflective + f_cynicism + f_exhaustion
```

```
  # mimic regressions
  f_deep ~ study_year
  f_unreflective ~ study_year
  f_cynicism ~ study_year
  f_exhaustion ~ study_year

  # covariances
  f_cynicism ~~ f_exhaustion
  f_unreflective ~~ f_cynicism + f_exhaustion + f_deep
'

m_constrained_gender <- '
  # measurement model
  f_deep =~ deep_1 + deep_2 + deep_3 + deep_4
  f_unreflective =~ unreflective_1 + unreflective_2 + unreflective_3
  f_cynicism =~ cynicism_1 + cynicism_2 + cynicism_3
  f_exhaustion =~ exhaustion_1 + exhaustion_2 + exhaustion_3 + exhaustion_4

  # regressions
  score_div100 ~ f_deep + study_year  + f_unreflective + f_cynicism + f_exhaustion

  # mimic regressions
  f_deep ~ 0*gender
  f_unreflective ~ 0*gender
  f_cynicism ~ 0*gender
  f_exhaustion ~ 0*gender

  # covariances
  f_cynicism ~~ f_exhaustion
  f_unreflective ~~ f_cynicism + f_exhaustion + f_deep
'

m_unconstrained_gender <- '
  # measurement model
  f_deep =~ deep_1 + deep_2 + deep_3 + deep_4
  f_unreflective =~ unreflective_1 + unreflective_2 + unreflective_3
  f_cynicism =~ cynicism_1 + cynicism_2 + cynicism_3
  f_exhaustion =~ exhaustion_1 + exhaustion_2 + exhaustion_3 + exhaustion_4

  # regressions
```

```
  score_div100 ~ f_deep + study_year  + f_unreflective + f_cynicism + f_exhaustion

  # mimic regressions
  f_deep ~ gender
  f_unreflective ~ gender
  f_cynicism ~ gender
  f_exhaustion ~ gender

  # covariances
  f_cynicism ~~ f_exhaustion
  f_unreflective ~~ f_cynicism + f_exhaustion + f_deep
'
```

Preparation of weights

The goal is to control the effect of university F. University E is removed to avoid introducing an overly strong emphasis on its five students.

The following code chunk won't run on anonymous data.

```
# raw data exluding university E
data_wt <- subset(raw_data, university != 'E')
data_wt$university <- factor(
  data_wt$university,
  levels = c('A', 'B', 'C', 'D', 'F', 'G')
)

# Calculate weights as the ratio of desired to observed proportions
group_table <- table(data_wt$university)
desired_proportions <- rep(
  1/length(levels(data_wt$university)),
  length(levels(data_wt$university))
)
weights <- desired_proportions / (group_table / sum(group_table))
data_wt$weights <- as.numeric(weights[as.character(data_wt$university)])

table_weights <- data.frame(cbind(
  count = table(data_wt$university),
  weights)
)
```

```r
table_weights["weighted count"] <- with(table_weights, count*weights)
table_weights <- rbind(table_weights, sum = apply(table_weights, 2, sum))
```

Weights per university

	count	weights	weighted count
A	31	1.31	40.5
B	32	1.27	40.5
C	27	1.50	40.5
D	18	2.25	40.5
F	105	0.39	40.5
G	30	1.35	40.5
sum	243	8.06	243.0

Fit models, exact fit test and global fit indices

- unweighted and weighted data
- full SE model fails the exact fit test
- full SEM with vs. without covariance term between cynicism_1 and exhaustion_1

```r
# global fit indices
global_fit_stats <- c(
  'rmsea.robust',
  'cfi.robust',
  'tli.scaled',
  'srmr'
)


### unweighted complete data
# measurement model, CFA
fit_measurement <-  cfa(
  model = m_measurement,
  data = raw_data,
  estimator = 'MLM',
  std.lv=T,
  fixed.x = 'default')
# exact-fit test
fitMeasures(fit_measurement, c("chisq.scaled", "df.scaled", "pvalue.scaled"))
```

```
 chisq.scaled     df.scaled pvalue.scaled 
      113.622        71.000         0.001 
```

```r
# full structural equation model
set.seed(1234)
fit_fullSEM <- sem(
  model = m_full_SEM, # full structural equation model
  data = raw_data,
  std.ov = T,
  mimic = 'lavaan',
  estimator = "MLM",
  fixed.x = 'default'
 )
# exact-fit test
fitMeasures(fit_fullSEM, c("chisq.scaled", "df.scaled", "pvalue.scaled"))
```

```
 chisq.scaled     df.scaled pvalue.scaled 
      148.202        95.000         0.000 
```

```r
# pvalue
fit_fullSEM@test$satorra.bentler$pvalue # 0.0003948651
```

```
[1] 0.0003948651
```

```r
# full structural equation model with covariance term cynicism_1~~exhaustion_1
set.seed(1234)
fit_fullSEM_cov <- sem(
  model = m_full_SEM_cov, # full structural equation model
  data = raw_data,
  std.ov = T,
  mimic = 'lavaan',
  estimator = "MLM",
  fixed.x = 'default'
 )
# exact-fit test
fitMeasures(fit_fullSEM_cov, c("chisq.scaled", "df.scaled", "pvalue.scaled")) # pvalue
```

```
 chisq.scaled     df.scaled pvalue.scaled 
      131.806        94.000         0.006 
```

```r
fit_fullSEM_cov@test$satorra.bentler$pvalue
```

```
[1] 0.00615839
```

```r
# comparison between fit_fullSEM and fit_fullSEM_cov
delta_cov_fit <- lavTestLRT(fit_fullSEM, fit_fullSEM_cov)

delta_chiq <- delta_cov_fit[2, 'Chisq'] - delta_cov_fit[1, 'Chisq']
pval <- pchisq(delta_chiq, 4, lower.tail = F)
delta_cov_fit[2, 'Chisq diff'] <- delta_chiq
delta_cov_fit[2, 'Pr(>Chisq)'] <- pval
delta_cov_fit # model comparison fit_fullSEM vs. fit_fullSEM_cov
```

```
Scaled Chi-Squared Difference Test (method = "satorra.bentler.2001")

lavaan NOTE:
    The "Chisq" column contains standard test statistics, not the
    robust test that should be reported per model. A robust difference
    test is a function of two standard (not robust) statistics.

                Df    AIC    BIC  Chisq Chisq diff Df diff Pr(>Chisq)
fit_fullSEM_cov 94 7332.0 7476.1 141.43
fit_fullSEM     95 7347.7 7488.3 159.11     17.676       1   0.001428 **
---
Signif. codes:  0 '***' 0.001 '**' 0.01 '*' 0.05 '.' 0.1 ' ' 1
```

The following code chunk won't run on anonymous data.

```r
### weighted data
# measurement model, weighted data excluding university E
set.seed(1234)
fit_weighted_measurement <-  cfa(
  model = m_measurement,
  data = data_wt, # raw_data without university E
  sampling.weights = "weights", # weight matrix
  estimator = 'MLM',
  std.lv=T,
```

```r
  fixed.x = 'default')
# exact-fit test
fitMeasures(fit_weighted_measurement, c("chisq.scaled", "df.scaled", "pvalue.scaled"))
```

```
 chisq.scaled     df.scaled pvalue.scaled 
      124.966        71.000         0.000 
```

```r
# pvalue
fit_weighted_measurement@test$satorra.bentler$pvalue
```

```
[1] 8.118573e-05
```

```r
# full SE model with weight matrix but without university E
set.seed(1234)
fit_weighted_fullSEM <- sem(
  model = m_full_SEM, # full structural equation model
  data = data_wt,
  sampling.weights = "weights",
  std.ov = T,
  mimic = 'lavaan',
  estimator = "MLM",
  fixed.x = 'default'
 )
# exact-fit test
fitMeasures(fit_weighted_fullSEM, c("chisq.scaled", "df.scaled", "pvalue.scaled"))
```

```
 chisq.scaled     df.scaled pvalue.scaled 
      147.914        95.000         0.000 
```

```r
# pvalue
fit_weighted_fullSEM@test$satorra.bentler$pvalue
```

```
[1] 0.0004177953
```

```r
# full SE model with covariance term cynicism_1~~exhaustion_1 and
# weight matrix but exluding data from university E
set.seed(1234)
fit_weighted_fullSEM_cov <- sem(
  model = m_full_SEM_cov, # full structural equation model
  data = data_wt,
  sampling.weights = "weights",
  std.ov = T,
  mimic = 'lavaan',
  estimator = "MLM",
  fixed.x = 'default'
 )

# exact-fit test
fitMeasures(fit_weighted_fullSEM_cov, c("chisq.scaled", "df.scaled", "pvalue.scaled"))
```

```
 chisq.scaled     df.scaled pvalue.scaled 
      126.109        94.000         0.015 
```

```r
# pvalue
fit_weighted_fullSEM_cov@test$satorra.bentler$pvalue
```

```
[1] 0.01516942
```

Down sampling university F

Down sampling of data, 1000 iterations. Sample size (N=173 including 30 from university F). The code won't run on anonymous data without university information.

Model fitting:

- measurement model
- full SE model

Global fit indices from fitted models:

- 'rmsea.robust',
- 'cfi.robust',
- 'tli.scaled',
- 'srmr'

Store global fit, local fit, and model parameters in R data objects:

- global fit
  - downsampled_data_fit_indices (measurement model)
  - downsampled_data_fit_indices_fullSEM (full SEM)
  - downsampled_data_fit_indices_fullSEM_cov (full SEM with cyn1~~exh1 covariance)
- local fit
  - downsampled_data_residual_correlations (full SEM)
  - downsampled_data_residual_correlations_cov (full SEM with cyn1~~exh1 covariance)
  - downsampled_data_standardized_residual_covariances (full SEM)
  - downsampled_data_standardized_residual_covariances_cov (full SEM with cyn1~~exh1 covariance)
- model parameters
  - downsampled_data_parameter_list (measurement model)
  - downsampled_data_parameter_list_fullSEM (full SEM)
  - downsampled_data_parameter_list_fullSEM_cov (full SEM with cyn1~~exh1 covariance)

The following code chunk won't run on anonymous data.

```
# global fit placeholders
downsampled_data_fit_indices <- list()
downsampled_data_fit_indices_fullSEM <- list()
downsampled_data_fit_indices_fullSEM_cov <- list()
# local fit placeholders (only for full SEM)
downsampled_data_residual_correlations <- list()
downsampled_data_residual_correlations_cov <- list()
downsampled_data_standardized_residual_covariances <- list()
downsampled_data_standardized_residual_covariances_cov <- list()
# parameter estimates placeholders
downsampled_data_parameter_list <- list()
downsampled_data_parameter_list_fullSEM <- list()
downsampled_data_parameter_list_fullSEM_cov <- list()

n_iterations <- n_iterations1
data <- raw_data
s = 30 # sample size
```

```r
for (i in 1:n_iterations) {
  # draw a sample from university F and
  # combine with the observations from the other universities
  uniF_idx <- which(data$university == 'F')
  sampled_idx <- sample(uniF_idx, length(uniF_idx) * (s/length(uniF_idx)))
  downsampled_data <- data[c(sampled_idx, which(data$university != 'F')), ]

  # Fit the measurement model to the down-sampled dataset
  fit_downsampled <-  cfa(
    model = m_measurement, # measurement model
    data = downsampled_data,
    estimator = 'MLM',
    std.lv=T,
    fixed.x = 'default'
  )

  # Fit the full SE model to down-sampled dataset
  fit_downsampled_fullSEM <- sem(
    model = m_full_SEM, # full structural equation model
    data = downsampled_data,
    std.ov = T,
    mimic = 'lavaan',
    estimator = "MLM",
    fixed.x = 'default'
  )

  # Fit the full SE model with covariance term to the down-sampled dataset
  fit_downsampled_fullSEM_cov <- sem(
    model = m_full_SEM_cov, # full SEM with cyn1~~exh1 covariance
    data = downsampled_data,
    std.ov = T,
    mimic = 'lavaan',
    estimator = "MLM",
    fixed.x = 'default'
  )

  # Save global fit indices, residuals and parameter estimates
  downsampled_data_fit_indices [[i]] <- fitMeasures(
    fit_downsampled,
    global_fit_stats
```

```
  )
  downsampled_data_fit_indices_fullSEM [[i]] <- fitMeasures(
    fit_downsampled_fullSEM,
    global_fit_stats
  )
  downsampled_data_residual_correlations[[i]] <- lavResiduals(
    fit_downsampled_fullSEM,
    type = 'cor'
  )$cov
  downsampled_data_standardized_residual_covariances[[i]] <- lavResiduals(
    fit_downsampled_fullSEM,
    type = 'raw'
  )$cov.z
  downsampled_data_parameter_list[[i]] <- standardizedSolution(
    fit_downsampled,
    output = 'data.frame'
  )
  downsampled_data_parameter_list_fullSEM[[i]] <- standardizedSolution(
    fit_downsampled_fullSEM,
    output = 'data.frame'
  )
  downsampled_data_fit_indices_fullSEM_cov[[i]] <- fitMeasures(
    fit_downsampled_fullSEM_cov,
    global_fit_stats
  )
  downsampled_data_residual_correlations_cov[[i]] <- lavResiduals(
    fit_downsampled_fullSEM_cov,
    type = 'cor'
  )$cov
  downsampled_data_standardized_residual_covariances_cov[[i]] <- lavResiduals(
    fit_downsampled_fullSEM_cov,
    type = 'raw'
  )$cov.z
  downsampled_data_parameter_list_fullSEM_cov[[i]] <- standardizedSolution(
    fit_downsampled_fullSEM_cov,
    output = 'data.frame'
  )
}
```

Complete data with factor scores

```r
lavaan_traits <- lavPredict(
  object = fit_fullSEM,
  type = 'lv'
)

complete_data <- cbind(
  raw_data,
  lavaan_traits
)
```

Description of the latent traits

	vars	n	mean	sd	median	trimmed	mad	min	max	range	skew	kurtosis	se
f_deep	1	248	0	0.45	0.11	0.02	0.24	-1.90	0.92	2.82	-0.59	0.98	0.03
f_unreflective	2	248	0	0.54	-0.05	-0.01	0.50	-1.17	1.57	2.74	0.29	-0.23	0.03
f_cynicism	3	248	0	0.64	-0.07	-0.05	0.74	-0.93	2.07	3.00	0.60	-0.25	0.04
f_exhaustion	4	248	0	0.57	-0.01	0.00	0.64	-1.20	1.59	2.79	0.11	-0.54	0.04

Tables

Table 1: Distribution of the participants

The following code chunk won't run on anonymous data.

```
# Table 1A
t1a <- table(raw_data['university'])
s <- sum(t1a)
attr(s, which = 'names') <- "sum"
table1A <- t(append(t1a, s))
```

Table 1A. Distribution of students by universities

A	B	C	D	E	F	G	sum
31	32	27	18	5	105	30	248

```
# Table 1B
data_table1B <- subset(raw_data, gender != 3)
mB <- matrix(
  data = addmargins(xtabs(~  gender + study_year, data=data_table1B)),
  byrow=F,
  nrow=3,
  dimnames = list(c('males', 'females', 'sum'), c(as.character(1:6), "sum"))
)
table1B <- data.frame(mB)
colnames(table1B) = attr(mB, "dimnames")[[2]]
```

Table 1B. Gender by study year

	1	2	3	4	5	6	sum
males	4	8	3	6	5	3	29
females	44	64	41	33	23	12	217
sum	48	72	44	39	28	15	246

```
# Table 1C obs/exp number of students by study year and university

data <- xtabs(~ university + study_year, raw_data)
(chisq <- chisq.test(data, simulate.p.value = T))
```

```
    Pearson's Chi-squared test with simulated p-value (based on 2000
    replicates)

data:  data
X-squared = 116.43, df = NA, p-value = 0.0004998
```

```r
mC <- matrix(
  data=ftable(round(data/chisq$expected, 2)),
  byrow = FALSE,
  nrow = 7,
  dimnames = list(LETTERS[1:7], 1:6)
)
table1C <- data.frame(mC)
colnames(table1C) <-  attr(mC, "dimnames")[[2]]
```

Table 1C. Observed/expected number of students by study year and university

	study year					
	1	2	3	4	5	6
A	1.14	0.44	1.27	1.23	1.71	0.53
B	0.32	0.32	1.23	1.39	1.38	4.13
C	0.00	0.38	1.04	2.59	1.64	1.84
D	0.28	1.51	0.63	0.71	1.97	0.92
E	0.00	0.68	1.13	2.54	0.00	3.31
F	1.88	1.36	0.86	0.30	0.25	0.00
G	0.00	1.36	1.13	1.27	1.48	0.55

Comparison between universities

```r
# Differences of the student profiles between universities
categories <- levels(raw_data$university)
alpha = 0.05

pairwise_comparison <- data.frame(
  university_1 = character(),
  university_2 = character(),
  chisq_stats = double(),
  pvalue = double()
)
```

```r
combinations <- combn(categories, 2)
for (i in 1:ncol(combinations)) {
  subset <- raw_data[raw_data$university %in% combinations[, i], ]
  set.seed(1234)
  chi_test_result <- chisq.test(
    simulate.p.value = T,
    xtabs(~ university + study_year, subset)[combinations[,i], ]
  )
  pairwise_comparison <- rbind(
    pairwise_comparison,
    c(
      combinations[1, i],
      combinations[2, i],
      chi_test_result$statistic,
      chi_test_result$p.value
    )
  )
}

colnames(pairwise_comparison) <- c(
  "university_1",
  "university_2",
  "chisq_statistic",
  "pvalue"
)
pairwise_comparison$chisq_statistic <- round(
  as.double(pairwise_comparison$chisq_statistic),
  2
)
pairwise_comparison$pvalue <- as.double(pairwise_comparison$pvalue)
pairwise_comparison$adj_pvalue <- with(pairwise_comparison, p.adjust(pvalue, "fdr"))
pairwise_comparison$significant_at_alpha <- with(
  pairwise_comparison,
  ifelse(adj_pvalue < alpha, "TRUE", "")
)

# placeholder matrix for the pairwise chisq statistics
mat <- matrix(
  NA,
  nrow = length(categories),
```

```r
  ncol = length(categories),
  dimnames = list(categories, categories)
)

# Fill the lower triangle with the pairwise values
for (i in 1:nrow(pairwise_comparison)) {
  # indices
  row_index <- which(categories == pairwise_comparison$university_1[i])
  col_index <- which(categories == pairwise_comparison$university_2[i])

  # lower triangle
  if (row_index > col_index) {
    mat[row_index, col_index] <- pairwise_comparison[i,"chisq_statistic" ]
  } else if (row_index < col_index) {
    mat[col_index, row_index] <- pairwise_comparison[i, "chisq_statistic"]
  }
}
```

Chi statistics from pairwise comparisons between universities

	A	B	C	D	E	F
B	8.52					
C	9.81	5.11				
D	8.13	10.63	10.52			
E	5.27	2.18	1.46	5.04		
F	26.99	56.76	58.73	22.01	32.87	
G	11.15	12.95	7.83	3.11	4.13	29.13

```r
# significant pairwise comparisons
significant_differences <- subset(pairwise_comparison, significant_at_alpha == "TRUE")
```

Significant differences at alpha = 0.05

university_1	university_2	chisq_statistic	pvalue	adj_pvalue	significant_at_alpha
A	F	26.99	0.0004998	0.0026237	TRUE
B	F	56.76	0.0004998	0.0026237	TRUE
B	G	12.95	0.0144928	0.0434783	TRUE
C	F	58.73	0.0004998	0.0026237	TRUE
D	F	22.01	0.0014993	0.0052474	TRUE
E	F	32.87	0.0009995	0.0041979	TRUE
F	G	29.13	0.0004998	0.0026237	TRUE

Table 2: Factor loadings on the learning approach indicator items (CFA & EFA)

```
# CFA
m_approaches = ' # measurement model for learning approaches
  deep =~ deep_1 + deep_2 + deep_3 + deep_4
  unreflective =~ unreflective_1 + unreflective_2 + unreflective_3 + unreflective_4
'

data_tableX = raw_data[c(scale_deep, scale_unreflective4)]
fit_approaches_cfa <-  cfa(
  model = m_approaches,
  data = data_tableX,
  std.ov = T,
  estimator = 'MLM',
  fixed.x = FALSE
)

tableXA <- standardizedSolution(fit_approaches_cfa, output='data.frame')
tableX <- subset(tableXA[,c(1,2,3,8,4,9)], op == "=~")

# EFA
fit_approaches_efa = efa(data_tableX, 2, output = 'lavaan')
tableXB <- standardizedSolution(fit_approaches_efa, output='data.frame')
f1 <- subset(tableXB[,c(1,2,3,8,4,9)], op == "=~" & lhs == 'f1')[c(4:6)]
f2 <- subset(tableXB[,c(1,2,3,8,4,9)], op == "=~" & lhs == 'f2')[c(4:6)]

# Table
tableX_rownames <- subset(tableXB, op =="=~" & lhs == 'f1')$rhs
tableX <- cbind(tableX[,-c(1,2)], f1)
tableX <- cbind(tableX, f2)
tableX <- round(tableX[,-1],2)
rownames(tableX) <- tableX_rownames
colnames(tableX) <- c(
  "ci.lower",
  "f.cfa",
  "ci.upper",
  "ci.lower",
  "f1.efa",
  "ci.upper",
  "ci.lower",
```

```r
  "f2.efa",
  "ci.upper"
)
table2 <- tableX
```

Table 2. Factor loadings on the learning approach indicator items

	ci.lower	f.cfa	ci.upper	ci.lower	f1.efa	ci.upper	ci.lower	f2.efa	ci.upper
deep_1	0.38	0.50	0.62	0.39	0.51	0.62	-0.11	0.02	0.16
deep_2	0.24	0.37	0.50	0.25	0.38	0.51	-0.12	0.02	0.17
deep_3	0.73	0.82	0.92	0.74	0.82	0.91	-0.09	-0.02	0.06
deep_4	0.73	0.82	0.92	0.73	0.81	0.90	-0.06	0.00	0.06
unreflective_1	0.61	0.75	0.88	-0.04	0.01	0.05	0.63	0.77	0.90
unreflective_2	0.41	0.54	0.66	-0.17	-0.04	0.09	0.37	0.51	0.65
unreflective_3	0.43	0.56	0.69	-0.13	-0.02	0.10	0.40	0.54	0.67
unreflective_4	0.15	0.28	0.40	0.14	0.28	0.42	0.24	0.39	0.53

Table 3: Reliability measures

- Cronbach's α
- McDonald's ω_{tot}
- Person Separation Index (PSI)

$$PSI = \frac{true\ variance}{total\ variance} = 1 - \frac{mean\ error\ variance\ of\ location\ \theta\ for\ each\ person}{total\ variance\ for\ the\ person\ location\ \theta}$$

```r
table3_scales = list(
  scale_deep,
  scale_unreflective4,
  scale_unreflective3, # items 1-3 only
  scale_cynicism,
  scale_exhaustion,
  scale_inadequacy
)

# Cronbach's alpha
table3_results = list()
```

```r
for(s in table3_scales){
  table3_results <-
    append(table3_results,
           psych::alpha(raw_data[s])$feldt
    )
}

alpha_m <- matrix(data = unlist(table3_results),
                  nrow = 6,
                  ncol = 4,
                  byrow = T,
                  dimnames = list(
                    list("deep",
                         "unreflective4",
                         "unreflective", # items 1-3
                         "cynicism",
                         "exhaustion",
                         "inadequacy"
                    ),
                    list("lower_ci", "alpha", "upper_ci", "r_bar")
                  )
)

table3 <- data.frame(alpha_m)[-4]  # Table 3, alpha only

# McDonald's omega
fun_omega <- function(d,ind){
  data_boot = d[ind,]
  fit_boot <-  cfa(
    model = m_measurement,
    data = data_boot,
    estimator = 'MLM',
    std.lv=T,
    fixed.x = FALSE)
  o <- omegaFromSem(fit = fit_boot,
                    m = data_boot,
                    plot = FALSE)
  return (o$omega.tot)
}
```

```r
set.seed(1234)
bootout_omega <- boot(
  data = raw_data,
  statistic = fun_omega,
  R = n_iterations1 # 1000
)

ci_results <- boot.ci(
  bootout_omega,
  conf = 0.95,
  type = 'perc'
)

table3 <- rbind(
  table3,
  c(ci_results$percent[4], ci_results$t0,ci_results$percent[5])
)
rownames(table3)[7] <- "omega.tot"

# append Person Separation Index
# For data (N = 1948), see Schaper et al. 2023.
# PSI = 0.86 [0.85-0.87], bootstrapped 95% confidence interval (10000 iterations)

table3 <- rbind(table3, c(0.85, 0.86, 0.87))
rownames(table3)[8] <- "pers.sep.index"
colnames(table3)[2] <- "reliability_est"
table3 <- round(table3, 2)
```

Table 3. Reliability measures

	lower_ci	reliability_est	upper_ci
deep	0.66	0.72	0.77
unreflective4	0.52	0.61	0.68
unreflective	0.56	0.64	0.71
cynicism	0.76	0.80	0.84
exhaustion	0.67	0.73	0.78
inadequacy	0.42	0.55	0.65
omega.tot	0.77	0.81	0.84
pers.sep.index	0.85	0.86	0.87

Table 4: Factor loadings from EFA on burnout indicators

```
data_table4A <- raw_data[c(scale_cynicism, scale_exhaustion, scale_inadequacy)]
data_table4B <- raw_data[c(scale_cynicism, scale_exhaustion)]

burnout_loadings <- data.frame(
  efa(data_table4A, 3)$loadings,
  efa(data_table4A, 2)$loadings,
  rbind(
    efa(data_table4B, 2)$loadings,
    inadequacy_1 = NA,
    inadequacy_2 = NA
  )
)

colnames(burnout_loadings) <- c(
  paste("dim3_f", 1:3, sep = ''),
  paste("dim2A_f", 1:2, sep = ''),
  paste("dim2B_f", 1:2, sep = '')
)
```

Table 4. Factor loadings from EFA on burnout indicators

	dim3_f1	dim3_f2	dim3_f3	dim2A_f1	dim2A_f2	dim2B_f1	dim2B_f2
cynicism_1	0.56	0.26	0.01	0.50	0.34	0.57	0.24
cynicism_2	0.88	-0.10	0.01	0.76	0.10	0.82	0.04
cynicism_3	0.89	0.01	-0.22	0.86	-0.08	0.83	-0.12
exhaustion_1	0.00	0.66	0.19	0.01	0.73	0.13	0.55
exhaustion_2	0.03	0.01	0.64	0.01	0.61	0.03	0.67
exhaustion_3	-0.08	0.03	0.60	-0.12	0.60	-0.08	0.61
exhaustion_4	0.01	-0.12	0.80	-0.01	0.61	-0.01	0.69
inadequacy_1	0.16	0.67	0.00	0.16	0.58	NA	NA
inadequacy_2	0.43	0.21	0.00	0.39	0.25	NA	NA

Table 5A: Global fit indices for the measurement and full SE models. Complete unweighted data.

```
my_statistic3 <- function(fitted_object){
  return(fitMeasures(fitted_object, global_fit_stats))
```

```r
}

# Measurement model
# lavaan WARNING: 1 bootstrap run resulted in a nonadmissible (n) solution.
bootout_lavaan_measurement <- bootstrapLavaan(
  object = fit_measurement,
  R = n_iterations1,  # 1000
  type = 'yuan',
  FUN = my_statistic3,
  iseed = 1234 )
```

```
Warning in bootstrapLavaan(object = fit_measurement, R = n_iterations1, :
lavaan WARNING: 1 bootstrap run resulted in a nonadmissible (n) solution.
```

```r
df_meas = data.frame(bootout_lavaan_measurement)

ci_lavaan_meas = data.frame(
  lower_ci = numeric(),
  higher_ci = numeric()
)
for (i in 1:4){
  ci_lavaan_meas <- rbind(
    ci_lavaan_meas,
    quantile(df_meas[,i], c(0.025, 0.975))
  )
}
ci_lavaan_meas <- cbind(ci_lavaan_meas, fitMeasures(fit_measurement, global_fit_stats))
colnames(ci_lavaan_meas) <- c("lower_ci", "upper_ci", "estimate")
rownames(ci_lavaan_meas) <- global_fit_stats
ci_lavaan_meas <- ci_lavaan_meas[,c(1,3,2)]

# Full SE model
bootout_lavaan <- bootstrapLavaan(
  object = fit_fullSEM,
  R = n_iterations1, # 1000
  type = 'yuan',
  FUN = my_statistic3,
  iseed = 1234 )

df = data.frame(bootout_lavaan)
```

```
ci_lavaan = data.frame(
  lower_ci = numeric(),
  higher_ci = numeric()
)
for (i in 1:4){
  ci_lavaan <- rbind(
    ci_lavaan,
    quantile(df[,i], c(0.025, 0.975))
  )
}
ci_lavaan <- cbind(ci_lavaan, fitMeasures(fit_fullSEM, global_fit_stats))
colnames(ci_lavaan) <- c("lower_ci", "upper_ci", "estimate")
rownames(ci_lavaan) <- global_fit_stats
ci_lavaan <- ci_lavaan[,c(1,3,2)] ##

# combine into a single table
table5A <- round(ci_lavaan_meas,6)
table5A <- cbind (table5A, round(ci_lavaan,6))
```

Table 5A. Global fit indices, unweighted complete data

	measurement model			full SE model		
	lower_ci	estimate	upper_ci	lower_ci	estimate	upper_ci
rmsea.robust	0.030546	0.051343	0.076442	0.033433	0.049237	0.070772
cfi.robust	0.894980	0.950499	0.982705	0.898682	0.949762	0.977536
tli.scaled	0.859026	0.933884	0.976861	0.867250	0.935051	0.971004
srmr	0.045045	0.054436	0.067894	0.048997	0.056350	0.070598

Table 5B: Global fit indices for the measurement and full SE models. Weighted data.

The following code chunk won't run on anonymous data.

```
my_statistic3 <- function(fitted_object){
  return(fitMeasures(fitted_object, global_fit_stats))
}

# Measurement model
# lavaan WARNING: 8 bootstrap run resulted in a nonadmissible (n) solution.
bootout_lavaan_measurement <- bootstrapLavaan(
```

```r
  object = fit_weighted_measurement,
  R = n_iterations1, # 1000
  type = 'yuan',
  FUN = my_statistic3,
  iseed = 1234 )
```

```
Warning in bootstrapLavaan(object = fit_weighted_measurement, R =
n_iterations1, : lavaan WARNING: 8 bootstrap runs resulted in nonadmissible (n)
solutions.
```

```r
df_meas = data.frame(bootout_lavaan_measurement)

ci_lavaan_meas = data.frame(
  lower_ci = numeric(),
  higher_ci = numeric()
)
for (i in 1:4){
  ci_lavaan_meas <- rbind(
    ci_lavaan_meas,
    quantile(df_meas[,i], c(0.025, 0.975))
  )
}
ci_lavaan_meas <- cbind(
  ci_lavaan_meas,
  fitMeasures(fit_weighted_measurement, global_fit_stats)
)
colnames(ci_lavaan_meas) <- c("lower_ci", "upper_ci", "estimate")
rownames(ci_lavaan_meas) <- global_fit_stats
ci_lavaan_meas <- ci_lavaan_meas[,c(1,3,2)]

# Full SE model
# lavaan WARNING: 7 bootstrap run resulted in a nonadmissible (n) solution.
bootout_lavaan <- bootstrapLavaan(
  object = fit_weighted_fullSEM,
  R = n_iterations1, # 1000
  type = 'yuan',
  FUN = my_statistic3,
  iseed = 1234 )
```

```
Warning in bootstrapLavaan(object = fit_weighted_fullSEM, R = n_iterations1, :
```

```
lavaan WARNING: 7 bootstrap runs resulted in nonadmissible (n) solutions.
```

```
df = data.frame(bootout_lavaan)

ci_lavaan = data.frame(
  lower_ci = numeric(),
  higher_ci = numeric()
)
for (i in 1:4){
  ci_lavaan <- rbind(
    ci_lavaan,
    quantile(df[,i], c(0.025, 0.975))
  )
}
ci_lavaan <- cbind(ci_lavaan, fitMeasures(fit_weighted_fullSEM, global_fit_stats))
colnames(ci_lavaan) <- c("lower_ci", "upper_ci", "estimate")
rownames(ci_lavaan) <- global_fit_stats
ci_lavaan <- ci_lavaan[,c(1,3,2)]

# combine into a single table
table5B <- round(ci_lavaan_meas,6)
table5B <- cbind (table5B, round(ci_lavaan,6))
```

Table 5B. Global fit indices, weighted data.

	measurement model			full SE model		
	lower_ci	estimate	upper_ci	lower_ci	estimate	upper_ci
rmsea.robust	0.042512	0.059563	0.092646	0.037545	0.050652	0.083275
cfi.robust	0.855572	0.938584	0.970613	0.868338	0.948213	0.972305
tli.scaled	0.812743	0.921334	0.961989	0.830231	0.934322	0.964288
srmr	0.049758	0.059491	0.080001	0.051986	0.058919	0.079079

Table 5C: Global fit indices, down-sampled data

The following code chunk won't run on anonymous data.

```
# measurement model
indices <- downsampled_data_fit_indices[[1]]
for (i in 2:n_iterations){
```

```
  indices <- cbind(indices, downsampled_data_fit_indices[[i]])
}
colnames(indices) <- 1:n_iterations

table5Cmeas <- apply(indices, 1, quantile, c(0.025, 0.975))
table5Cmeas <- rbind(means = table5Cmeas, apply(indices, 1, mean))[c(3,1,2),]
rownames(table5Cmeas)[1] <- "mean"

# full SEM
indices <- downsampled_data_fit_indices_fullSEM[[1]]
for (i in 2:n_iterations){
  indices <- cbind(indices, downsampled_data_fit_indices_fullSEM[[i]])
}
colnames(indices) <- 1:n_iterations
table5CfullSEM <- apply(indices, 1, quantile, c(0.025, 0.975))
table5CfullSEM <- rbind(table5CfullSEM, apply(indices, 1, mean))[c(3,1,2),]
rownames(table5CfullSEM)[1] <- "mean"

# combine into a single table
table5C <- data.frame(cbind(t(table5Cmeas), t(table5CfullSEM)))[c(2,1,3,5,4,6)]
colnames(table5C) <- colnames(table5A)
```

Table 5C. Global fit indices, down-sampled data, 1000 iterations.

	measurement model			full SE model		
	lower_ci	estimate	upper_ci	lower_ci	estimate	upper_ci
rmsea.robust	0.0374997	0.0501804	0.0623035	0.0293632	0.0433377	0.0561114
cfi.robust	0.9296487	0.9530494	0.9745723	0.9349271	0.9592552	0.9818047
tli.scaled	0.9041417	0.9361820	0.9649276	0.9138940	0.9464964	0.9756501
srmr	0.0571531	0.0623561	0.0681436	0.0574568	0.0627865	0.0684916

Table 6A: Measurement invariance between study years

```
# measurement invariance, study year
set.seed(1234)
fit_constrained_syear <- sem(
  model = m_constrained_syear,
  data <- raw_data,
```

```
  std.ov = T,
  estimator = "MLM",
  fixed.x = 'default'
)

set.seed(1234)
fit_unconstrained_syear <- sem(
  model = m_unconstrained_syear,
  data <- raw_data,
  std.ov = T,
  estimator = "MLM",
  fixed.x = 'default'
)

table6A <- lavTestLRT(
  fit_unconstrained_syear,
  fit_constrained_syear
) # deltaChisq = 3.781 not 4.058

delta_chiq <- table6A[2, 'Chisq'] - table6A[1, 'Chisq']
pval <- pchisq(delta_chiq, 4, lower.tail = F)
table6A[2, 'Chisq diff'] <- delta_chiq
table6A[2, 'Pr(>Chisq)'] <- pval
```

Table 6A. Chi-squared difference test on MI across study years

	Df	AIC	BIC	Chisq	Chisq diff	Df diff	Pr(>Chisq)
fit_unconstrained_syear	93	7359.247	7506.811	166.6358	NA	NA	NA
fit_constrained_syear	97	7355.028	7488.539	170.4167	3.780879	4	0.436472

```
# delta-cfi
fitMeasures(fit_constrained_syear,'cfi.robust' ) -
  fitMeasures(fit_unconstrained_syear, 'cfi.robust')
```

```
cfi.robust
         0
```

Table 6B: Measurement invariance between genders

The following code chunk won't run on anonymous data.

```
# remove 2 students of gender 'other'
data6B <- subset(raw_data, gender !=3)
data6B$gender <- data6B$gender - 1
table(data6B$gender)
```

```
  0   1
 29 217
```

```
set.seed(1234)
fit_constrained_gender <- sem(
  model = m_constrained_gender,
  data <- data6B,
  std.ov = T,
  estimator = "MLM",
  fixed.x = 'default'
)

set.seed(1234)
fit_unconstrained_gender <- sem(
  model = m_unconstrained_gender,
  data <- data6B,
  std.ov = T,
  estimator = "MLM",
  fixed.x = 'default'
)

# chisq-difference test
table6B <- lavTestLRT(
  fit_constrained_gender,
  fit_unconstrained_gender
) # deltaChisq = 19.350 not 18.612

delta_chiq <- table6B[2, 'Chisq'] - table6B[1, 'Chisq']
pval <- pchisq(delta_chiq, 4, lower.tail = F)
```

```r
table6B[2, 'Chisq diff'] <- delta_chiq
table6B[2, 'Pr(>Chisq)'] <- pval
```

Table 6B. Chi-squared difference test on MI between genders

	Df	AIC	BIC	Chisq	Chisq diff	Df diff	Pr(>Chisq)
fit_unconstrained_gender	108	6592.741	6739.965	178.7771	NA	NA	NA
fit_constrained_gender	112	6604.091	6737.293	198.1272	19.35008	4	0.0006707

```r
# delta-cfi
fitMeasures(fit_constrained_gender,'cfi.robust' ) -
  fitMeasures(fit_unconstrained_gender, 'cfi.robust')
```

```
cfi.robust
    -0.013
```

Table S2A: Bivariate Spearman correlations between indicator items

```r
data_stable2 <- raw_data[, c(
  grep ("deep", colnames(raw_data)),
  grep ('unreflective[_4]', colnames(raw_data)),

  # corr btw burnout and unrefl_1-3 sum scores is 0.47
  # grep ('unreflective[_123]*[^_4]', colnames(raw_data)),

  grep ("cynicism", colnames(raw_data)),
  grep ("exhaustion", colnames(raw_data)),
  grep ("inadequacy", colnames(raw_data)),
  grep ("burnout", colnames(raw_data)))]

set.seed(1234)
cci <- cor.ci(
  data_stable2,
  n.iter = n_iterations1, # 1000
  method = 'sp',
  show.legend = FALSE,
  plot = FALSE
)
```

```r
corPlot(# table S2A
  main = '',
  adjust = T,
  pval = T,
  cci$rho,
  show.legend = FALSE,
  xlas = 2,
  MAR = 5,
  keep.par = TRUE
)
```

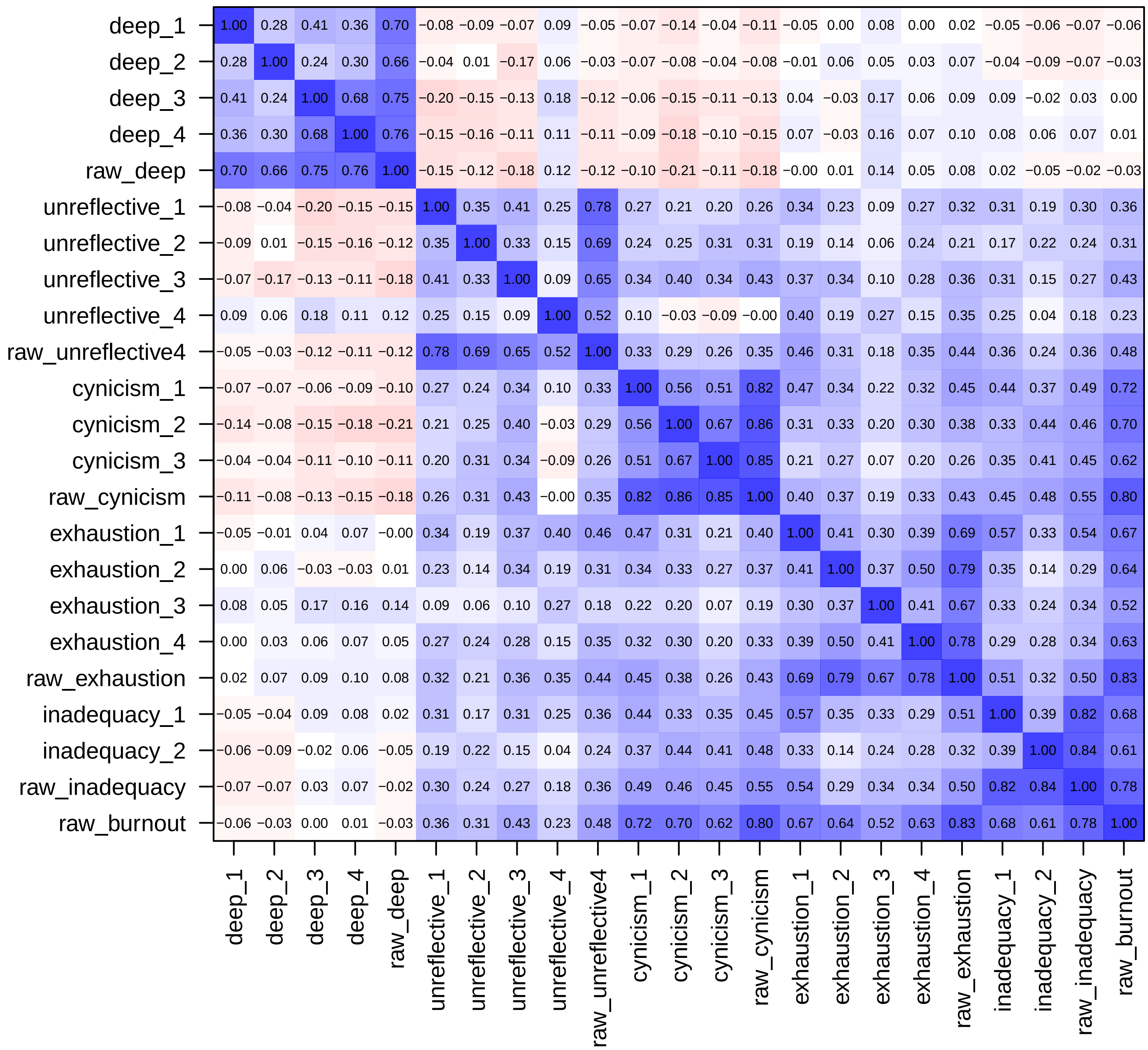
deep_1 1.00 0.28 0.41 0.36 0.70 −0.08 −0.09 −0.07 0.09 −0.05 −0.07 −0.14 −0.04 −0.11 −0.05 0.00 0.08 0.00 0.02 −0.05 −0.06 −0.07 −0.06
deep_2 0.28 1.00 0.24 0.30 0.66 −0.04 0.01 −0.17 0.06 −0.03 −0.07 −0.08 −0.04 −0.08 −0.01 0.06 0.05 0.03 0.07 −0.04 −0.09 −0.07 −0.03
deep_3 0.41 0.24 1.00 0.68 0.75 −0.20 −0.15 −0.13 0.18 −0.12 −0.06 −0.15 −0.11 −0.13 0.04 −0.03 0.17 0.06 0.09 0.09 −0.02 0.03 0.00
deep_4 0.36 0.30 0.68 1.00 0.76 −0.15 −0.16 −0.11 0.11 −0.11 −0.09 −0.18 −0.10 −0.15 0.07 −0.03 0.16 0.07 0.10 0.08 0.06 0.07 0.01
raw_deep 0.70 0.66 0.75 0.76 1.00 −0.15 −0.12 −0.18 0.12 −0.12 −0.10 −0.21 −0.11 −0.18 −0.00 0.01 0.14 0.05 0.08 0.02 −0.05 −0.02 −0.03
unreflective_1 −0.08 −0.04 −0.20 −0.15 −0.15 1.00 0.35 0.41 0.25 0.78 0.27 0.21 0.20 0.26 0.34 0.23 0.09 0.27 0.32 0.31 0.19 0.30 0.36
unreflective_2 −0.09 0.01 −0.15 −0.16 −0.12 0.35 1.00 0.33 0.15 0.69 0.24 0.25 0.31 0.31 0.19 0.14 0.06 0.24 0.21 0.17 0.22 0.24 0.31
unreflective_3 −0.07 −0.17 −0.13 −0.11 −0.18 0.41 0.33 1.00 0.09 0.65 0.34 0.40 0.34 0.43 0.37 0.34 0.10 0.28 0.36 0.31 0.15 0.27 0.43
unreflective_4 0.09 0.06 0.18 0.11 0.12 0.25 0.15 0.09 1.00 0.52 0.10 −0.03 −0.09 −0.00 0.40 0.19 0.27 0.15 0.35 0.25 0.04 0.18 0.23
raw_unreflective4 −0.05 −0.03 −0.12 −0.11 −0.12 0.78 0.69 0.65 0.52 1.00 0.33 0.29 0.26 0.35 0.46 0.31 0.18 0.35 0.44 0.36 0.24 0.36 0.48
cynicism_1 −0.07 −0.07 −0.06 −0.09 −0.10 0.27 0.24 0.34 0.10 0.33 1.00 0.56 0.51 0.82 0.47 0.34 0.22 0.32 0.45 0.44 0.37 0.49 0.72
cynicism_2 −0.14 −0.08 −0.15 −0.18 −0.21 0.21 0.25 0.40 −0.03 0.29 0.56 1.00 0.67 0.86 0.31 0.33 0.20 0.30 0.38 0.33 0.44 0.46 0.70
cynicism_3 −0.04 −0.04 −0.11 −0.10 −0.11 0.20 0.31 0.34 −0.09 0.26 0.51 0.67 1.00 0.85 0.21 0.27 0.07 0.20 0.26 0.35 0.41 0.45 0.62
raw_cynicism −0.11 −0.08 −0.13 −0.15 −0.18 0.26 0.31 0.43 −0.00 0.35 0.82 0.86 0.85 1.00 0.40 0.37 0.19 0.33 0.43 0.45 0.48 0.55 0.80
exhaustion_1 −0.05 −0.01 0.04 0.07 −0.00 0.34 0.19 0.37 0.40 0.46 0.47 0.31 0.21 0.40 1.00 0.41 0.30 0.39 0.69 0.57 0.33 0.54 0.67
exhaustion_2 0.00 0.06 −0.03 −0.03 0.01 0.23 0.14 0.34 0.19 0.31 0.34 0.33 0.27 0.37 0.41 1.00 0.37 0.50 0.79 0.35 0.14 0.29 0.64
exhaustion_3 0.08 0.05 0.17 0.16 0.14 0.09 0.06 0.10 0.27 0.18 0.22 0.20 0.07 0.19 0.30 0.37 1.00 0.41 0.67 0.33 0.24 0.34 0.52
exhaustion_4 0.00 0.03 0.06 0.07 0.05 0.27 0.24 0.28 0.15 0.35 0.32 0.30 0.20 0.33 0.39 0.50 0.41 1.00 0.78 0.29 0.28 0.34 0.63
raw_exhaustion 0.02 0.07 0.09 0.10 0.08 0.32 0.21 0.36 0.35 0.44 0.45 0.38 0.26 0.43 0.69 0.79 0.67 0.78 1.00 0.51 0.32 0.50 0.83
inadequacy_1 −0.05 −0.04 0.09 0.08 0.02 0.31 0.17 0.31 0.25 0.36 0.44 0.33 0.35 0.45 0.57 0.35 0.33 0.29 0.51 1.00 0.39 0.82 0.68
inadequacy_2 −0.06 −0.09 −0.02 0.06 −0.05 0.19 0.22 0.15 0.04 0.24 0.37 0.44 0.41 0.48 0.33 0.14 0.24 0.28 0.32 0.39 1.00 0.84 0.61
raw_inadequacy −0.07 −0.07 0.03 0.07 −0.02 0.30 0.24 0.27 0.18 0.36 0.49 0.46 0.45 0.55 0.54 0.29 0.34 0.34 0.50 0.82 0.84 1.00 0.78
raw_burnout −0.06 −0.03 0.00 0.01 −0.03 0.36 0.31 0.43 0.23 0.48 0.72 0.70 0.62 0.80 0.67 0.64 0.52 0.63 0.83 0.68 0.61 0.78 1.00
deep_1 deep_2 deep_3 deep_4 raw_deep unreflective_1 unreflective_2 unreflective_3 unreflective_4 raw_unreflective4 cynicism_1 cynicism_2 cynicism_3 raw_cynicism exhaustion_1 exhaustion_2 exhaustion_3 exhaustion_4 raw_exhaustion inadequacy_1 inadequacy_2 raw_inadequacy raw_burnout

Table S2B. Confidence intervals of the interitem correlation coefficients (1000 bootstrap iterations).

	deep_1	deep_2	deep_3	deep_4	raw_deep	unreflective_1	unreflective_2	unreflective_3	unreflective_4	raw_unreflective4	cynicism_1	cynicism_2	cynicism_3	raw_cynicism	exhaustion_1	exhaustion_2	exhaustion_3	exhaustion_4	raw_exhaustion	inadequacy_1	inadequacy_2	raw_inadequacy	raw_burnout
deep_1	1.00	0.40	0.51	0.45	0.76	−0.21	−0.22	−0.20	0.21	−0.19	−0.19	−0.26	−0.17	−0.23	−0.17	0.12	0.22	0.12	0.14	−0.17	−0.19	−0.19	−0.18
deep_2	0.16	1.00	0.36	0.41	0.73	−0.17	0.13	−0.29	0.19	−0.17	−0.19	−0.20	−0.16	−0.20	−0.13	0.19	0.18	0.15	0.19	−0.16	−0.21	−0.19	−0.14
deep_3	0.30	0.12	1.00	0.76	0.81	−0.32	−0.27	−0.25	0.31	−0.24	−0.17	−0.27	−0.24	−0.25	0.16	−0.15	0.29	0.17	0.21	0.22	−0.15	0.16	0.12
deep_4	0.25	0.19	0.58	1.00	0.81	−0.28	−0.28	−0.24	0.24	−0.24	−0.21	−0.31	−0.22	−0.28	0.20	−0.15	0.29	0.19	0.22	0.21	0.18	0.20	0.14
raw_deep	0.62	0.58	0.69	0.69	1.00	−0.28	−0.24	−0.31	0.25	−0.25	−0.22	−0.33	−0.24	−0.30	−0.12	0.13	0.27	0.17	0.20	0.15	−0.17	−0.15	−0.16
unreflective_1	0.04	0.09	−0.08	−0.02	−0.02	1.00	0.45	0.52	0.36	0.83	0.38	0.34	0.32	0.38	0.45	0.35	0.22	0.39	0.43	0.43	0.31	0.42	0.47
unreflective_2	0.03	−0.12	−0.03	−0.04	0.00	0.24	1.00	0.44	0.25	0.75	0.36	0.37	0.42	0.43	0.31	0.26	0.19	0.35	0.33	0.30	0.34	0.36	0.42
unreflective_3	0.06	−0.03	0.00	0.02	−0.05	0.29	0.21	1.00	0.21	0.72	0.45	0.52	0.45	0.54	0.47	0.45	0.22	0.40	0.47	0.42	0.28	0.39	0.54
unreflective_4	−0.04	−0.07	0.04	−0.03	−0.01	0.13	0.05	−0.04	1.00	0.60	0.22	−0.15	−0.21	−0.12	0.50	0.30	0.39	0.27	0.45	0.36	0.17	0.30	0.34
raw_unreflective4	0.07	0.10	0.01	0.02	0.02	0.71	0.61	0.56	0.43	1.00	0.45	0.42	0.38	0.47	0.55	0.42	0.29	0.45	0.54	0.47	0.36	0.48	0.58
cynicism_1	0.06	0.06	0.07	0.04	0.03	0.14	0.11	0.21	−0.01	0.21	1.00	0.65	0.60	0.87	0.56	0.45	0.34	0.43	0.55	0.55	0.48	0.59	0.78
cynicism_2	−0.01	0.05	−0.03	−0.05	−0.08	0.08	0.13	0.28	0.10	0.16	0.46	1.00	0.75	0.89	0.43	0.45	0.31	0.41	0.49	0.45	0.54	0.56	0.76
cynicism_3	0.08	0.08	0.02	0.03	0.02	0.08	0.19	0.21	0.03	0.14	0.40	0.58	1.00	0.88	0.33	0.38	0.19	0.32	0.37	0.47	0.51	0.55	0.69
raw_cynicism	0.01	0.05	−0.01	−0.02	−0.05	0.13	0.18	0.31	0.12	0.22	0.77	0.81	0.81	1.00	0.51	0.48	0.31	0.43	0.53	0.55	0.57	0.64	0.84
exhaustion_1	0.08	0.11	−0.08	−0.06	0.12	0.23	0.07	0.26	0.29	0.37	0.36	0.18	0.09	0.28	1.00	0.51	0.42	0.50	0.75	0.66	0.44	0.63	0.74
exhaustion_2	−0.12	−0.06	0.10	0.09	−0.11	0.11	0.01	0.22	0.07	0.18	0.22	0.20	0.15	0.26	0.31	1.00	0.48	0.59	0.84	0.46	0.26	0.40	0.71
exhaustion_3	−0.05	−0.08	0.03	0.03	0.01	−0.04	−0.07	−0.03	0.14	0.06	0.09	0.08	−0.06	0.07	0.18	0.25	1.00	0.52	0.75	0.45	0.36	0.46	0.61
exhaustion_4	−0.12	−0.09	−0.06	−0.05	−0.07	0.14	0.12	0.16	0.03	0.23	0.19	0.19	0.08	0.21	0.27	0.39	0.29	1.00	0.83	0.42	0.39	0.46	0.70
raw_exhaustion	−0.10	−0.05	−0.03	−0.02	−0.03	0.19	0.09	0.25	0.24	0.33	0.34	0.27	0.14	0.33	0.61	0.74	0.58	0.71	1.00	0.61	0.44	0.60	0.87
inadequacy_1	0.07	0.09	−0.04	−0.05	−0.11	0.18	0.05	0.18	0.14	0.25	0.33	0.22	0.24	0.33	0.47	0.23	0.20	0.17	0.40	1.00	0.50	0.86	0.76
inadequacy_2	0.07	0.03	0.11	−0.07	0.09	0.06	0.10	0.02	−0.08	0.12	0.24	0.33	0.30	0.37	0.21	0.01	0.12	0.16	0.20	0.26	1.00	0.87	0.69
raw_inadequacy	0.06	0.05	−0.09	−0.06	0.11	0.17	0.11	0.15	0.06	0.24	0.37	0.36	0.35	0.45	0.45	0.17	0.22	0.22	0.39	0.77	0.80	1.00	0.83
raw_burnout	0.07	0.09	−0.12	−0.12	0.09	0.24	0.19	0.32	0.12	0.37	0.64	0.63	0.54	0.76	0.58	0.55	0.41	0.55	0.78	0.60	0.52	0.71	1.00

Table S3: Testing for normality.

- Table S3A: VetRepos test score
- Table S3B: scales

Deep sampling for kurtosis bootstrapping requires setting `SAMPLING_DEPTH` accordingly if an accurate estimate of the p-value is desirable. This may push the resources of a personal computer. `CI_VALUE` may also need to be adjusted.

```
# for an accurate p-value estimation, depending on your computer resources:
SAMPLING_DEPTH = 2000 # change to desired depth, e.g. 10^7, or more
CI_VALUE = 0.95 # decrease e.g to 0.9 or 0.8

### Table S3A: normality of vetrepos score

# Shapiro-Wilk test fails on the VetRepos test score
shapiro.test(raw_data$vetrepos_score)
```

```
    Shapiro-Wilk normality test

data:  raw_data$vetrepos_score
W = 0.98552, p-value = 0.01292
```

```
# bootstrapped kurtosis (VetRepos test score)
fun_kurtosis <- function(d,ind){
  return (kurtosi(d[ind,]))
}

set.seed(1234)
bootout_kurtosis <- boot(
  data = raw_data['vetrepos_score'],
  statistic = fun_kurtosis,
  R = SAMPLING_DEPTH
)

n = length(which(sort(bootout_kurtosis$t) >= 0))
p_kurtosis = (2*n)/bootout_kurtosis$R

ci_kurtosis <- boot.ci(
```

```
  bootout_kurtosis,
  conf = CI_VALUE,
  type = 'perc'
)

vetrepos_kurtosis <- c(
  ci_kurtosis$percent[4],
  ci_kurtosis$t0,
  ci_kurtosis$percent[5],
  p_kurtosis
)

# bootstrapped skewness (VetRepos test score)
fun_skewness <- function(d,ind){
  return(skew(d[ind])['skew (g1)'])
}

set.seed(1234)
bootout_skewness <- boot(
  data = raw_data$vetrepos_score,
  statistic = fun_skewness,
  R = 2000
)

p_skew = 2*(1-(length(which(sort(bootout_skewness$t) >= 0)))/2000)

ci_skewness <- boot.ci(
  bootout_skewness,
  conf = 0.95,
  type = 'perc'
)

vetrepos_skewness <- c(
  ci_skewness$percent[4],
  ci_skewness$t0,
  ci_skewness$percent[5],
  p_skew
)

STable3A <- data.frame(
```

```r
  lower_ci = numeric(),
  estimate = numeric(),
  upper_ci = numeric(),
  pvalue = numeric()
)

STable3A <-rbind(
  rbind(STable3A,vetrepos_skewness),
  vetrepos_kurtosis
)
colnames(STable3A) <- c('lower_ci', 'estimate', 'upper_ci', "pvalue")
rownames(STable3A) <- c('skewness', 'kurtosis')
```

Table S3A. Kurtosis and skewness of the VetRepos score

	lower_ci	estimate	upper_ci	pvalue
skewness	-0.1521057	0.0301417	0.1993484	0.745
kurtosis	-0.9787786	-0.7477947	-0.4840059	0.000

```r
### Table S3B: normality of the scales

# Mardia's test on the scales, requires semTools
mardia_list <- list()
for (
  s in c(
    list(scale_all),
    list(scale_deep),
    list(scale_unreflective4),
    list(scale_unreflective3),
    list(scale_cynicism),
    list(scale_exhaustion),
    list(scale_inadequacy)
  )
){
  estimates <- c(mardiaKurtosis(raw_data[,c(s)]), mardiaSkew(raw_data[,c(s)]))
  mardia_list <- append(mardia_list, estimates)
}

matrix_mardia <- matrix(
  data=unlist(mardia_list), nrow=7, ncol=7, byrow = T,
```

```
  dimnames = c(
    list(
      c(
        "all_scales",
        "deep_approach",
        "unreflective_approach4",
        "unreflective_approach3",
        "cynicism",
        "exhaustion",
        "inadequacy"
      )
    ),
    list(c('b2d', 'z', 'p2', 'b1d', 'chi', 'df', 'p1'))
  )
)

stable3B <- data.frame(matrix_mardia)
```

Table S3B. Mardia's multivariate normality test on the scales

	b2d	z	p2	b1d	chi	df	p1
all_scales	344.915837	6.7894979	0.0000000	36.0018973	1488.078423	969	0.0000000
deep_approach	27.282787	3.7309365	0.0001908	2.1548234	89.066035	20	0.0000000
unreflective_approach4	24.136013	0.1545809	0.8771517	1.8033408	74.538088	20	0.0000000
unreflective_approach3	14.247097	-1.0823660	0.2790899	0.7139070	29.508155	10	0.0010305
cynicism	16.299374	1.8679680	0.0617665	2.0099971	83.079880	10	0.0000000
exhaustion	22.819720	-1.3414059	0.1797887	1.1498543	47.527309	20	0.0004954
inadequacy	6.765118	-2.4308677	0.0150627	0.0319093	1.318918	4	0.8581577

Table S4: Measurement model, standardized parameters

- Table S4A: unweighted complete data
- Table S4B: weighted data (This code won't run on the anonymous data.)

```
# Table S4A
STable4A <- standardizedSolution(fit_measurement, output = 'data.frame')
STable4A$h2 = NA

STable4A [STable4A$op == "=~", "h2"] <- STable4A [STable4A$op == "=~", "est.std"]^2
```

```r
STable4A <- cbind(
  STable4A[1:3],
  round(STable4A[4:ncol(STable4A)], 2)
)
```

Table S4A. Parameter estimates from the measurement model fitted on the unweighted data

lhs	op	rhs	est.std	se	z	pvalue	ci.lower	ci.upper	h2
f_deep	=~	deep_1	0.50	0.06	8.40	0.00	0.38	0.61	0.25
f_deep	=~	deep_2	0.38	0.07	5.63	0.00	0.25	0.51	0.14
f_deep	=~	deep_3	0.82	0.05	17.03	0.00	0.73	0.92	0.68
f_deep	=~	deep_4	0.82	0.05	17.07	0.00	0.73	0.92	0.68
f_unreflective	=~	unreflective_1	0.63	0.06	11.10	0.00	0.52	0.74	0.40
f_unreflective	=~	unreflective_2	0.53	0.07	8.00	0.00	0.40	0.66	0.28
f_unreflective	=~	unreflective_3	0.68	0.06	11.67	0.00	0.56	0.79	0.46
f_cynicism	=~	cynicism_1	0.70	0.05	15.54	0.00	0.61	0.79	0.49
f_cynicism	=~	cynicism_2	0.86	0.04	23.65	0.00	0.79	0.93	0.74
f_cynicism	=~	cynicism_3	0.73	0.04	18.10	0.00	0.65	0.81	0.54
f_exhaustion	=~	exhaustion_1	0.64	0.05	13.26	0.00	0.55	0.74	0.41
f_exhaustion	=~	exhaustion_2	0.68	0.05	12.69	0.00	0.57	0.78	0.46
f_exhaustion	=~	exhaustion_3	0.52	0.06	8.76	0.00	0.41	0.64	0.28
f_exhaustion	=~	exhaustion_4	0.69	0.05	14.68	0.00	0.60	0.78	0.48
deep_1	~~	deep_1	0.75	0.06	12.76	0.00	0.64	0.87	NA
deep_2	~~	deep_2	0.86	0.05	17.02	0.00	0.76	0.96	NA
deep_3	~~	deep_3	0.32	0.08	4.08	0.00	0.17	0.48	NA
deep_4	~~	deep_4	0.32	0.08	4.11	0.00	0.17	0.48	NA
unreflective_1	~~	unreflective_1	0.60	0.07	8.46	0.00	0.46	0.74	NA
unreflective_2	~~	unreflective_2	0.72	0.07	10.25	0.00	0.58	0.86	NA
unreflective_3	~~	unreflective_3	0.54	0.08	6.95	0.00	0.39	0.70	NA
cynicism_1	~~	cynicism_1	0.51	0.06	8.11	0.00	0.39	0.63	NA
cynicism_2	~~	cynicism_2	0.26	0.06	4.25	0.00	0.14	0.39	NA
cynicism_3	~~	cynicism_3	0.46	0.06	7.72	0.00	0.34	0.58	NA
exhaustion_1	~~	exhaustion_1	0.59	0.06	9.47	0.00	0.47	0.71	NA
exhaustion_2	~~	exhaustion_2	0.54	0.07	7.54	0.00	0.40	0.68	NA
exhaustion_3	~~	exhaustion_3	0.72	0.06	11.52	0.00	0.60	0.85	NA
exhaustion_4	~~	exhaustion_4	0.52	0.07	7.98	0.00	0.39	0.65	NA
f_deep	~~	f_deep	1.00	0.00	NA	NA	1.00	1.00	NA
f_unreflective	~~	f_unreflective	1.00	0.00	NA	NA	1.00	1.00	NA
f_cynicism	~~	f_cynicism	1.00	0.00	NA	NA	1.00	1.00	NA
f_exhaustion	~~	f_exhaustion	1.00	0.00	NA	NA	1.00	1.00	NA
f_deep	~~	f_unreflective	-0.29	0.08	-3.54	0.00	-0.46	-0.13	NA
f_deep	~~	f_cynicism	-0.19	0.09	-2.07	0.04	-0.37	-0.01	NA
f_deep	~~	f_exhaustion	0.08	0.08	1.09	0.28	-0.07	0.23	NA
f_unreflective	~~	f_cynicism	0.55	0.08	6.73	0.00	0.39	0.71	NA
f_unreflective	~~	f_exhaustion	0.63	0.07	8.53	0.00	0.49	0.78	NA
f_cynicism	~~	f_exhaustion	0.54	0.06	8.42	0.00	0.41	0.66	NA

```r
# The following code chunk won't run on anonymous data.
# Table S4B
STable4B <- standardizedSolution(fit_weighted_measurement, output = 'data.frame')
```

Table S4B. Parameter estimates from the measurement model fitted on the weighted data

lhs	op	rhs	est.std	se	z	pvalue	ci.lower	ci.upper	h2
f_deep	=~	deep_1	0.48	0.06	7.54	0.00	0.35	0.60	0.23
f_deep	=~	deep_2	0.34	0.07	4.99	0.00	0.21	0.48	0.12
f_deep	=~	deep_3	0.89	0.05	16.46	0.00	0.78	0.99	0.79
f_deep	=~	deep_4	0.79	0.05	15.34	0.00	0.69	0.89	0.63
f_unreflective	=~	unreflective_1	0.62	0.06	10.90	0.00	0.51	0.73	0.39
f_unreflective	=~	unreflective_2	0.51	0.07	7.67	0.00	0.38	0.64	0.26
f_unreflective	=~	unreflective_3	0.68	0.06	11.28	0.00	0.56	0.79	0.46
f_cynicism	=~	cynicism_1	0.70	0.04	15.87	0.00	0.62	0.79	0.49
f_cynicism	=~	cynicism_2	0.88	0.04	24.13	0.00	0.81	0.96	0.78
f_cynicism	=~	cynicism_3	0.76	0.04	18.66	0.00	0.68	0.84	0.58
f_exhaustion	=~	exhaustion_1	0.65	0.05	13.91	0.00	0.56	0.74	0.42
f_exhaustion	=~	exhaustion_2	0.68	0.05	13.54	0.00	0.58	0.77	0.46
f_exhaustion	=~	exhaustion_3	0.56	0.06	10.13	0.00	0.45	0.67	0.31
f_exhaustion	=~	exhaustion_4	0.74	0.04	16.63	0.00	0.66	0.83	0.55
deep_1	~~	deep_1	0.77	0.06	12.72	0.00	0.65	0.89	NA
deep_2	~~	deep_2	0.88	0.05	18.63	0.00	0.79	0.97	NA
deep_3	~~	deep_3	0.21	0.10	2.21	0.03	0.02	0.40	NA
deep_4	~~	deep_4	0.37	0.08	4.58	0.00	0.21	0.53	NA
unreflective_1	~~	unreflective_1	0.61	0.07	8.60	0.00	0.47	0.75	NA
unreflective_2	~~	unreflective_2	0.74	0.07	10.73	0.00	0.60	0.87	NA
unreflective_3	~~	unreflective_3	0.54	0.08	6.69	0.00	0.38	0.70	NA
cynicism_1	~~	cynicism_1	0.51	0.06	8.15	0.00	0.38	0.63	NA
cynicism_2	~~	cynicism_2	0.22	0.06	3.37	0.00	0.09	0.35	NA
cynicism_3	~~	cynicism_3	0.42	0.06	6.79	0.00	0.30	0.54	NA
exhaustion_1	~~	exhaustion_1	0.58	0.06	9.66	0.00	0.46	0.70	NA
exhaustion_2	~~	exhaustion_2	0.54	0.07	8.01	0.00	0.41	0.67	NA
exhaustion_3	~~	exhaustion_3	0.69	0.06	11.19	0.00	0.57	0.81	NA
exhaustion_4	~~	exhaustion_4	0.45	0.07	6.75	0.00	0.32	0.58	NA
f_deep	~~	f_deep	1.00	0.00	NA	NA	1.00	1.00	NA
f_unreflective	~~	f_unreflective	1.00	0.00	NA	NA	1.00	1.00	NA
f_cynicism	~~	f_cynicism	1.00	0.00	NA	NA	1.00	1.00	NA
f_exhaustion	~~	f_exhaustion	1.00	0.00	NA	NA	1.00	1.00	NA
f_deep	~~	f_unreflective	-0.18	0.09	-2.11	0.03	-0.35	-0.01	NA
f_deep	~~	f_cynicism	-0.13	0.09	-1.45	0.15	-0.30	0.05	NA
f_deep	~~	f_exhaustion	0.14	0.07	1.93	0.05	0.00	0.28	NA
f_unreflective	~~	f_cynicism	0.52	0.08	6.34	0.00	0.36	0.68	NA
f_unreflective	~~	f_exhaustion	0.64	0.07	9.07	0.00	0.50	0.78	NA
f_cynicism	~~	f_exhaustion	0.52	0.06	8.59	0.00	0.40	0.64	NA

Table S5: Interitem residual correlation coefficients, fitted full model

The following code chunk won't run on anonymous data.

```r
# Table S5A unweighted complete data
STable5A <- lavResiduals(fit_fullSEM, type = 'cor')$cov
range(STable5A)
```

```
[1] -0.1493163  0.2276201
```

Table S5A. Standardized residual correlations. Full SEM.

	deep_1	deep_2	deep_3	deep_4	unreflective_1	unreflective_2	unreflective_3	cynicism_1	cynicism_2	cynicism_3	exhaustion_1	exhaustion_2	exhaustion_3	exhaustion_4	score_div100	study_year
deep_1	0.000	0.153	-0.003	-0.018	0.002	-0.035	0.050	-0.008	-0.058	0.009	-0.102	-0.032	0.055	-0.016	0.049	0.090
deep_2	0.153	0.000	-0.044	0.013	0.027	0.047	-0.072	-0.030	-0.006	-0.014	-0.044	0.049	0.033	0.017	-0.071	-0.035
deep_3	-0.003	-0.044	0.000	0.005	-0.042	-0.010	0.032	0.055	0.013	-0.011	-0.028	-0.047	0.130	0.014	0.066	0.069
deep_4	-0.018	0.013	0.005	0.000	-0.016	-0.039	0.040	0.010	-0.027	0.014	-0.011	-0.065	0.111	0.009	0.052	0.106
unreflective_1	0.002	0.027	-0.042	-0.016	0.000	0.042	-0.006	0.028	-0.103	-0.067	0.108	-0.014	-0.095	0.008	-0.034	-0.075
unreflective_2	-0.035	0.047	-0.010	-0.039	0.042	0.000	-0.027	0.019	0.004	0.080	0.002	-0.097	-0.117	0.013	0.000	0.033
unreflective_3	0.050	-0.072	0.032	0.040	-0.006	-0.027	0.000	0.061	0.048	-0.010	0.107	0.037	-0.098	-0.012	-0.018	-0.035
cynicism_1	-0.008	-0.030	0.055	0.010	0.028	0.019	0.061	0.000	-0.016	-0.019	0.228	0.092	0.019	0.055	0.022	0.017
cynicism_2	-0.058	-0.006	0.013	-0.027	-0.103	0.004	0.048	-0.016	0.000	0.022	0.018	-0.020	-0.011	-0.027	0.084	0.080
cynicism_3	0.009	-0.014	-0.011	0.014	-0.067	0.080	-0.010	-0.019	0.022	0.000	-0.030	-0.033	-0.149	-0.081	-0.061	0.005
exhaustion_1	-0.102	-0.044	-0.028	-0.011	0.108	0.002	0.107	0.228	0.018	-0.030	0.000	-0.013	-0.023	-0.049	0.095	0.124
exhaustion_2	-0.032	0.049	-0.047	-0.065	-0.014	-0.097	0.037	0.092	-0.020	-0.033	-0.013	0.000	0.023	0.024	-0.070	-0.057
exhaustion_3	0.055	0.033	0.130	0.111	-0.095	-0.117	-0.098	0.019	-0.011	-0.149	-0.023	0.023	0.000	0.050	0.109	0.078
exhaustion_4	-0.016	0.017	0.014	0.009	0.008	0.013	-0.012	0.055	-0.027	-0.081	-0.049	0.024	0.050	0.000	-0.007	0.004
score_div100	0.049	-0.071	0.066	0.052	-0.034	0.000	-0.018	0.022	0.084	-0.061	0.095	-0.070	0.109	-0.007	0.000	0.012
study_year	0.090	-0.035	0.069	0.106	-0.075	0.033	-0.035	0.017	0.080	0.005	0.124	-0.057	0.078	0.004	0.012	0.000

```r
# The following code chunk won't run on anonymous data.

# Table S5B weighted data
STable5B <- lavResiduals(fit_weighted_fullSEM, type = 'cor')$cov
range(STable5B)
```

```
[1] -0.1373657  0.2563980
```

Table S5B. Standardized residual correlations. Full SEM, weighted data.

	deep_1	deep_2	deep_3	deep_4	unreflective_1	unreflective_2	unreflective_3	cynicism_1	cynicism_2	cynicism_3	exhaustion_1	exhaustion_2	exhaustion_3	exhaustion_4	score_div100	study_year
deep_1	0.000	0.166	-0.003	-0.014	-0.069	-0.024	0.074	0.013	-0.026	0.024	-0.086	-0.094	-0.019	-0.022	0.025	0.041
deep_2	0.166	0.000	-0.024	0.016	-0.010	0.084	-0.035	-0.021	0.021	-0.023	-0.062	-0.029	-0.030	0.007	-0.053	-0.004
deep_3	-0.003	-0.024	0.000	0.002	-0.104	-0.060	0.095	0.022	0.008	-0.016	-0.021	-0.066	0.047	0.060	-0.018	-0.046
deep_4	-0.014	0.016	0.002	0.000	-0.072	-0.036	0.129	0.009	-0.027	0.016	-0.008	-0.069	0.027	0.064	-0.009	0.009
unreflective_1	-0.069	-0.010	-0.104	-0.072	0.000	0.026	-0.009	0.034	-0.103	-0.092	0.102	-0.010	-0.137	0.008	-0.025	-0.031
unreflective_2	-0.024	0.084	-0.060	-0.036	0.026	0.000	-0.009	0.042	0.027	0.052	-0.019	-0.131	-0.109	0.006	0.013	0.059
unreflective_3	0.074	-0.035	0.095	0.129	-0.009	-0.009	0.000	0.090	0.049	-0.021	0.141	0.034	-0.054	-0.006	0.046	0.043
cynicism_1	0.013	-0.021	0.022	0.009	0.034	0.042	0.090	0.000	-0.016	-0.011	0.256	0.076	0.032	0.040	0.068	0.083
cynicism_2	-0.026	0.021	0.008	-0.027	-0.103	0.027	0.049	-0.016	0.000	0.016	0.040	-0.006	-0.014	-0.036	0.109	0.128
cynicism_3	0.024	-0.023	-0.016	0.016	-0.092	0.052	-0.021	-0.011	0.016	0.000	-0.038	-0.019	-0.130	-0.072	-0.009	0.090
exhaustion_1	-0.086	-0.062	-0.021	-0.008	0.102	-0.019	0.141	0.256	0.040	-0.038	0.000	-0.001	-0.031	-0.053	0.098	0.123
exhaustion_2	-0.094	-0.029	-0.066	-0.069	-0.010	-0.131	0.034	0.076	-0.006	-0.019	-0.001	0.000	0.034	0.011	-0.032	-0.002
exhaustion_3	-0.019	-0.030	0.047	0.027	-0.137	-0.109	-0.054	0.032	-0.014	-0.130	-0.031	0.034	0.000	0.052	0.073	0.058
exhaustion_4	-0.022	0.007	0.060	0.064	0.008	0.006	-0.006	0.040	-0.036	-0.072	-0.053	0.011	0.052	0.000	0.011	0.027
score_div100	0.025	-0.053	-0.018	-0.009	-0.025	0.013	0.046	0.068	0.109	-0.009	0.098	-0.032	0.073	0.011	0.000	-0.003
study_year	0.041	-0.004	-0.046	0.009	-0.031	0.059	0.043	0.083	0.128	0.090	0.123	-0.002	0.058	0.027	-0.003	0.000

```r
# The following code chunk won't run on anonymous data.

# full SEM fitted to down-sampled data, residual interitem correlations
all_res_corr <- list()
for (i in 1:n_iterations){
  res_corr <- downsampled_data_residual_correlations[[i]]
  all_res_corr <- append(all_res_corr, res_corr[lower.tri(res_corr)])
}

range(unlist(all_res_corr))
```

```
[1] -0.2256855  0.3143270
```

Table S6: Standardized interitem residual covariances, fitted full model

```
# Table S6A unweighted complete data
res_z = lavResiduals(fit_fullSEM, type = 'raw')$cov.z
range(res_z)
```

```
[1] -2.918249  4.855455
```

Table S6A. Standardized residual covariances, full SEM

	deep_1	deep_2	deep_3	deep_4	unreflective_1	unreflective_2	unreflective_3	cynicism_1	cynicism_2	cynicism_3	exhaustion_1	exhaustion_2	exhaustion_3	exhaustion_4	score_div100	study_year
deep_1	0.00	2.93	-0.17	-0.77	0.04	-0.65	0.79	-0.15	-1.00	0.16	-1.71	-0.51	0.84	-0.28	0.96	1.48
deep_2	2.93	0.00	-2.44	0.52	0.45	0.78	-1.24	-0.49	-0.11	-0.23	-0.79	0.81	0.53	0.31	-1.25	-0.57
deep_3	-0.17	-2.44	0.00	1.14	-0.88	-0.25	0.68	1.17	0.41	-0.23	-0.61	-0.99	2.12	0.33	1.45	1.06
deep_4	-0.77	0.52	1.14	0.00	-0.31	-0.91	0.85	0.22	-0.78	0.33	-0.24	-1.35	1.87	0.20	1.18	1.64
unreflective_1	0.04	0.45	-0.88	-0.31	0.00	1.37	-0.31	0.66	-2.92	-1.57	2.13	-0.35	-1.96	0.17	-0.74	-1.17
unreflective_2	-0.65	0.78	-0.25	-0.91	1.37	0.00	-1.07	0.41	0.12	1.87	0.05	-2.23	-2.23	0.29	-0.04	0.55
unreflective_3	0.79	-1.24	0.68	0.85	-0.31	-1.07	0.00	1.50	1.28	-0.21	2.44	0.89	-1.96	-0.33	-0.40	-0.54
cynicism_1	-0.15	-0.49	1.17	0.22	0.66	0.41	1.50	0.00	-2.26	-1.24	4.86	2.01	0.35	1.14	0.41	0.26
cynicism_2	-1.00	-0.11	0.41	-0.78	-2.92	0.12	1.28	-2.26	0.00	3.19	0.42	-0.51	-0.23	-0.77	1.83	1.28
cynicism_3	0.16	-0.23	-0.23	0.33	-1.57	1.87	-0.21	-1.24	3.19	0.00	-0.68	-0.76	-2.70	-1.72	-1.28	0.08
exhaustion_1	-1.71	-0.79	-0.61	-0.24	2.13	0.05	2.44	4.86	0.42	-0.68	0.00	-0.53	-0.74	-2.14	1.70	1.87
exhaustion_2	-0.51	0.81	-0.99	-1.35	-0.35	-2.23	0.89	2.01	-0.51	-0.76	-0.53	0.00	0.73	1.19	-1.41	-0.92
exhaustion_3	0.84	0.53	2.12	1.87	-1.96	-2.23	-1.96	0.35	-0.23	-2.70	-0.74	0.73	0.00	1.54	1.96	1.19
exhaustion_4	-0.28	0.31	0.33	0.20	0.17	0.29	-0.33	1.14	-0.77	-1.72	-2.14	1.19	1.54	0.00	-0.14	0.07
score_div100	0.96	-1.25	1.45	1.18	-0.74	-0.04	-0.40	0.41	1.83	-1.28	1.70	-1.41	1.96	-0.14	1.38	1.38
study_year	1.48	-0.57	1.06	1.64	-1.17	0.55	-0.54	0.26	1.28	0.08	1.87	-0.92	1.19	0.07	1.38	0.00

```r
# The following code chunk won't run on anonymous data.
# Table S6B weighted data
res_z = lavResiduals(fit_weighted_fullSEM, type = 'raw')$cov.z
range(res_z)
```

```
[1] -3.031664  5.414844
```

Table S6B. Standardized residual covariances, full SEM, weighted data

	deep_1	deep_2	deep_3	deep_4	unreflective_1	unreflective_2	unreflective_3	cynicism_1	cynicism_2	cynicism_3	exhaustion_1	exhaustion_2	exhaustion_3	exhaustion_4	score_div100	study_year
deep_1	0.00	3.02	-0.24	-0.52	-1.11	-0.42	1.09	0.23	-0.42	0.39	-1.34	-1.41	-0.30	-0.35	0.40	0.66
deep_2	3.02	0.00	-1.90	0.53	-0.17	1.38	-0.58	-0.36	0.37	-0.39	-1.10	-0.49	-0.52	0.13	-0.84	-0.06
deep_3	-0.24	-1.90	0.00	0.75	-2.18	-1.47	2.04	0.46	0.30	-0.32	-0.45	-1.44	0.80	1.53	-0.40	-0.68
deep_4	-0.52	0.53	0.75	0.00	-1.28	-0.77	2.40	0.19	-0.68	0.33	-0.16	-1.32	0.45	1.40	-0.18	0.14
unreflective_1	-1.11	-0.17	-2.18	-1.28	0.00	0.85	-0.51	0.82	-2.90	-2.10	2.00	-0.24	-2.96	0.19	-0.49	-0.49
unreflective_2	-0.42	1.38	-1.47	-0.77	0.85	0.00	-0.35	0.89	0.70	1.17	-0.41	-3.03	-2.18	0.13	0.25	0.98
unreflective_3	1.09	-0.58	2.04	2.40	-0.51	-0.35	0.00	2.16	1.28	-0.42	3.12	0.82	-1.12	-0.17	0.85	0.64
cynicism_1	0.23	-0.36	0.46	0.19	0.82	0.89	2.16	0.00	-2.66	-0.73	5.41	1.66	0.62	0.84	1.30	1.30
cynicism_2	-0.42	0.37	0.30	-0.68	-2.90	0.70	1.28	-2.66	0.00	3.38	0.92	-0.14	-0.32	-1.11	2.37	1.99
cynicism_3	0.39	-0.39	-0.32	0.33	-2.10	1.17	-0.42	-0.73	3.38	0.00	-0.82	-0.43	-2.35	-1.53	-0.16	1.35
exhaustion_1	-1.34	-1.10	-0.45	-0.16	2.00	-0.41	3.12	5.41	0.92	-0.82	0.00	-0.03	-1.01	-2.57	1.63	1.85
exhaustion_2	-1.41	-0.49	-1.44	-1.32	-0.24	-3.03	0.82	1.66	-0.14	-0.43	-0.03	0.00	1.11	0.62	-0.60	-0.03
exhaustion_3	-0.30	-0.52	0.80	0.45	-2.96	-2.18	-1.12	0.62	-0.32	-2.35	-1.01	1.11	0.00	1.89	1.25	0.91
exhaustion_4	-0.35	0.13	1.53	1.40	0.19	0.13	-0.17	0.84	-1.11	-1.53	-2.57	0.62	1.89	0.00	0.21	0.41
score_div100	0.40	-0.84	-0.40	-0.18	-0.49	0.25	0.85	1.30	2.37	-0.16	1.63	-0.60	1.25	0.21	-0.41	-0.41
study_year	0.66	-0.06	-0.68	0.14	-0.49	0.98	0.64	1.30	1.99	1.35	1.85	-0.03	0.91	0.41	-0.41	0.00

```r
# The following code chunk won't run on anonymous data.
# full SEM fitted to down-sampled data, standardized residual covariances
all_resz <- list()
for (i in 1:n_iterations){
  res_z <- downsampled_data_standardized_residual_covariances[[i]]
  all_resz <- append(all_resz, res_z[lower.tri(res_z)])
}
range(unlist(all_resz))
```

```
[1] -4.361720  5.287087
```

Table S7. Full model, standardized parameters

- Table S7A: unweighted complete data
- Table S7B: weighted data (This code won't run on the anonymous data.)

```
# Table S7A
STable7A <- standardizedSolution(fit_fullSEM, output = 'data.frame')
STable7A["h2"] <-  NA
STable7A[STable7A$op == "=~", "h2"] <- STable7A[STable7A$op == "=~", "est.std"]^2
tableS7A <- cbind(STable7A[1:3], round(STable7A[4:ncol(STable7A)], 2))
```

Table S7A. Parameter estimates from full SEM fitted on unweighted data

lhs	op	rhs	est.std	se	z	pvalue	ci.lower	ci.upper	h2
f_deep	=~	deep_1	0.50	0.06	8.37	0.00	0.38	0.61	0.25
f_deep	=~	deep_2	0.37	0.07	5.50	0.00	0.24	0.50	0.14
f_deep	=~	deep_3	0.84	0.04	18.60	0.00	0.75	0.92	0.70
f_deep	=~	deep_4	0.81	0.05	17.43	0.00	0.72	0.90	0.66
f_unreflective	=~	unreflective_1	0.62	0.06	11.19	0.00	0.51	0.73	0.39
f_unreflective	=~	unreflective_2	0.53	0.07	8.18	0.00	0.41	0.66	0.28
f_unreflective	=~	unreflective_3	0.68	0.06	11.94	0.00	0.57	0.79	0.46
f_cynicism	=~	cynicism_1	0.70	0.05	15.54	0.00	0.61	0.79	0.49
f_cynicism	=~	cynicism_2	0.86	0.04	23.53	0.00	0.78	0.93	0.73
f_cynicism	=~	cynicism_3	0.74	0.04	18.19	0.00	0.66	0.82	0.54
f_exhaustion	=~	exhaustion_1	0.64	0.05	13.32	0.00	0.55	0.74	0.41
f_exhaustion	=~	exhaustion_2	0.67	0.05	12.62	0.00	0.57	0.78	0.46
f_exhaustion	=~	exhaustion_3	0.53	0.06	8.76	0.00	0.41	0.64	0.28
f_exhaustion	=~	exhaustion_4	0.69	0.05	14.59	0.00	0.60	0.78	0.48
score_div100	~	f_deep	0.16	0.06	2.46	0.01	0.03	0.28	NA
score_div100	~	study_year	0.68	0.03	23.52	0.00	0.62	0.73	NA
score_div100	~	f_unreflective	-0.25	0.11	-2.24	0.03	-0.47	-0.03	NA
score_div100	~	f_cynicism	0.00	0.07	0.01	1.00	-0.14	0.14	NA
score_div100	~	f_exhaustion	0.10	0.10	1.02	0.31	-0.09	0.30	NA
f_cynicism	~~	f_exhaustion	0.54	0.06	8.43	0.00	0.41	0.66	NA
f_unreflective	~~	f_cynicism	0.56	0.08	6.76	0.00	0.39	0.72	NA
f_unreflective	~~	f_exhaustion	0.63	0.07	8.54	0.00	0.49	0.78	NA
f_deep	~~	f_unreflective	-0.29	0.08	-3.53	0.00	-0.46	-0.13	NA
deep_1	~~	deep_1	0.75	0.06	12.77	0.00	0.64	0.87	NA
deep_2	~~	deep_2	0.86	0.05	17.46	0.00	0.77	0.96	NA
deep_3	~~	deep_3	0.30	0.08	4.01	0.00	0.15	0.45	NA
deep_4	~~	deep_4	0.34	0.08	4.55	0.00	0.20	0.49	NA
unreflective_1	~~	unreflective_1	0.61	0.07	8.80	0.00	0.48	0.75	NA
unreflective_2	~~	unreflective_2	0.72	0.07	10.27	0.00	0.58	0.85	NA
unreflective_3	~~	unreflective_3	0.54	0.08	7.03	0.00	0.39	0.69	NA
cynicism_1	~~	cynicism_1	0.51	0.06	8.09	0.00	0.39	0.63	NA
cynicism_2	~~	cynicism_2	0.27	0.06	4.30	0.00	0.15	0.39	NA
cynicism_3	~~	cynicism_3	0.46	0.06	7.68	0.00	0.34	0.57	NA
exhaustion_1	~~	exhaustion_1	0.59	0.06	9.44	0.00	0.46	0.71	NA
exhaustion_2	~~	exhaustion_2	0.54	0.07	7.54	0.00	0.40	0.69	NA
exhaustion_3	~~	exhaustion_3	0.72	0.06	11.51	0.00	0.60	0.85	NA
exhaustion_4	~~	exhaustion_4	0.52	0.07	7.96	0.00	0.39	0.65	NA
score_div100	~~	score_div100	0.45	0.04	11.32	0.00	0.37	0.53	NA
f_deep	~~	f_deep	1.00	0.00	NA	NA	1.00	1.00	NA
f_unreflective	~~	f_unreflective	1.00	0.00	NA	NA	1.00	1.00	NA
f_cynicism	~~	f_cynicism	1.00	0.00	NA	NA	1.00	1.00	NA
f_exhaustion	~~	f_exhaustion	1.00	0.00	NA	NA	1.00	1.00	NA
f_deep	~~	f_cynicism	-0.19	0.09	-2.07	0.04	-0.37	-0.01	NA
f_deep	~~	f_exhaustion	0.08	0.08	1.10	0.27	-0.07	0.23	NA
study_year	~~	study_year	1.00	0.00	NA	NA	1.00	1.00	NA

```r
# The following code chunk won't run on anonymous data.
STable7B <- standardizedSolution(fit_weighted_fullSEM, output = 'data.frame')
```

Table S7B. Parameter estimates from full SEM fitted on weighted data

lhs	op	rhs	est.std	se	z	pvalue	ci.lower	ci.upper	h2
f_deep	=~	deep_1	0.48	0.06	7.46	0.00	0.35	0.60	0.23
f_deep	=~	deep_2	0.34	0.07	4.90	0.00	0.20	0.47	0.11
f_deep	=~	deep_3	0.90	0.05	17.19	0.00	0.79	1.00	0.80
f_deep	=~	deep_4	0.79	0.05	15.32	0.00	0.68	0.89	0.62
f_unreflective	=~	unreflective_1	0.62	0.06	10.92	0.00	0.51	0.73	0.38
f_unreflective	=~	unreflective_2	0.52	0.07	7.77	0.00	0.39	0.65	0.27
f_unreflective	=~	unreflective_3	0.68	0.06	11.45	0.00	0.56	0.79	0.46
f_cynicism	=~	cynicism_1	0.70	0.04	15.88	0.00	0.62	0.79	0.49
f_cynicism	=~	cynicism_2	0.88	0.04	24.15	0.00	0.81	0.96	0.78
f_cynicism	=~	cynicism_3	0.76	0.04	18.65	0.00	0.68	0.84	0.58
f_exhaustion	=~	exhaustion_1	0.65	0.05	13.98	0.00	0.56	0.74	0.42
f_exhaustion	=~	exhaustion_2	0.68	0.05	13.48	0.00	0.58	0.77	0.46
f_exhaustion	=~	exhaustion_3	0.56	0.06	10.13	0.00	0.45	0.67	0.31
f_exhaustion	=~	exhaustion_4	0.74	0.04	16.55	0.00	0.65	0.83	0.55
score_div100	~	f_deep	0.11	0.06	1.70	0.09	-0.02	0.24	NA
score_div100	~	study_year	0.66	0.03	21.26	0.00	0.59	0.72	NA
score_div100	~	f_unreflective	-0.27	0.11	-2.40	0.02	-0.49	-0.05	NA
score_div100	~	f_cynicism	0.03	0.08	0.41	0.68	-0.12	0.18	NA
score_div100	~	f_exhaustion	0.11	0.10	1.08	0.28	-0.09	0.31	NA
f_cynicism	~~	f_exhaustion	0.52	0.06	8.60	0.00	0.40	0.64	NA
f_unreflective	~~	f_cynicism	0.52	0.08	6.35	0.00	0.36	0.68	NA
f_unreflective	~~	f_exhaustion	0.64	0.07	9.09	0.00	0.50	0.78	NA
f_deep	~~	f_unreflective	-0.18	0.09	-2.12	0.03	-0.35	-0.01	NA
deep_1	~~	deep_1	0.77	0.06	12.69	0.00	0.65	0.89	NA
deep_2	~~	deep_2	0.89	0.05	18.96	0.00	0.79	0.98	NA
deep_3	~~	deep_3	0.20	0.09	2.12	0.03	0.01	0.38	NA
deep_4	~~	deep_4	0.38	0.08	4.77	0.00	0.23	0.54	NA
unreflective_1	~~	unreflective_1	0.62	0.07	8.75	0.00	0.48	0.75	NA
unreflective_2	~~	unreflective_2	0.73	0.07	10.64	0.00	0.60	0.87	NA
unreflective_3	~~	unreflective_3	0.54	0.08	6.81	0.00	0.39	0.70	NA
cynicism_1	~~	cynicism_1	0.51	0.06	8.15	0.00	0.38	0.63	NA
cynicism_2	~~	cynicism_2	0.22	0.06	3.37	0.00	0.09	0.35	NA
cynicism_3	~~	cynicism_3	0.42	0.06	6.80	0.00	0.30	0.54	NA
exhaustion_1	~~	exhaustion_1	0.58	0.06	9.65	0.00	0.46	0.70	NA
exhaustion_2	~~	exhaustion_2	0.54	0.07	8.01	0.00	0.41	0.68	NA
exhaustion_3	~~	exhaustion_3	0.69	0.06	11.18	0.00	0.57	0.81	NA
exhaustion_4	~~	exhaustion_4	0.45	0.07	6.74	0.00	0.32	0.58	NA
score_div100	~~	score_div100	0.50	0.04	12.12	0.00	0.42	0.58	NA
f_deep	~~	f_deep	1.00	0.00	NA	NA	1.00	1.00	NA
f_unreflective	~~	f_unreflective	1.00	0.00	NA	NA	1.00	1.00	NA
f_cynicism	~~	f_cynicism	1.00	0.00	NA	NA	1.00	1.00	NA
f_exhaustion	~~	f_exhaustion	1.00	0.00	NA	NA	1.00	1.00	NA
f_deep	~~	f_cynicism	-0.13	0.09	-1.45	0.15	-0.30	0.05	NA
f_deep	~~	f_exhaustion	0.14	0.07	1.94	0.05	0.00	0.28	NA
study_year	~~	study_year	1.00	0.00	NA	NA	1.00	1.00	NA

Table S8: Parameter estimates from measurement models fitted to downsampled data

The following code chunk won't run on anonymous data.

```
n_iterations <- length(downsampled_data_parameter_list)
tableS8 <- downsampled_data_parameter_list[[1]][1:6]
for (i in 2:n_iterations){
  tableS8 <- cbind(tableS8, downsampled_data_parameter_list[[i]][4:6])
}

my_quantile <- function(x, probs){
  return(
    quantile(
      x = x,
      probs = probs ,
      na.rm = TRUE
    )
  )
}

tableS8 <- cbind(
  tableS8[1:3],
  mean.est.std = apply(tableS8[(grepl("est", colnames(tableS8)))], 1, mean),
  t(apply(tableS8[(grepl("est", colnames(tableS8)))], 1, my_quantile, c(0.025, 0.975))),
  mean.se = apply(tableS8[(grepl("se", colnames(tableS8)))], 1, mean),
  t(apply(tableS8[(grepl("se", colnames(tableS8)))], 1, my_quantile, c(0.025, 0.975))),
  mean.z = apply(tableS8[(grepl("z", colnames(tableS8)))], 1, mean),
  t(apply(tableS8[(grepl("z", colnames(tableS8)))], 1, my_quantile, c(0.025, 0.975)))
)
```

Table S8. Parameter estimates from measurement model fitted on downsampled data

lhs	op	rhs	mean.est.std	2.5%	97.5%	mean.se	2.5%	97.5%	mean.z	2.5%	97.5%
f_deep	=~	deep_1	0.49	0.43	0.55	0.07	0.06	0.07	7.35	6.00	8.57
f_deep	=~	deep_2	0.36	0.30	0.41	0.08	0.08	0.09	4.43	3.55	5.28
f_deep	=~	deep_3	0.85	0.80	0.90	0.05	0.04	0.07	16.84	11.71	22.05
f_deep	=~	deep_4	0.79	0.75	0.83	0.06	0.05	0.07	13.35	11.19	15.63
f_unreflective	=~	unreflective_1	0.64	0.59	0.68	0.07	0.06	0.07	9.31	7.95	10.77
f_unreflective	=~	unreflective_2	0.52	0.47	0.58	0.08	0.07	0.09	6.54	5.49	7.70
f_unreflective	=~	unreflective_3	0.67	0.61	0.72	0.07	0.07	0.08	9.32	7.93	10.74
f_cynicism	=~	cynicism_1	0.71	0.67	0.75	0.05	0.05	0.06	13.71	11.74	15.72
f_cynicism	=~	cynicism_2	0.88	0.83	0.92	0.04	0.03	0.05	23.10	17.67	28.79
f_cynicism	=~	cynicism_3	0.75	0.71	0.78	0.05	0.04	0.05	16.05	13.83	18.44
f_exhaustion	=~	exhaustion_1	0.63	0.58	0.67	0.06	0.05	0.06	11.11	9.41	12.94
f_exhaustion	=~	exhaustion_2	0.66	0.62	0.70	0.06	0.06	0.07	10.93	9.20	12.42
f_exhaustion	=~	exhaustion_3	0.58	0.53	0.62	0.07	0.07	0.08	8.18	7.01	9.16
f_exhaustion	=~	exhaustion_4	0.74	0.69	0.79	0.05	0.04	0.06	15.59	12.66	18.64
deep_1	~~	deep_1	0.76	0.70	0.82	0.07	0.06	0.07	11.64	9.69	13.77
deep_2	~~	deep_2	0.87	0.83	0.91	0.06	0.05	0.07	15.24	12.57	18.56
deep_3	~~	deep_3	0.27	0.20	0.35	0.09	0.07	0.11	3.05	2.28	3.74
deep_4	~~	deep_4	0.38	0.31	0.44	0.09	0.08	0.11	4.03	3.17	4.80
unreflective_1	~~	unreflective_1	0.59	0.53	0.65	0.09	0.08	0.09	6.75	5.96	7.62
unreflective_2	~~	unreflective_2	0.73	0.67	0.78	0.08	0.08	0.09	8.74	7.60	10.02
unreflective_3	~~	unreflective_3	0.55	0.48	0.62	0.10	0.09	0.10	5.79	4.79	6.81
cynicism_1	~~	cynicism_1	0.49	0.43	0.56	0.07	0.07	0.08	6.66	5.80	7.58
cynicism_2	~~	cynicism_2	0.22	0.15	0.31	0.07	0.06	0.08	3.25	2.33	4.08
cynicism_3	~~	cynicism_3	0.44	0.39	0.50	0.07	0.07	0.07	6.37	5.69	7.12
exhaustion_1	~~	exhaustion_1	0.60	0.55	0.66	0.07	0.07	0.07	8.50	7.77	9.26
exhaustion_2	~~	exhaustion_2	0.56	0.51	0.62	0.08	0.08	0.09	6.90	6.23	7.61
exhaustion_3	~~	exhaustion_3	0.67	0.62	0.72	0.08	0.08	0.09	8.13	7.27	9.15
exhaustion_4	~~	exhaustion_4	0.45	0.37	0.52	0.07	0.07	0.08	6.28	5.37	7.21
f_deep	~~	f_deep	1.00	1.00	1.00	0.00	0.00	0.00	NA	NA	NA
f_unreflective	~~	f_unreflective	1.00	1.00	1.00	0.00	0.00	0.00	NA	NA	NA
f_cynicism	~~	f_cynicism	1.00	1.00	1.00	0.00	0.00	0.00	NA	NA	NA
f_exhaustion	~~	f_exhaustion	1.00	1.00	1.00	0.00	0.00	0.00	NA	NA	NA
f_deep	~~	f_unreflective	-0.28	-0.36	-0.20	0.10	0.10	0.11	-2.73	-3.62	-1.89
f_deep	~~	f_cynicism	-0.19	-0.27	-0.11	0.11	0.10	0.11	-1.80	-2.54	-1.03
f_deep	~~	f_exhaustion	0.10	0.05	0.16	0.09	0.09	0.10	1.11	0.49	1.74
f_unreflective	~~	f_cynicism	0.51	0.46	0.57	0.10	0.10	0.11	5.02	4.35	5.70
f_unreflective	~~	f_exhaustion	0.59	0.53	0.64	0.09	0.09	0.10	6.34	5.37	7.34
f_cynicism	~~	f_exhaustion	0.50	0.45	0.55	0.07	0.07	0.08	6.81	5.69	7.87

Table S9: Parameter estimates from full SE models fitted to downsampled data

This code chunk won't run on anonymous data.

Table S9. Table S8. Parameter estimates from full SEM fitted on downsampled data

lhs	op	rhs	mean.est.std	2.5%	97.5%	mean.se	2.5%	97.5%	mean.z	2.5%	97.5%
f_deep	=~	deep_1	0.49	0.42	0.55	0.07	0.42	0.55	7.27	5.92	8.51
f_deep	=~	deep_2	0.35	0.29	0.41	0.08	0.29	0.40	4.35	3.46	5.20
f_deep	=~	deep_3	0.87	0.82	0.91	0.05	0.82	0.91	17.95	12.53	23.75
f_deep	=~	deep_4	0.78	0.74	0.82	0.06	0.74	0.81	13.21	11.21	15.38
f_unreflective	=~	unreflective_1	0.64	0.58	0.68	0.07	0.58	0.68	9.48	7.99	11.16
f_unreflective	=~	unreflective_2	0.53	0.47	0.58	0.08	0.47	0.59	6.72	5.64	7.93
f_unreflective	=~	unreflective_3	0.67	0.61	0.72	0.07	0.61	0.72	9.45	8.01	10.91
f_cynicism	=~	cynicism_1	0.71	0.67	0.75	0.05	0.67	0.75	13.72	11.74	15.73
f_cynicism	=~	cynicism_2	0.88	0.83	0.93	0.04	0.83	0.92	23.05	17.64	28.77
f_cynicism	=~	cynicism_3	0.75	0.71	0.78	0.05	0.71	0.78	16.07	13.87	18.51
f_exhaustion	=~	exhaustion_1	0.63	0.58	0.67	0.06	0.58	0.67	11.11	9.38	12.94
f_exhaustion	=~	exhaustion_2	0.66	0.62	0.70	0.06	0.62	0.70	10.92	9.18	12.44
f_exhaustion	=~	exhaustion_3	0.58	0.53	0.62	0.07	0.53	0.62	8.17	7.02	9.17
f_exhaustion	=~	exhaustion_4	0.74	0.69	0.79	0.05	0.70	0.79	15.59	12.63	18.63
score_div100	~	f_deep	0.12	0.04	0.19	0.07	0.04	0.19	1.59	0.57	2.67
score_div100	~	study_year	0.65	0.62	0.67	0.04	0.62	0.67	16.50	14.94	18.09
score_div100	~	f_unreflective	-0.23	-0.32	-0.14	0.13	-0.32	-0.14	-1.81	-2.50	-1.09
score_div100	~	f_cynicism	0.03	-0.04	0.09	0.09	-0.04	0.09	0.29	-0.46	1.04
score_div100	~	f_exhaustion	0.06	-0.01	0.14	0.11	0.00	0.14	0.56	-0.11	1.20
f_cynicism	~~	f_exhaustion	0.50	0.45	0.55	0.07	0.45	0.55	6.81	5.69	7.86
f_unreflective	~~	f_cynicism	0.52	0.46	0.57	0.10	0.45	0.57	5.02	4.36	5.70
f_unreflective	~~	f_exhaustion	0.59	0.53	0.64	0.09	0.53	0.63	6.33	5.37	7.35
f_deep	~~	f_unreflective	-0.28	-0.36	-0.20	0.10	-0.35	-0.21	-2.75	-3.64	-1.95
deep_1	~~	deep_1	0.76	0.70	0.82	0.07	0.70	0.82	11.69	9.69	13.93
deep_2	~~	deep_2	0.88	0.83	0.92	0.06	0.84	0.91	15.66	12.83	19.33
deep_3	~~	deep_3	0.25	0.17	0.33	0.09	0.17	0.33	2.91	2.06	3.63
deep_4	~~	deep_4	0.39	0.33	0.45	0.09	0.34	0.45	4.26	3.41	5.08
unreflective_1	~~	unreflective_1	0.60	0.53	0.66	0.09	0.54	0.66	6.99	6.15	7.88
unreflective_2	~~	unreflective_2	0.72	0.66	0.78	0.08	0.65	0.78	8.70	7.59	9.99
unreflective_3	~~	unreflective_3	0.56	0.48	0.63	0.09	0.48	0.62	5.93	4.97	7.00
cynicism_1	~~	cynicism_1	0.49	0.43	0.56	0.07	0.44	0.55	6.66	5.81	7.58
cynicism_2	~~	cynicism_2	0.22	0.14	0.31	0.07	0.15	0.31	3.26	2.30	4.10
cynicism_3	~~	cynicism_3	0.44	0.39	0.50	0.07	0.39	0.50	6.35	5.68	7.11
exhaustion_1	~~	exhaustion_1	0.60	0.55	0.66	0.07	0.55	0.66	8.49	7.75	9.27
exhaustion_2	~~	exhaustion_2	0.56	0.51	0.62	0.08	0.51	0.62	6.90	6.23	7.61
exhaustion_3	~~	exhaustion_3	0.67	0.62	0.72	0.08	0.61	0.72	8.13	7.27	9.13
exhaustion_4	~~	exhaustion_4	0.45	0.37	0.52	0.07	0.37	0.51	6.27	5.36	7.21
score_div100	~~	score_div100	0.51	0.48	0.54	0.05	0.48	0.54	10.11	8.90	11.21
f_deep	~~	f_deep	1.00	1.00	1.00	0.00	1.00	1.00	NA	NA	NA
f_unreflective	~~	f_unreflective	1.00	1.00	1.00	0.00	1.00	1.00	NA	NA	NA
f_cynicism	~~	f_cynicism	1.00	1.00	1.00	0.00	1.00	1.00	NA	NA	NA
f_exhaustion	~~	f_exhaustion	1.00	1.00	1.00	0.00	1.00	1.00	NA	NA	NA
f_deep	~~	f_cynicism	-0.19	-0.27	-0.11	0.10	-0.27	-0.11	-1.80	-2.53	-1.02
f_deep	~~	f_exhaustion	0.10	0.05	0.16	0.09	0.04	0.16	1.12	0.50	1.75
study_year	~~	study_year	1.00	1.00	1.00	0.00	1.00	1.00	NA	NA	NA

Table S10: Raw score medians by gender

```r
# The following code chunk won't run on anonymous data.
data <- raw_data[,c(2,5:11,13:19, 23, 26:28)]
```

gender	deep_1	deep_2	deep_3	deep_4	unreflective_1	unreflective_2	unreflective_3	cynicism_1	cynicism_2	cynicism_3	exhaustion_1	exhaustion_2	exhaustion_3	exhaustion_4	raw_deep	raw_unreflective	raw_cynicism	raw_exhaustion
2	2	1	3	3	1	3	2	2	1	1	3	2	2	2	9	6	4	9
1	3	3	3	2	0	1	0	0	0	0	1	3	3	3	11	1	0	10
2	2	2	3	3	2	1	1	0	1	1	1	0	3	1	10	4	2	5

```r
# The following code chunk won't run on anonymous data.
stable10 <- cbind(
  males = apply(subset(data, gender == 1), 2, median),
  females = apply(subset(data, gender == 2), 2, median),
  difference = apply(subset(data, gender == 1), 2, median) -
    apply(subset(data, gender == 2), 2, median)
)
```

Table S10. Gender differences in the median values of the items and scale sum scores

	males	females	difference
deep_1	2	2	0
deep_2	2	2	0
deep_3	3	3	0
deep_4	3	3	0
unreflective_1	1	1	0
unreflective_2	1	1	0
unreflective_3	1	1	0
cynicism_1	1	2	-1
cynicism_2	1	1	0
cynicism_3	0	1	-1
exhaustion_1	2	3	-1
exhaustion_2	1	1	0
exhaustion_3	1	2	-1
exhaustion_4	1	2	-1
raw_deep	10	10	0
raw_unreflective	3	4	-1
raw_cynicism	3	3	0
raw_exhaustion	4	8	-4

Figures

Figure 1: Unreflective approach items - information and probability as a function of θ

```
# Information
fit_unreflective_IRTgraded <- mirt(data=raw_data[scale_unreflective4],
                      itemtype = 'graded',
                      verbose = FALSE,
                      model = 1)
summary(fit_unreflective_IRTgraded)
```

```
                  F1    h2
unreflective_1 0.807 0.652
unreflective_2 0.571 0.326
unreflective_3 0.649 0.421
unreflective_4 0.328 0.107

SS loadings:  1.507
Proportion Var:  0.377

Factor correlations:

   F1
F1  1
```

```
plot(fit_unreflective_IRTgraded, type = 'infotrace') # Figure 1 upper row
```

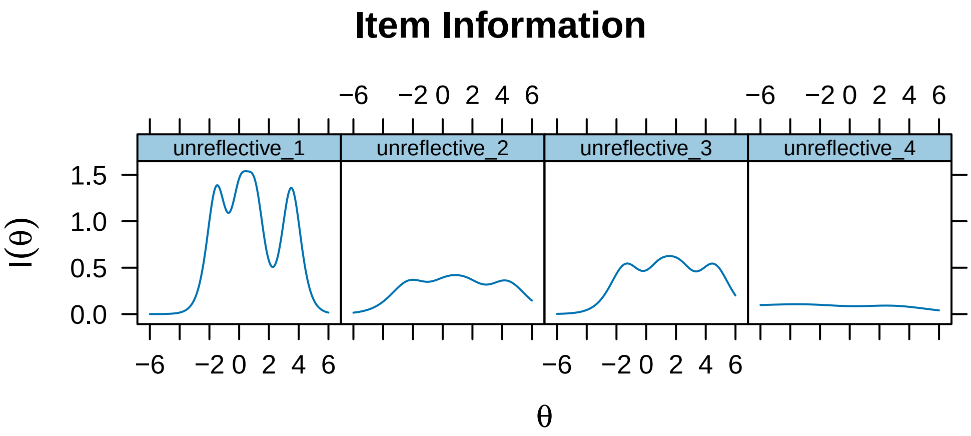

```r
# Probabilities
# Figure 1 lower row, unreflective_1
itemplot(fit_unreflective_IRTgraded, 1, type = 'trace', main = 'unreflective_1')
```

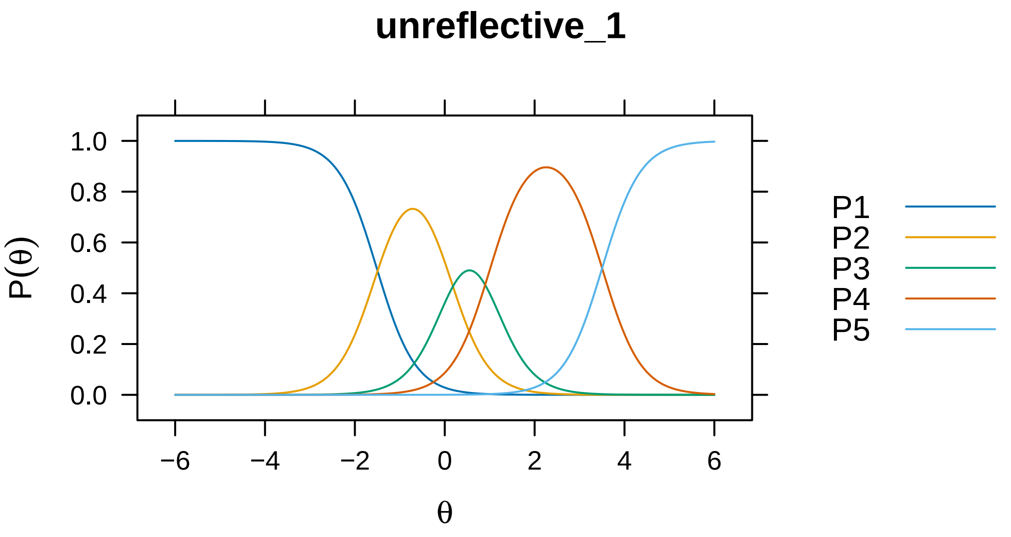


```r
# Figure 1 lower row, unreflective_2
itemplot(fit_unreflective_IRTgraded, 2, type = 'trace', main = 'unreflective_2')
```

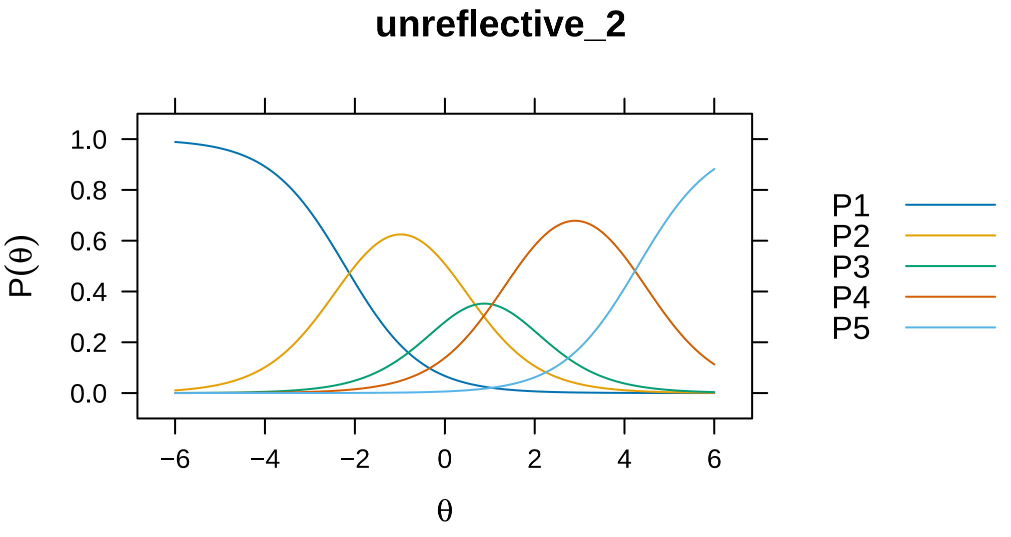

```r
# Figure 1 lower row, unreflective_3
itemplot(fit_unreflective_IRTgraded, 3, type = 'trace', main = 'unreflective_3')
```

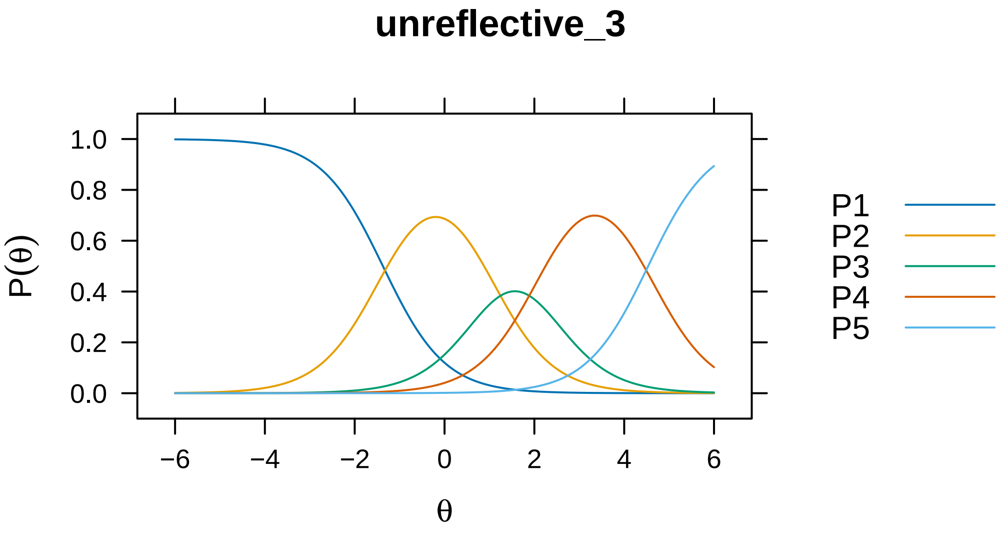


```r
# Figure 1 lower row, unreflective_4
itemplot(fit_unreflective_IRTgraded, 4, type = 'trace', main = 'unreflective_4')
```

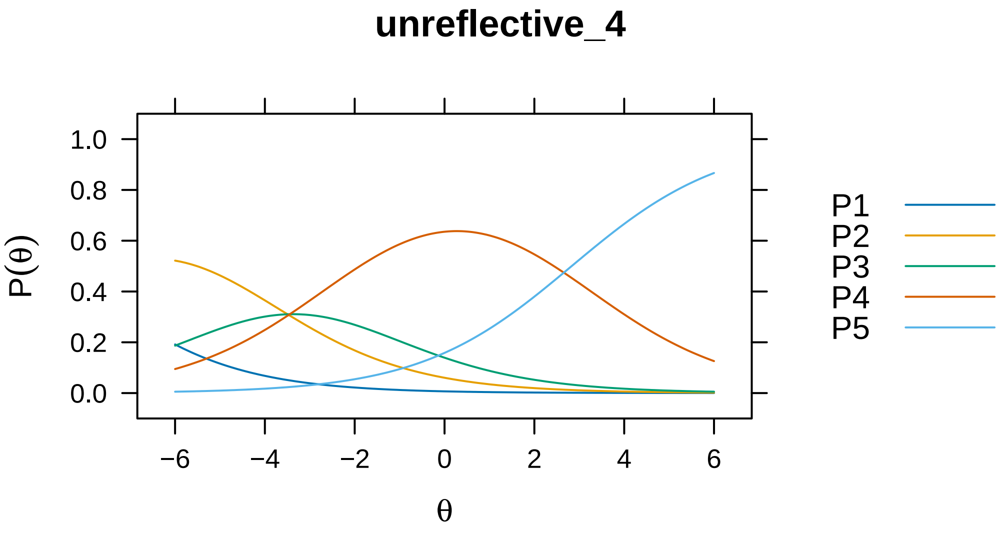

Figure 2: VetRepos test score distribution

```
hist(raw_data['vetrepos_score']) # Figure 2A
```

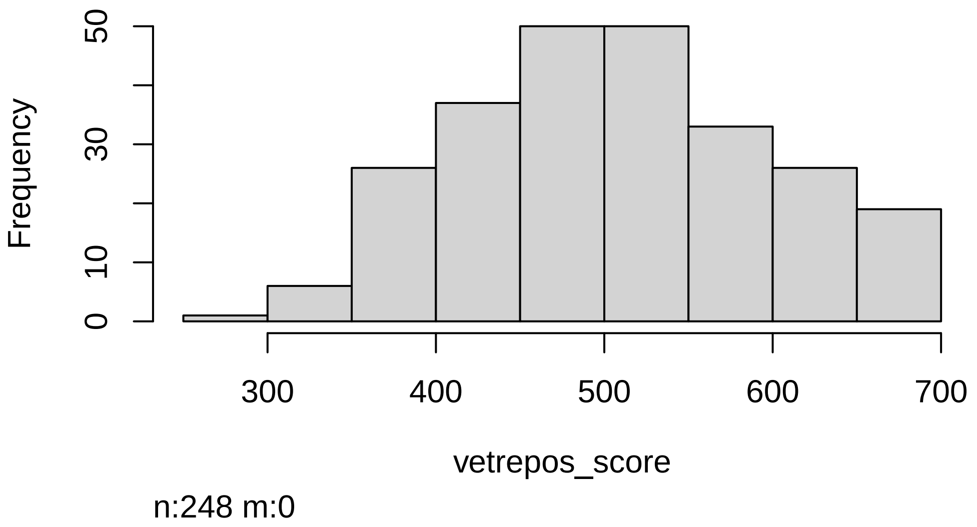


```
# The following code chunk won't run on anonymous data.
boxplot(vetrepos_score ~ study_year, data = raw_data) # Figure 2B
```

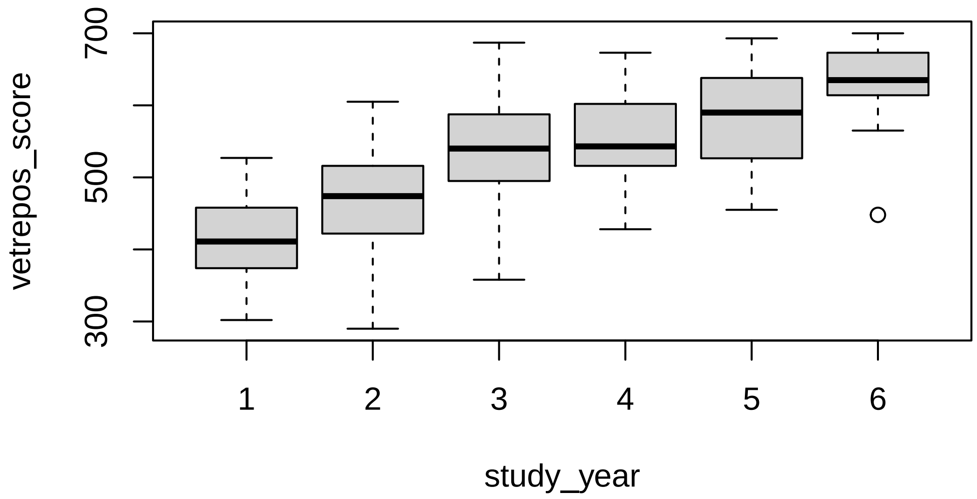


```
# print(kruskal.test(vetrepos_score ~ study_year, data = raw_data))
kruskal.test(vetrepos_score ~ study_year, data = raw_data)
```

```
    Kruskal-Wallis rank sum test

data:  vetrepos_score by study_year
Kruskal-Wallis chi-squared = 124.28, df = 5, p-value < 2.2e-16
```

```
y1 <- raw_data[ raw_data$study_year == 1, 'vetrepos_score']
y2 <- raw_data[ raw_data$study_year == 2, 'vetrepos_score']
y3 <- raw_data[ raw_data$study_year == 3, 'vetrepos_score']
y4 <- raw_data[ raw_data$study_year == 4, 'vetrepos_score']
y5 <- raw_data[ raw_data$study_year == 5, 'vetrepos_score']
y6 <- raw_data[ raw_data$study_year == 6, 'vetrepos_score']

x <- c(y1, y2, y3, y4, y5, y6)
g <- factor(rep(1:6, table(raw_data$study_year)),
            labels = 1:6)
dunn.test(x, g, method = 'bh') # fdr
```

```
  Kruskal-Wallis rank sum test

data: x and g
Kruskal-Wallis chi-squared = 124.2758, df = 5, p-value = 0

                             Comparison of x by g
                             (Benjamini-Hochberg)
Col Mean-|
Row Mean |          1          2          3          4          5
---------+-------------------------------------------------------
       2 |  -3.131104
         |    0.0015*
         |
       3 |  -6.698123  -4.259159
         |    0.0000*    0.0000*
         |
       4 |  -7.465930  -5.162119  -0.959320
         |    0.0000*    0.0000*     0.1807
         |
       5 |  -7.637413  -5.538093  -1.729820  -0.836484
         |    0.0000*    0.0000*     0.0523     0.2014
         |
```

```
       6 |  -7.594304  -5.865184  -2.842328  -2.102684  -1.349013
         |    0.0000*    0.0000*    0.0034*    0.0242*     0.1023

alpha = 0.05
Reject Ho if p <= alpha/2
```

Figure 3: Path diagram for the measurement model

Coefficients from Table S4.

Figure 4: Sum scores of the indicator items

- distributions
- pairwise correlations

```
# pairwise correlation coefficients
data_figure4 <- raw_data[c("raw_deep",
                           "raw_unreflective", # items 1-3
                           "raw_cynicism",
                           "raw_exhaustion")]
```

```
pairs.panels(x = data_figure4,
             method = 'sp',
             digits = 2,
             ci = T
)
```

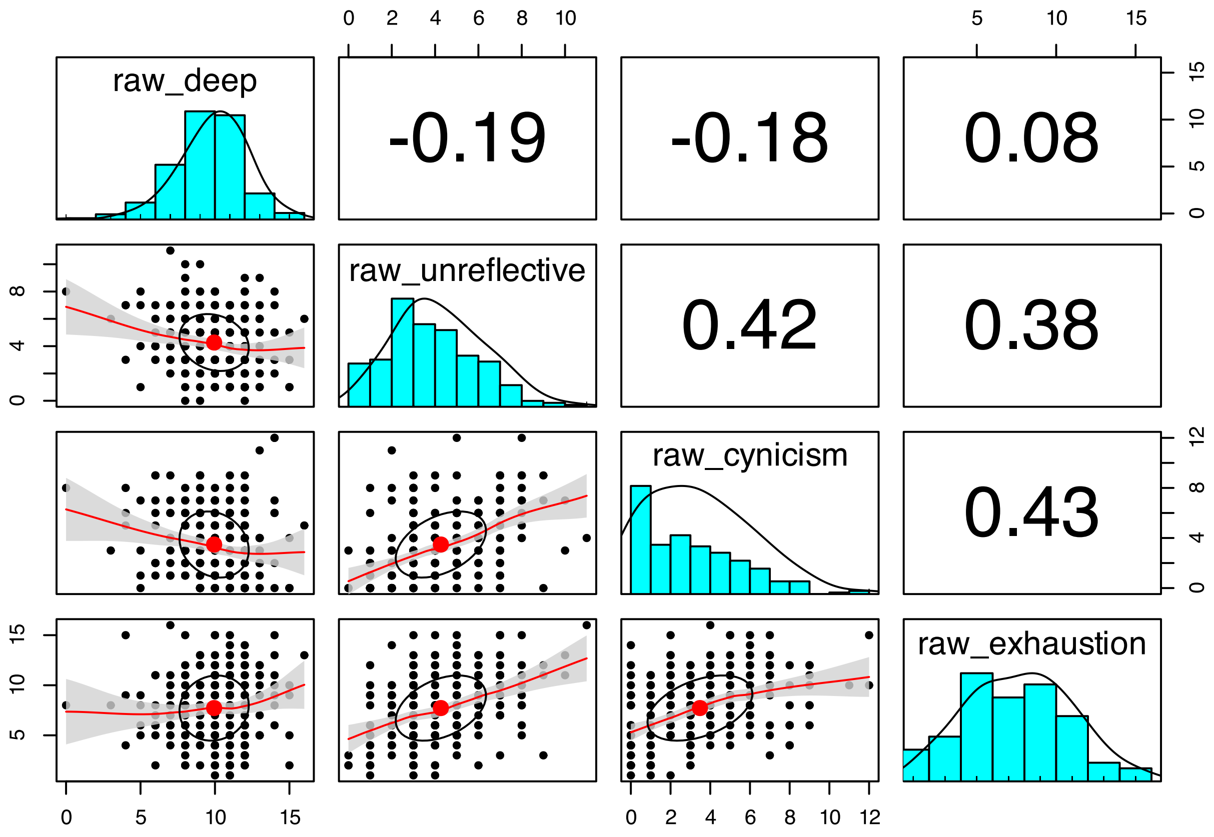


```
# bootstrapping confidence intervals
set.seed(1234)
cor.ci(x = data_figure4, n.iter = n_iterations2, method = "sp", plot = FALSE)
```

```
Call:corCi(x = x, keys = keys, n.iter = n.iter, p = p, overlap = overlap,
    poly = poly, method = method, plot = plot, minlength = minlength,
    n = n)

 Coefficients and bootstrapped confidence intervals
                rw_dp rw_nr rw_cy rw_xh
raw_deep         1.00
raw_unreflective -0.19  1.00
raw_cynicism     -0.18  0.42  1.00
```

```
raw_exhaustion    0.08  0.38  0.43  1.00

 scale correlations and bootstrapped confidence intervals
            lower.emp lower.norm estimate upper.norm upper.emp    p
rw_dp-rw_nr     -0.32      -0.31    -0.19      -0.06     -0.06 0.00
rw_dp-rw_cy     -0.30      -0.30    -0.18      -0.04     -0.04 0.01
rw_dp-rw_xh     -0.04      -0.04     0.08       0.21      0.21 0.18
rw_nr-rw_cy      0.30       0.29     0.42       0.53      0.53 0.00
rw_nr-rw_xh      0.27       0.27     0.38       0.49      0.49 0.00
rw_cy-rw_xh      0.33       0.32     0.43       0.54      0.54 0.00
```

Figure 5: Path diagram for the full model

Coefficients from table S7A.

Figure 6: Standardized residual covariances of the fitted full model

The following code chunk won't run on anonymous data.

Datasets:

- unweighted complete dataset (N=248)
- weighted dataset without university E (N=243)
- down-sampled dataset (N=173, 1000 iterations)
  - all residuals
  - residual between cynicism_1 and exhaustion_1 masked

```
# unweighted dataset
res_z = lavResiduals(fit_fullSEM, type = 'raw')$cov.z
res_z = res_z[lower.tri(res_z)]
data <- data.frame(
  index = 1:length(res_z),
  standardized_residual_covariance = res_z
)
shapiro.test(res_z)
```

```
    Shapiro-Wilk normality test
```

```
data:  res_z
W = 0.98773, p-value = 0.3549
```

```r
# weighted dataset
res_z2 = lavResiduals(fit_weighted_fullSEM, type = 'raw')$cov.z
res_z2 = res_z2[lower.tri(res_z2)]
data2 <- data.frame(
  index = 1:length(res_z2),
  standardized_residual_covariance = res_z2
)

# down-sampled dataset
all_residuals <- list()
all_residuals_masked <- list()
for (i in 1:length(downsampled_data_standardized_residual_covariances)){
  res_z3 <- downsampled_data_standardized_residual_covariances[[i]]
  # all residuals
  all_residuals <- append(all_residuals, res_z3[lower.tri(res_z3)])
  # residual[87] between cynicism_1. and exhaustion_1 is masked
  all_residuals_masked <- append(all_residuals_masked, res_z3[lower.tri(res_z3)][-87])
}

# extraction of residual[87] from the list of all_residuals
res_cyn1exh1 <- unlist(all_residuals)[seq(87, length(all_residuals), length(res_z))]
mean(res_cyn1exh1) #4.435283
```

```
[1] 4.435283
```

```r
range(res_cyn1exh1) # 3.440235 5.287087
```

```
[1] 3.440235 5.287087
```

```r
quantile(res_cyn1exh1, c(0.025, 0.975)) # 3.803168 5.021121
```

```
    2.5%    97.5%
3.803168 5.021121
```

```r
# preparation of dataset for plotting
# all residuals
data3 <- data.frame(
  index = 1:length(unlist(all_residuals)),
  standardized_residual_covariance= unlist(all_residuals)
)
# all residuals with mask on cynicism_1~~exhaustion_1 covariance term
data4 <- data.frame(
  index = 1:length(unlist(all_residuals_masked)),
  standardized_residual_covariance= unlist(all_residuals_masked)
)

# prepare plots
p <- ggplot(data = data3) +
  geom_density(
    aes(
      y = after_stat(scaled),
      x = standardized_residual_covariance
    ),
    fill = 'lightblue',
    lwd = 0
  ) +
  geom_density(
    data = data4,
    aes(
      y = after_stat(scaled),
      x = standardized_residual_covariance
    ),
    fill = 'lightgreen',
    lwd = 2,
    linetype = 'dotted'
  ) +
  geom_density(
    data = data3,
    aes(
      y = after_stat(scaled),
      x = standardized_residual_covariance,
    ),
    lwd = 1
  ) +
```

```
  scale_y_continuous(
    name = "relative density",
    sec.axis = sec_axis(
      transform = ~ . * max(data$index) / 1,
      name = "index",
      breaks = seq(0, 120, 30)
    )
  ) +
  geom_point(
    data = data2,
    aes(
      x = standardized_residual_covariance,
      y = index / max(index)
    ),
    color = "brown",
    alpha = 0.9,
    size = 3
  ) +
  geom_point(
    data = data,
    aes(
      x = standardized_residual_covariance,
      y = index / max(index)
    ),
    color = 'blue',
    alpha = 0.85,
    size = 2
  ) +
  scale_x_continuous(limits = c(-6, 6)) +
  labs(x = "standardized residual covariance")  +
  theme_minimal() &
  theme(
    text = element_text(size = 12),
    axis.text = element_text(size = 12)
  )
p
```

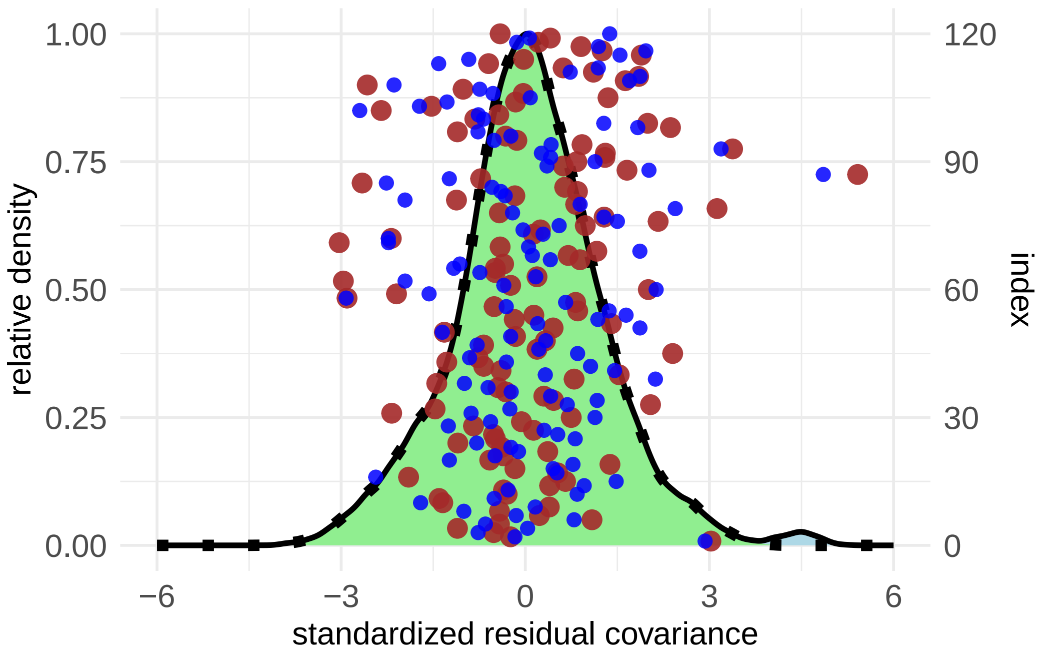

Figure 7: Comparison of parameter estimates from full SE models

The following code chunk won't run on anonymous data.

Full SE model:

- unweighted complete dataset (N=248)
- weighted dataset without university E (N=243)
- down-sampled dataset (N=173, 1000 iterations)
  - mean values of parameter estimates fetched from Table S9

Full SE model with freely estimated covariance between cynicism_1 and exhaustion_1

- unweighted complete dataset (N=248)

```
# unweighted data
data <- standardizedSolution(fit_fullSEM, output='data.frame')
# downsampled_mean_value from Table S9
data$ds_mean_value <- tableS9$mean.est.std
# weighted data
data$wt_est.std <- standardizedSolution(
  fit_weighted_fullSEM,
```

```r
  output='data.frame'
)$est.std

# SEM with covariance term cynicism_1~~exhaustion_1
# cynicism_1 ~~ exhaustion_1 covariance term excluded to match line numbers
data$cov_est.std <- standardizedSolution(
  fit_fullSEM_cov,
  output='data.frame'
)$est.std[-24]

data <- subset(data, se != 0)

# preparation of the labels
estimates = list()
for (i in 1:nrow(data)){
  estimates <- append(estimates, paste(c(t(data[i,1:3])), collapse = ''))
}

data$estimate <- unlist(estimates)
data[nrow(data)+1,'estimate'] <- 'factor loadings'
data[nrow(data)+1,'estimate'] <- 'variances'
data[nrow(data)+1,'estimate'] <- 'regression coefficients'
data[nrow(data)+1,'estimate'] <- 'correlation coefficients'

estimates <- '
factor loadings
f_deep=~deep_1
f_deep=~deep_2
f_deep=~deep_3
f_deep=~deep_4
f_unreflective=~unreflective_1
f_unreflective=~unreflective_2
f_unreflective=~unreflective_3
f_cynicism=~cynicism_1
f_cynicism=~cynicism_2
f_cynicism=~cynicism_3
f_exhaustion=~exhaustion_1
f_exhaustion=~exhaustion_2
f_exhaustion=~exhaustion_3
f_exhaustion=~exhaustion_4
```

```
regression coefficients
score_div100~f_deep
score_div100~study_year
score_div100~f_unreflective
score_div100~f_cynicism
score_div100~f_exhaustion

correlation coefficients
f_deep~~f_cynicism
f_deep~~f_exhaustion
f_deep~~f_unreflective
f_unreflective~~f_cynicism
f_unreflective~~f_exhaustion
f_cynicism~~f_exhaustion

variances
deep_1~~deep_1
deep_2~~deep_2
deep_3~~deep_3
deep_4~~deep_4
unreflective_1~~unreflective_1
unreflective_2~~unreflective_2
unreflective_3~~unreflective_3
cynicism_1~~cynicism_1
cynicism_2~~cynicism_2
cynicism_3~~cynicism_3
exhaustion_1~~exhaustion_1
exhaustion_2~~exhaustion_2
exhaustion_3~~exhaustion_3
exhaustion_4~~exhaustion_4
score_div100~~score_div100
'

estimates <- strsplit(x = estimates, split = "\n")[[1]]
estimates <- estimates[estimates != ""]
data$estimate <- factor(data$estimate, levels=rev(estimates))

tick_labels <- rev(data[with (data, order (estimate)), 'rhs'])

# (1:nrow(data))[is.na(tick_labels)] # 1, 16, 22, 29
```

```r
tick_labels[1] <- "FACTOR LOADINGS"
tick_labels[16] <- "VETREPOS SCORE REGRESSIONS"
tick_labels[22] <- "CORRELATIONS"
tick_labels[29] <- "VARIANCES"
tick_labels[23:28] <- estimates[23:28]
tick_labels[nrow(data)] <- "score_div100"

# preparation of the plot
p1 <- ggplot(
  data = data,
  aes(x=estimate, xend=estimate, y=ci.lower, yend=ci.upper)
)

p2 <- p1 +
  theme_minimal() +
  theme(
    axis.text = element_text(color = 'black', size = 12),
    axis.line = element_line(color='black'),
    axis.ticks = element_line(color = 'black'),
    panel.grid.minor = element_blank(),
    panel.grid.major.y = element_line(linetype = 'dotted', colour = 'gray'),
    panel.grid.major.x = element_blank(),
    panel.ontop = FALSE
  ) +
  geom_hline(yintercept = 0, linetype = 'dashed', color = 'gray') +
  geom_segment(color="darkgrey", linewidth=1) +
  geom_point(aes(x=estimate, y=est.std), color = "darkgrey", size = 2) +
  geom_point(aes(x=estimate, y=wt_est.std), color = "brown", shape = "I", size = 7) +
  geom_point(aes(x=estimate, y=cov_est.std), color = "black", shape = "I", size = 6) +
  geom_point(aes(x=estimate, y=ds_mean_value), color = "blue", shape = "I", size = 5) +
  coord_flip() +
  labs(y = 'standardized value', x = '') +
  scale_x_discrete(label = rev(tick_labels))

ggsave("figure7.svg", p2, height = 8, width = 9)
rsvg_pdf(svg = "figure7.svg", file = "figure7.pdf", width = 900, height = 800)
knitr::include_graphics(path = "figure7.pdf", dpi = 450)
```

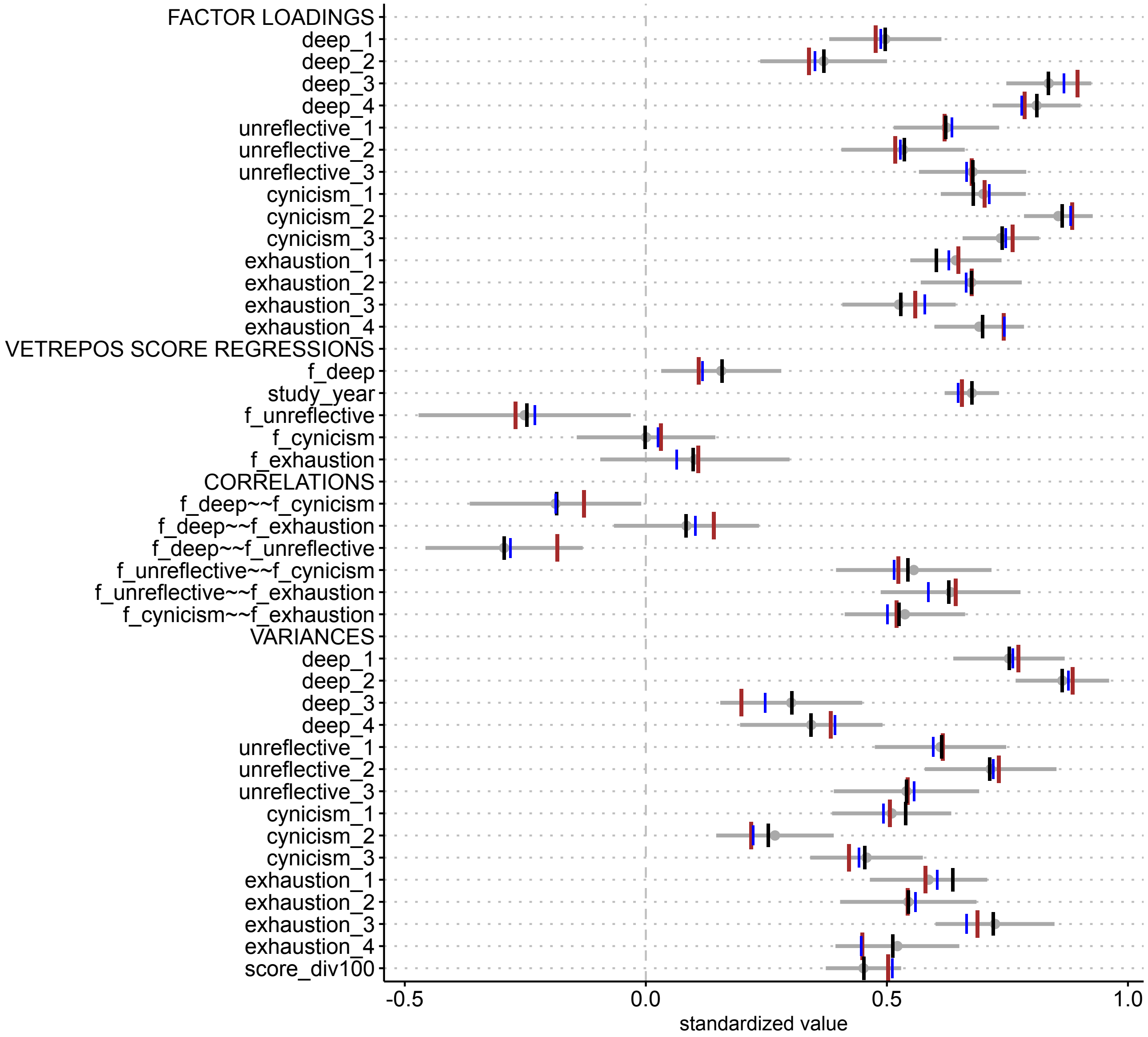

Figure 8: Levels of latent traits across study years

The following code chunk won't run on anonymous data.

```
svg("figure8.svg", height = 4, width =9)
ydims = c(-2,2.2)
```

```r
layout(
  mat = matrix(c(1,2,3,4), nrow = 1),
  widths = c(1,1,1,1),
  heights = c(1.5,1.5,1.5,1.5),
  respect = TRUE
)
boxplot(
  f_deep ~ study_year,
  complete_data, ylab = 'latent_trait_level',
  main='Deep approach',
  ylim=ydims
)
boxplot(
  f_unreflective ~ study_year,
  complete_data, ylab = 'latent_trait_level',
  main='Unreflective approach',
  ylim=ydims
)
boxplot(
  f_cynicism ~ study_year,
  complete_data,
  ylab = 'latent_trait_level',
  main='Cynicism',
  ylim=ydims
)
boxplot(
  f_exhaustion ~ study_year,
  complete_data,
  ylab = 'latent_trait_level',
  main='Exhaustion',
  ylim=ydims
)

dev.off()
```

```r
rsvg_pdf(svg = "figure8.svg", file = "figure8.pdf", width = 900, height = 400)
knitr::include_graphics("figure8.pdf")
```

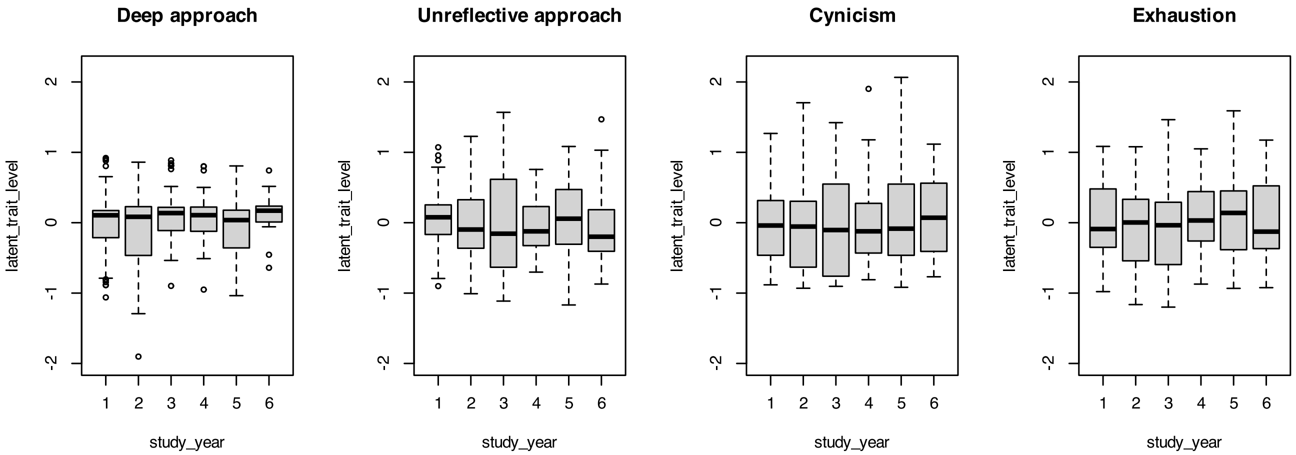


```r
### statistical analysis
# Mardia test failed
mardiaKurtosis(complete_data[c("f_deep", "f_unreflective", "f_cynicism", "f_exhaustion")])
```

```
        b2d           z          p
25.99481143  2.26713339  0.02338208
```

```r
mardiaSkew(complete_data[c("f_deep", "f_unreflective", "f_cynicism", "f_exhaustion")])
```

```
         b1d          chi           df            p
1.828695e+00 7.558606e+01 2.000000e+01 2.174916e-08
```

```r
# 1-way anova
kruskal.test(f_deep ~ study_year, complete_data) # NS
```

```

    Kruskal-Wallis rank sum test

data:  f_deep by study_year
Kruskal-Wallis chi-squared = 6.0169, df = 5, p-value = 0.3046
```

```
kruskal.test(f_unreflective ~ study_year, complete_data) # NS
```

```

    Kruskal-Wallis rank sum test

data:  f_unreflective by study_year
Kruskal-Wallis chi-squared = 4.8859, df = 5, p-value = 0.43
```

```
kruskal.test(f_cynicism ~ study_year, complete_data) # NS
```

```

    Kruskal-Wallis rank sum test

data:  f_cynicism by study_year
Kruskal-Wallis chi-squared = 1.3497, df = 5, p-value = 0.9297
```

```
kruskal.test(f_exhaustion ~ study_year, complete_data) # NS
```

```

    Kruskal-Wallis rank sum test

data:  f_exhaustion by study_year
Kruskal-Wallis chi-squared = 2.6436, df = 5, p-value = 0.7547
```

Figure 9: Standardized levels of latent traits

- distributions
- correlations

```
set.seed(1234)
svg(filename = "figure9.svg", width = 7, height = 5)
pairs.panels(lavaan_traits,
             method = 'sp',
             digits = 2,
             rug = FALSE,
             ci = TRUE) # Figure 8
dev.off()
```

```
rsvg_pdf(svg = "figure9.svg", file = "figure9.pdf", width = 700, height = 500)
knitr::include_graphics(path = "figure9.pdf")
```

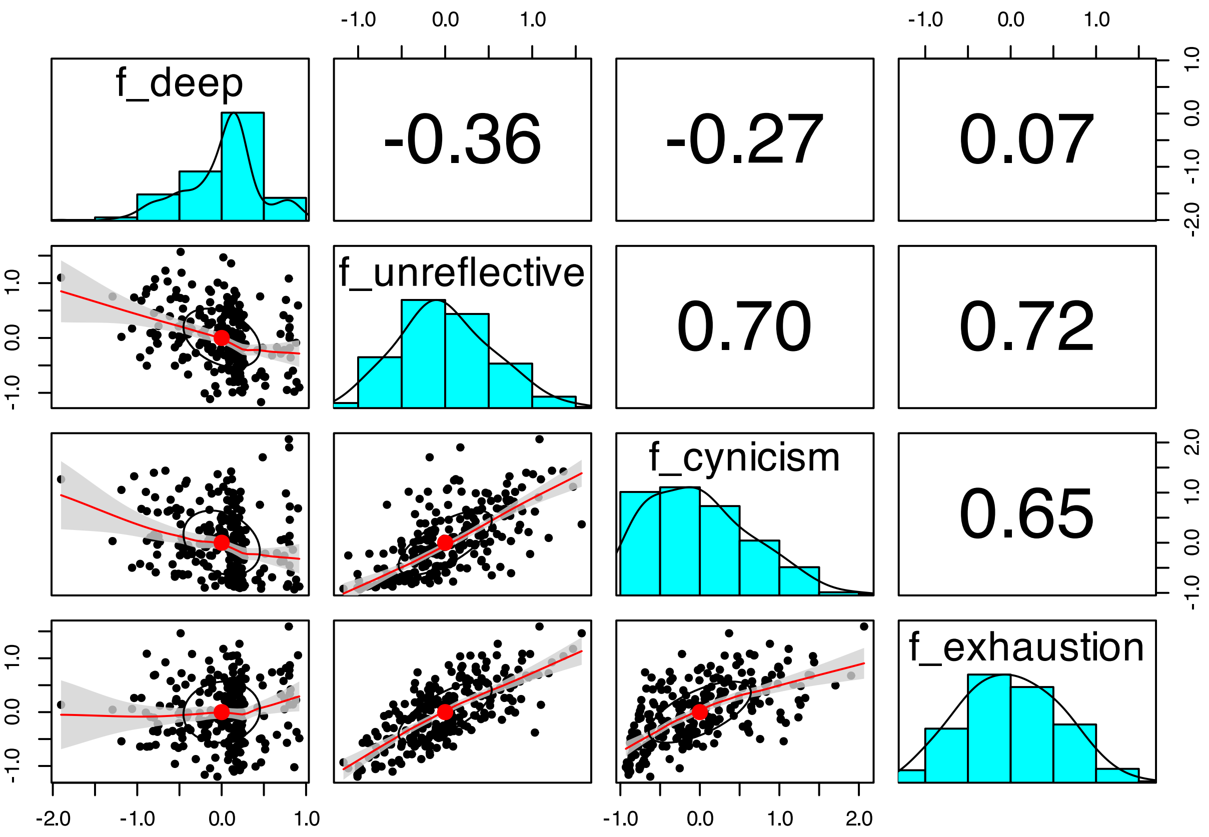


```
set.seed(1234)
cor.ci(
  lavaan_traits,
  n.iter = n_iterations2,
  method = 'sp',
  plot = FALSE
)
```

```
Call:corCi(x = x, keys = keys, n.iter = n.iter, p = p, overlap = overlap,
```

```
    poly = poly, method = method, plot = plot, minlength = minlength,
    n = n)

 Coefficients and bootstrapped confidence intervals
              f_dep f_nrf f_cyn f_xhs
f_deep         1.00
f_unreflective -0.36  1.00
f_cynicism     -0.27  0.70  1.00
f_exhaustion    0.07  0.72  0.65  1.00

 scale correlations and bootstrapped confidence intervals
            lower.emp lower.norm estimate upper.norm upper.emp    p
f_dep-f_nrf     -0.48      -0.47    -0.36      -0.25     -0.25 0.00
f_dep-f_cyn     -0.38      -0.38    -0.27      -0.14     -0.14 0.00
f_dep-f_xhs     -0.06      -0.06     0.07       0.20      0.20 0.26
f_nrf-f_cyn      0.61       0.61     0.70       0.77      0.77 0.00
f_nrf-f_xhs      0.66       0.65     0.72       0.78      0.78 0.00
f_cyn-f_xhs      0.56       0.56     0.65       0.72      0.72 0.00
```

Figure S1: Model comparison: original vs. covariance term at cynicism_1~~exhaustion_1

```
data <- cbind(
  standardizedSolution(fit_fullSEM, output='data.frame'),
  # covariance term is removed to match data length
  standardizedSolution(fit_fullSEM_cov, output='data.frame')[-24,]
)

# estimated value of the covariance term from the output of standardizedSolution()
#               lhs op            rhs est.std    se      z pvalue ci.lower ci.upper
# 24     cynicism_1 ~~   exhaustion_1   0.308 0.064  4.847  0.000    0.184    0.433


colnames(data)[10:18] <- paste('cov.', colnames(data)[10:18], sep = '')

data <- subset(data, se != 0)

# preparation of labels
estimates = list()
for (i in 1:nrow(data)){
```

```
  estimates <- append(estimates, paste(c(t(data[i,1:3])), collapse = ''))
}

data$estimate <- unlist(estimates)
data[nrow(data)+1,'estimate'] <- 'factor loadings'
data[nrow(data)+1,'estimate'] <- 'variances'
data[nrow(data)+1,'estimate'] <- 'regression coefficients'
data[nrow(data)+1,'estimate'] <- 'correlation coefficients'

estimates <- '
factor loadings
f_deep=~deep_1
f_deep=~deep_2
f_deep=~deep_3
f_deep=~deep_4
f_unreflective=~unreflective_1
f_unreflective=~unreflective_2
f_unreflective=~unreflective_3
f_cynicism=~cynicism_1
f_cynicism=~cynicism_2
f_cynicism=~cynicism_3
f_exhaustion=~exhaustion_1
f_exhaustion=~exhaustion_2
f_exhaustion=~exhaustion_3
f_exhaustion=~exhaustion_4

regression coefficients
score_div100~f_deep
score_div100~study_year
score_div100~f_unreflective
score_div100~f_cynicism
score_div100~f_exhaustion

correlation coefficients
f_deep~~f_cynicism
f_deep~~f_exhaustion
f_deep~~f_unreflective
f_unreflective~~f_cynicism
f_unreflective~~f_exhaustion
f_cynicism~~f_exhaustion
```

```
variances
deep_1~~deep_1
deep_2~~deep_2
deep_3~~deep_3
deep_4~~deep_4
unreflective_1~~unreflective_1
unreflective_2~~unreflective_2
unreflective_3~~unreflective_3
cynicism_1~~cynicism_1
cynicism_2~~cynicism_2
cynicism_3~~cynicism_3
exhaustion_1~~exhaustion_1
exhaustion_2~~exhaustion_2
exhaustion_3~~exhaustion_3
exhaustion_4~~exhaustion_4
score_div100~~score_div100
'

estimates <- strsplit(x = estimates, split = "\n")[[1]]
estimates <- estimates[estimates != ""]
data$estimate <- factor(data$estimate, levels=rev(estimates))

tick_labels <- rev(data[with (data, order (estimate)), 'rhs'])

# (1:nrow(data))[is.na(tick_labels)]
tick_labels[1] <- "FACTOR LOADINGS"
tick_labels[16] <- "VETREPOS SCORE REGRESSIONS"
tick_labels[22] <- "CORRELATIONS"
tick_labels[29] <- "VARIANCES"
tick_labels[23:28] <- estimates[23:28]
tick_labels[nrow(data)] <- "score_div100"

# preparation of the plot
p_cleveland <- ggplot(data = data) +
  geom_segment(
    aes(x=estimate, xend=estimate, y=ci.lower, yend=ci.upper),
    color='gray',
    linewidth=2
  ) +
  geom_hline(yintercept = 0, linetype = 'dashed', color = 'gray') +
```

```
geom_point( aes(x=estimate,y=ci.lower), color="darkgrey", shape='I', size = 6) +
geom_point(aes(x=estimate, y=est.std), color = "darkgrey", shape='I', size = 8) +
geom_point(aes(x=estimate, y=ci.upper), color = "darkgrey", shape='I', size = 6) +

geom_segment(
  aes(x=estimate, xend=estimate, y=cov.ci.lower, yend=cov.ci.upper),
  color='red',
  linewidth=1,
  linetype = 'dotted'
) +
geom_point( aes(x=estimate,y=cov.ci.lower), color="red", shape='I', size = 4) +
geom_point(aes(x=estimate, y=cov.est.std), color = "red", shape='I', size = 6) +
geom_point(aes(x=estimate, y=cov.ci.upper), color = "red", shape='I', size = 4) +

coord_flip() +
theme_minimal() +
theme(
  text = element_text(size = 12, color = 'black'),
  axis.text = element_text(color = 'black', size = 12),
  axis.line = element_line(color='black'),
  axis.ticks = element_line(color = 'black'),
  panel.grid.minor = element_blank(),
  panel.grid.major.y = element_line(linetype = 'dotted', colour = 'gray'),
  panel.grid.major.x = element_blank(),
  panel.ontop = FALSE
) +
labs(y = 'standardized value', x = '' ) +
scale_x_discrete(label = rev(tick_labels))
```

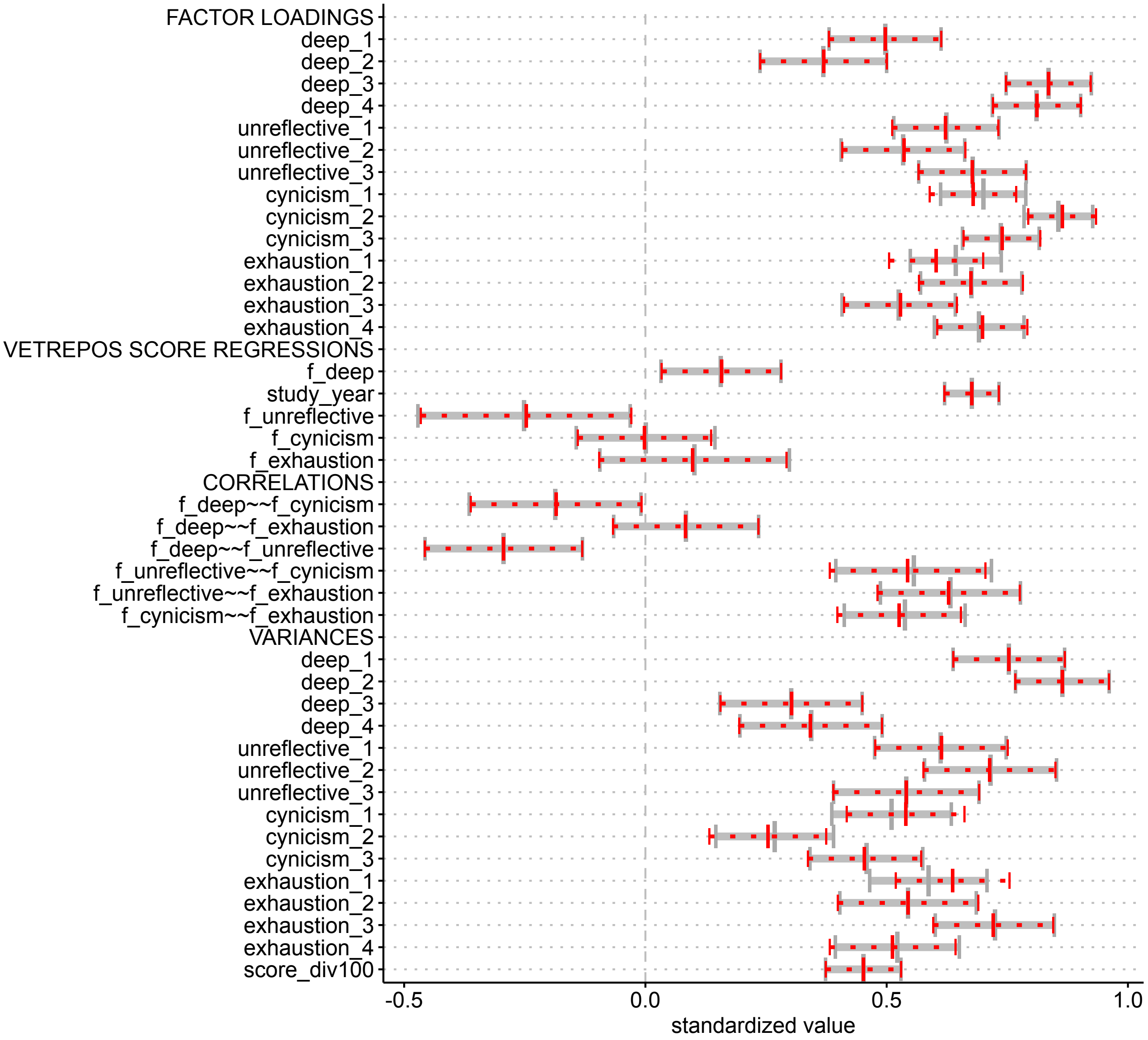

Figure S2: Raw sumscores of learning approaches, cynicism and exhaustion in males and females

The following code chunk won't run on anonymous data.

```r
data <- raw_data

p1 <- ggplot(data = data) +
  geom_histogram(
    aes(
      x = raw_deep,
      # color = gender == 1,
      fill = gender == 1,
      # alpha = 0.4
    ),
  position = 'identity',
  alpha = 0.4,
  bins = 17
) +
theme_minimal() +
theme(
  text = element_text(size = 12),
  axis.line = element_line(color='black'),
  axis.ticks = element_line(color = 'black'),
  panel.grid.minor = element_blank(),
  panel.grid.major = element_blank(),
  panel.ontop = TRUE
)

p2 <- ggplot(data = data) +
  geom_histogram(aes(x = raw_unreflective,
                     # color = gender == 1,
                     fill = gender == 1),
                 position = 'identity', alpha = 0.4, bins = 12) +
  theme_minimal() +
  theme(
    text = element_text(size = 12),
    axis.line = element_line(color='black'),
    axis.ticks = element_line(color = 'black'),
    panel.grid.minor = element_blank(),
    panel.grid.major = element_blank(),
    panel.ontop = TRUE
  )
```

```
p3 <- ggplot(data = data) +
  geom_histogram(aes(x = raw_cynicism,
                     # color = gender == 1,
                     fill = gender == 1),
                 position = 'identity', alpha = 0.4, bins = 13) +
  theme_minimal() +
  theme(
    text = element_text(size = 12),
    axis.line = element_line(color='black'),
    axis.ticks = element_line(color = 'black'),
    panel.grid.minor = element_blank(),
    panel.grid.major = element_blank(),
    panel.ontop = TRUE
  )


p4 <- ggplot(data = data) +
  geom_histogram(aes(x = raw_exhaustion,
                     # color = gender == 1,
                     fill = gender == 1),
                 position = 'identity', alpha = 0.4, bins = 16) +
  theme_minimal() +
  theme(
    text = element_text(size = 12),
    axis.line = element_line(color='black'),
    axis.ticks = element_line(color = 'black'),
    panel.grid.minor = element_blank(),
    panel.grid.major = element_blank(),
    panel.ontop = TRUE
  )

p_all <- (p1 | p2  ) / (p3  | p4) +
  plot_annotation(
    title = 'Raw sum scores of the scales in male and female students',
    tag_levels = 'A'
  ) +
  plot_layout(guides = 'collect')  &
  scale_fill_manual(
    name = "gender",
    values = c("TRUE" = rgb(0,0,1,0.1), "FALSE" = rgb(1,0,0,0.1)),
```

```
    labels = c("female", "male"),
    # alpha = 0.3
  )


ggsave("figureS2.svg", p_all, height = 7, width = 10)
p_all
```

Raw sum scores of the scales in male and female students

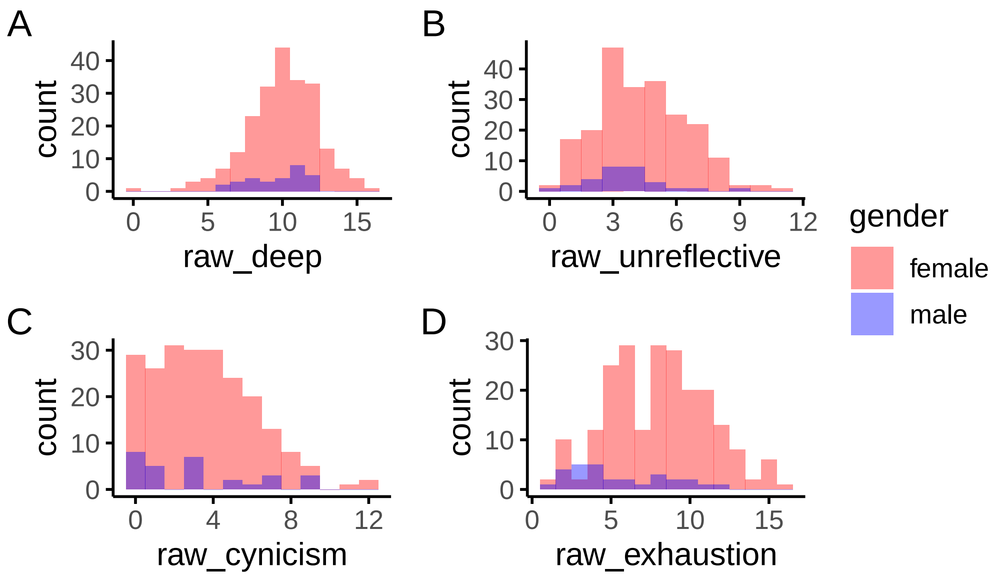


```
# statistical analysis with fdr adjustment for p-value (n = 4)
kruskal.test(
  x = c(
    subset(data, gender == 1)["raw_deep"],
    subset(data, gender == 2)["raw_deep"]
  )
)$p.value # NS 0.451
```

```
[1] 0.4509818
```

```
p.adjust(kruskal.test(
  x = c(
```

```
    subset(data, gender == 1)["raw_unreflective"],
    subset(data, gender == 2)["raw_unreflective"]
  )
)$p.value, n = 4, 'fdr') # NS = 0.1988
```

```
[1] 0.1988126
```

```
kruskal.test(
  x = c(
    subset(data, gender == 1)["raw_cynicism"],
    subset(data, gender == 2)["raw_cynicism"]
  )
)$p.value # NS
```

```
[1] 0.2575851
```

```
kt <- kruskal.test(
  x = c(
    subset(data, gender == 1)["raw_exhaustion"],
    subset(data, gender == 2)["raw_exhaustion"]
  )
)

kt
```

```
    Kruskal-Wallis rank sum test

data:  c(subset(data, gender == 1)["raw_exhaustion"], subset(data, gender == 2)["raw_exhaustion"]
Kruskal-Wallis chi-squared = 14.8, df = 1, p-value = 0.0001195
```

```
p.adjust(kt$p.value, n=4, 'fdr')
```

```
[1] 0.0004781081
```
